# Exploring a Role for Regulatory miRNAs In Wound Healing during Ageing:Involvement of miR-200c in wound repair

**DOI:** 10.1038/s41598-017-03331-6

**Published:** 2017-06-12

**Authors:** Eerik Aunin, David Broadley, Mohammed I. Ahmed, Andrei N. Mardaryev, Natalia V. Botchkareva

**Affiliations:** 1 Centre for Skin Sciences, University of Bradford, Bradford, West Yorkshire UK; 2 School of Science and Technology, Nottingham Trent University, Nottingham, UK

## Abstract

Multiple factors and conditions can lead to impaired wound healing. Chronic non-healing wounds are a common problem among the elderly. To identify microRNAs negatively impacting the wound repair, global miRNA profiling of wounds collected from young and old mice was performed. A subset of miRNAs that exhibited an age-dependent expression pattern during wound closure was identified, including miR-31 and miR-200c. The expression of miR-200 family members was markedly downregulated upon wounding in both young and aged mice, with an exception of acute upregulation of miR-200c at the early phase of wound healing in aged skin. In unwounded aged skin (versus unwounded younger skin), the level of miR-200c was also found elevated in both human and mice. Overexpression of miR-200c in human *ex vivo* wounds delayed re-epithelialisation and inhibited cell proliferation in the wound epithelium. Modulation of miR-200c expression in both human and mouse keratinocytes *in vitro* revealed inhibitory effects of miR-200c on migration, but not proliferation. Accelerated wound closure *in vitro* induced by anti-miR-200c was associated with upregulation of genes controlling cell migration. Thus, our study identified miR-200c as a critical determinant that inhibits cell migration during skin repair after injury and may contribute to age-associated alterations in wound repair.

## Introduction

Wound healing is a complex process that aims to repair skin integrity and functions, and can be divided into four phases: haemostasis, inflammation, proliferation and remodelling. Each phase overlaps with another and is characterised by coordinated activation and interplay of the cutaneous residential and migratory cells regulated by an array of signalling pathways, including platelet-derived growth factor (PDGF), transforming growth factor beta (TGF-β), vascular endothelial growth factor (VEGF), stromal cell-derived factor 1 (SDF-1) and fibroblast growth factors (FGF)^1–5^. Multiple factors and conditions can lead to impaired wound healing. Chronic wound healing disorders are a common problem among the elderly^6, 7^. In healthy older individuals, acute wound healing is temporally delayed as well. Ageing affects multiple processes, including DNA repair, mitochondrial function, cell cycle, proteolysis and cellular metabolism^8, 9^. Ageing-related impairment in wound repair is associated with alterations in all major components of healing process^7, 10^.

A class of non-coding RNA molecules that have recently emerged as critical factors in wound healing are the microRNAs (miRNAs)^11–13^. miRNAs are about 22-25 nucleotides in length and affect a wide range of cellular processes. miRNAs function by inhibiting mRNA translation or by targeting mRNA for degradation^14–17^. Increasing evidence suggest that miRNAs regulate gene expression program and outcome during wound healing. For example, cutaneous wound repair is compromised in both keratinocyte-specific and endothelial-specific Dicer-ablated mice, which asserts that miRNAs are implicated in keratinocyte differentiation and angiogenesis in the healing process^18, 19^. The possible involvement of miRNAs in skin wound healing has been illuminated by several expression profiling studies that have found differential expression of many miRNAs in wounded skin^18, 20–27^. Some notable examples include miR-130a, miR-132, miR-155, miR-198, miR-21, miR-31 and miR-378a^13, 23, 24, 26, 28–30^. miR-155 acts as an important player in controlling the inflammatory response during skin repair; genetic deletion of miR-155 in mice leads to accelerated healing associated with elevated numbers of macrophages and increased type-1 collagen deposition in wounded tissue^30^. TGF-β inducible miR-132 and miR-31 were found to be upregulated during the transition from the inflammatory to the proliferative phase in human skin promoting keratinocyte proliferation^23, 28^. In contrast, miR-378a negatively affects the remodelling phase, delaying the healing of mouse skin wounds by downregulating β3 integrin and vimentin^29^.

The Tomic-Canic lab identified a candidate set of microRNAs that contributes to the chronic non-healing wounds^13^. They observed enhanced levels of miR-21 and miR-130 in venous ulcers patients, which delay healing of human wounds by targeting leptin receptor (LepR)^13^.

Although miRNAs have emerged as key players in skin repair, their contribution to the aged-associated changes in the skin and impairment in wound healing remains unknown. The objective of the current study was to identify expressional changes of miRNAs during wound healing in aged versus young skin using mouse model, and to define the role for distinct miRNAs in the control of keratinocyte proliferation, migration and differentiation that might contribute to the age-associated alterations in cutaneous wound healing.

## Results and Discussion

### Age-dependent changes in miRNA expression fluctuation during cutaneous wound healing

**Figure 1:**
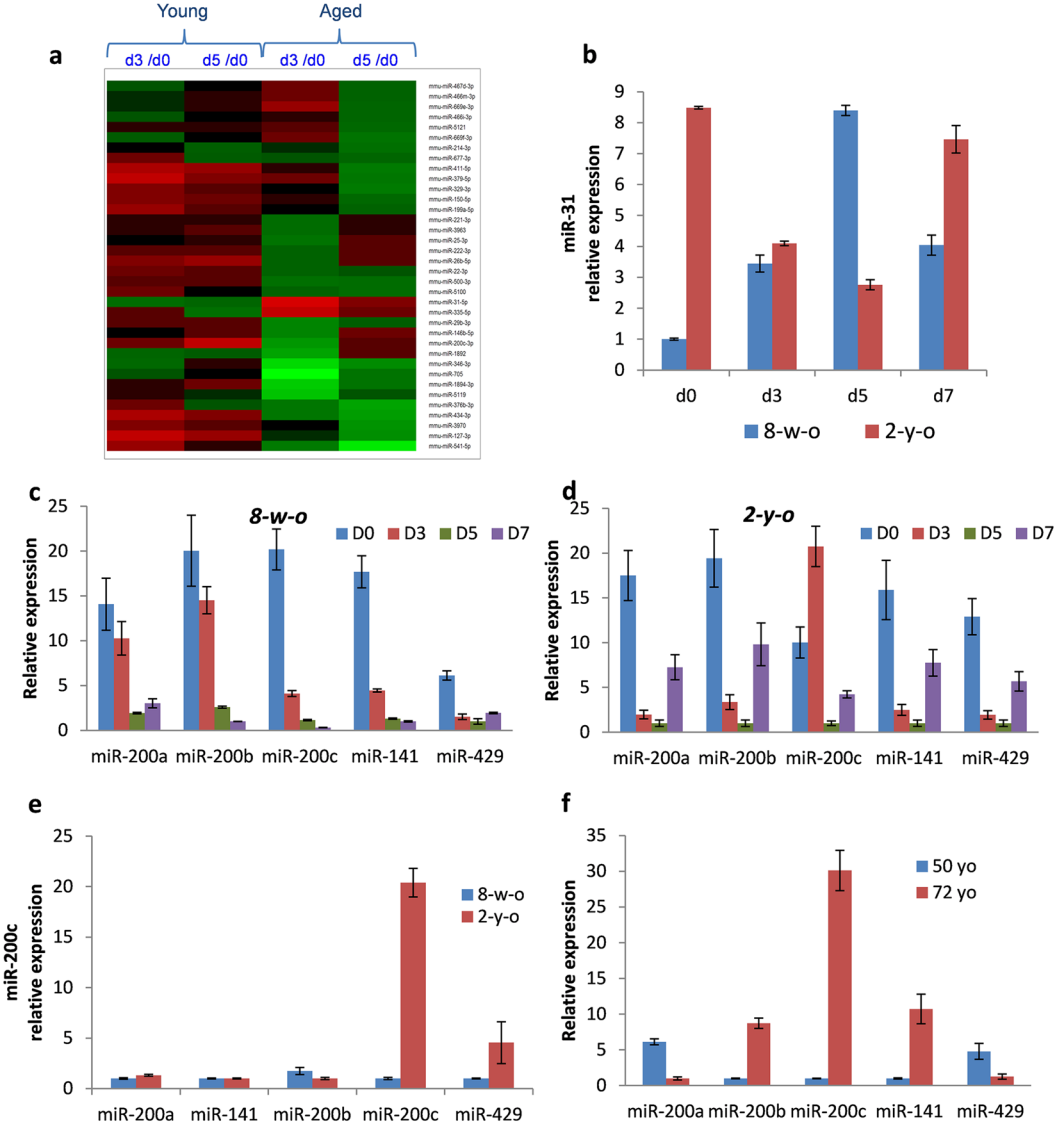
A differentially expressed subset of miRNAs in young versus aged skin during wound healing. (**a**) Microarray: heat map represents miRNAs that exhibited opposite dynamic of their expression (with at least 2-fold differences in fold changes) in young versus aged mouse wounds at days 3 and 5 (d3 and d5) post wounding compared to unwounded skin (d0). (Green: high expression; red: low expression); (**b**) RT-qPCR: increased miR-31 expression in 2-year-old versus 8-week-old intact mouse back skin, and increase in miR-31 levels during wound healing in the skin of 8-week-old mice in contrast to the skin of 2-year-old mice; (**c**) RT-qPCR: decreased expression of all miR-200 family members on day 3, 5, and 7 after wounding in the skin of 8-week-old mice; (**d**) RT-qPCR: decreased expression of miR-200 family after wounding, except for upregulation of miR-200c expression on day 3 after wounding in the skin of 2-year-old mice; (**e**) RT-qPCR: markedly elevated levels of miR-200c in intact telogen skin of 2-year-old versus 8-week-old mice; (**f**) RT-qPCR: prominent elevation in miR-200c expression in the epidermis of a healthy 72-year-old donor versus 50-year-old individual.

In order to identify the candidate miRNAs that might compromise wound healing and contribute to the age-associated delay in wound repair, global miRNA profiling was performed in mouse back telogen skin of young (8-week-old) and aged (2-year-old) animals at distinct time points after wounding (Supplementary Table 1a,b). Bioinformatics analysis revealed 37 miRNAs that exhibited opposite changes with at least 2-fold differences in their levels at days 3 and 5 (D3 and D5) post wounding compared to unwounded skin (day 0; D0) (Fig. 1a, Table 1). Such age-dependent differential expression of selected miRNAs might suggest their involvement in ageing-associated changes in cutaneous wound repair. Interestingly, microarray validation by RT-qPCR confirmed the contrasting expression of miR-31. Significantly increased levels of miR-31 in unwounded aged versus (vs) young skin were decreased on days 3 and 5 after injury, in contrast to increase in miR-31 expression in young mice on days 3 and 5 post-wounding (Fig. 1b, Supplementary Tables 1 and 2). The observed changes in the dynamics of miR-31 levels in aged skin during wound healing suggest that miR-31 may compromise wound repair in aged skin. miR-31 is known to be highly expressed in the activated keratinocytes under hyperproliferative conditions, including anagen phase of the hair cycle, psoriasis, and cutaneous squamous cell carcinoma^31–35^. Moreover, our finding reconciles well with the previous reports about the positive impact of miR-31 on re-epithelialisation during acute wound healing^28^. miR-31 expression has been shown to be gradually increased in the epithelial tongue and promotes keratinocyte proliferation and migration during wound repair^28^. Therefore, diminished expression of miR-31 post-wounding in aged skin may suppress keratinocyte proliferation. In addition, the observed decreased miR-31 levels in aged skin may also contribute to the changes in the inflammatory response, as this miRNA has been found to positively regulate inflammatory cytokine and chemokine production in primary human keratinocytes^35^.

**Table 1:** Subset of differentially expressed miRNAs in young versus aged skin during wound healing (microarray data).

Reporter Name	Young D3/D0	Young D5/D0	Aged D3/D0	Aged D5/D0
mmu-miR-705	1.2	1	6	2
mmu-miR-541-5p	0.5	0.9	2.2	5.4
mmu-miR-1894-3p	0.9	0.7	4.5	2
mmu-miR-5119	0.9	1.1	4.3	1.2
mmu-miR-346-3p	1.7	0.9	4.9	2.7
mmu-miR-434-3p	0.4	0.6	2.1	3.2
mmu-miR-127-3p	0.3	0.5	1.1	2.8
mmu-miR-200c-3p	0.7	0.3	3	0.8
mmu-miR-376b-3p	0.7	1.2	2.1	3.4
mmu-miR-3970	0.6	0.8	1	2.9
mmu-miR-1892	1.6	1.3	3.4	0.8
mmu-miR-411-5p	0.4	0.5	0.9	2.2
mmu-miR-29b-3p	0.8	0.8	2.5	1.4
mmu-miR-31-5p	1.8	1.6	0.2	0.6
mmu-miR-379-5p	0.3	0.6	0.7	2.1
mmu-miR-146b-5p	1	0.8	2.5	0.7
mmu-miR-329-3p	0.6	0.8	1	2.2
mmu-miR-335-5p	0.8	1.9	0.3	0.7
mmu-miR-500-3p	0.8	0.8	1.9	1.8
mmu-miR-669f-3p	1.3	1	0.7	2
mmu-miR-150-5p	0.6	0.7	0.9	1.7
mmu-miR-25-3p	1	0.9	2	0.8
mmu-miR-221-3p	0.9	0.9	1.8	0.9
mmu-miR-3963	0.9	0.8	1.8	0.9
mmu-miR-26b-5p	0.6	0.5	1.5	0.8
mmu-miR-5100	0.7	1	1.5	1.8
mmu-miR-22-3p	0.7	0.8	1.5	1.2
mmu-miR-5121	0.9	0.9	0.8	1.7
mmu-miR-669e-3p	1.1	0.9	0.5	1.6
mmu-miR-199a-5p	0.5	0.8	1	1.5
mmu-miR-222-3p	0.8	0.8	1.5	0.8
mmu-miR-466i-3p	1.2	1	0.9	1.6
mmu-miR-466m-3p	1.1	0.9	0.7	1.5
mmu-miR-214-3p	1	1.4	1.1	1.9
mmu-miR-677-3p	0.7	1.3	1.2	1.6
mmu-miR-467d-3p	1.2	1	0.7	1.5

We also observed that the expression of all members of miR-200 family was markedly downregulated in both young and aged mouse wounds (Fig. 1c,d, Supplementary Table 1a,b), with the exception of acute upregulation of miR-200c observed at day 3 of wound healing in aged skin (Fig. 1d, Table 1). Interestingly, it was previously reported that the first phases of healing are delayed in aged wounds with significant decline in the rate of re-epithelialisation that takes place at day 3 after wounding^7, 36^.

In addition to the transient upregulation of miR-200c during wound healing in aged mouse skin, miR-200c expression was significantly increased in the intact unwounded skin of 2-year-old mice in contrast to 8 week-old mice (Fig. 1e, Supplementary Table 2). Similarly, miR-200c levels are higher in the human aged epidermis (Fig. 1f). Due to the altered levels of miR-200c in aged skin and during early stages of wound healing in old mice we selected this miRNA for further investigation.

There are multiple reasons to suggest that miR-200c may be involved in wound healing. The miR-200 family consists of epithelial-specific miRNAs that are known to function as negative regulators of epithelial to mesenchymal transition (EMT) by targeting E-cadherin transcriptional repressors, zinc finger E-box-binding homeobox 1 (ZEB1) and ZEB2^37^. EMT is an important process that also occurs in wound healing, and is required for keratinocyte activation and their migration across the wound bed^38^. This fact makes miR-200 family members potential candidates for the regulation of re-epithelialisation during wound healing. Our data suggest that the increased expression of miR-200c in aged skin could contribute to the impaired skin repair associated with ageing and might be implicated in the pathogenesis of chronic wounds.

### miR-200c regulates keratinocyte migration and differentiation

**Figure 2:**
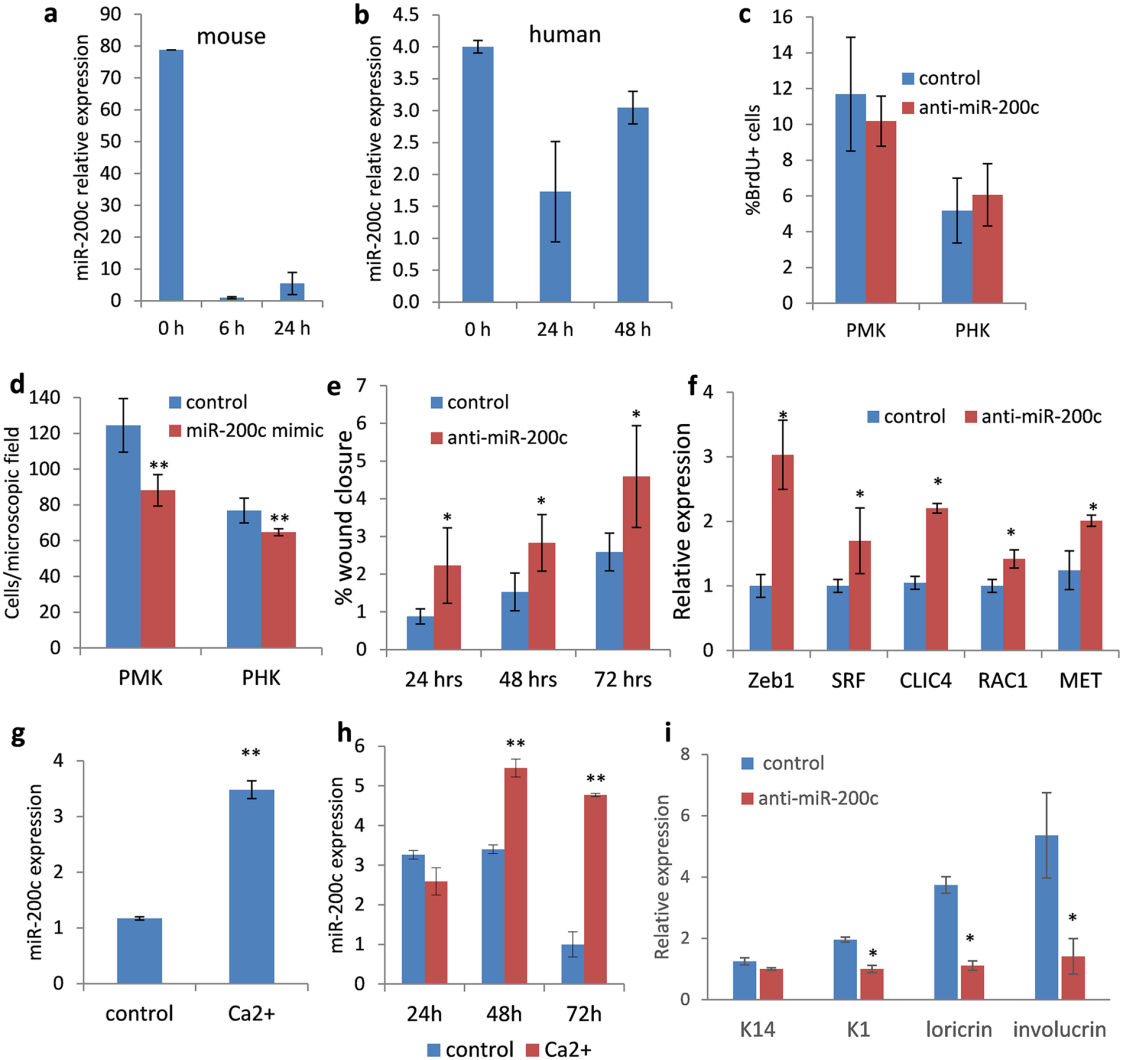
miR-200c exerts negative effects on keratinocyte migration and positive effect on keratinocyte differentiation. (**a**) RT-qPCR: dramatic decrease in miR-200c expression in primary mouse keratinocytes during scratch wound healing assay *in vitro*; (**b**) RT-qPCR: downregulation of miR-200c expression in primary human keratinocytes during scratch wound healing assay *in vitro*; (**c**) Lack of difference in the number of BrdU+ cells in both mouse (PMK) and human (PHK) keratinocytes transfected with miR-200c inhibitor versus scrambled RNA control; (**d**) miR-200c mimic suppresses migration of both human and mouse keratinocytes. Transwell migration assay: primary mouse or human keratinocytes were transfected with miR-200c mimic or negative control RNA and allowed to migrate for 48 hours (n = 3; mean ± SE; **p < 0.001; Wilcoxon rank sum test); (**e**) Scratch assay: primary mouse keratinocytes transfected with miR-200c inhibitor exhibited accelerated migration compared to the negative control (n = 3; mean ± SD; *p < 0.05; Student’s t test); (**f**) RT-qPCR: Accelerated wound closure induced by anti-miR-200c is associated with upregulation of genes controlling cell migration, including *Zeb1, Srf, Clic4, Rac1, Met* in mouse keratinocytes treated with miR-200c inhibitor (n = 3; mean ± SD; *p < 0.05; Student’s t test); (**g**) Ca^2+^-induced differentiation in mouse primary keratinocytes; increase in the expression of miR-200c 48 hours after Ca^2+^ treatment (n = 3, mean ± SD; **p < 0.01; Student’s t test); (**h**) Ca^2+^-induced differentiation in human keratinocytes; increase in the expression of miR-200c at 48 h and 72 h after Ca^2+^ treatment (n = 3, mean ± SD; **p < 0.01; Student’s t test); (**i**) Ca^2+^-induced differentiation in mouse keratinocytes; downregulation of *Loricrin* and *Involucrin* expression in primary mouse keratinocytes transfected with miR-200c inhibitor 48 hours after Ca^2+^ treatment (n = 3, mean ± SD; *p < 0.05; Student’s t test).

Keratinocyte migration, proliferation and differentiation are all critical components of successful re-epithelialisation during wound healing^4, 5, 39^. To begin to elucidate the potential effects of miR-200c on keratinocyte activity during wound healing, *in vitro* “scratch” assay was performed using primary mouse and human keratinocytes. Similar to *in vivo* observations, miR-200c expression was downregulated in both primary mouse and human keratinocytes during closure of scratch-induced wounds (Fig. 2a,b). To investigate the functional significance of miR-200c in keratinocytes, loss and gain of function experiments were employed. Possible effects of miR-200c on keratinocyte proliferation were evaluated by transfecting primary mouse and human epidermal keratinocytes with miR-200c inhibitor for 48 hours followed by quantitative analysis of bromodeoxyuridine (BrdU) positive cells. No significant difference in the proliferation rate was detected in anti-miR-200c treated and corresponding control groups in both mouse and human keratinocytes (Fig. 2c). Consistently, quantification of Ki-67 positive cells in the primary mouse keratinocytes 24 hours after transfection with miR-200c mimic also revealed no effect of miR-200c on keratinocyte proliferation (Supplementary Figure 1a,b). Moreover, fluorescence-activated cell sorting (FACS) analysis of human immortalised keratinocytes, HaCaT cells, transfected with miR-200c mimic did not show any changes in proliferation in response to the increased levels of miR-200c (Supplementary Figure 1c).

However, miR-200c mimic significantly reduced primary mouse and human keratinocyte migration in transwell assay (Fig. 2d), while inhibition of miR-200c in keratinocytes resulted in significant acceleration of their migration in scratch assay (Fig. 2e). Moreover, accelerated wound closure induced by anti-miR-200c in scratch assay was associated with upregulation of *Zeb1*, serum response factor (*Srf*), chloride intracellular channel 4 (*Clic4*), RAS-related C3 botulinum toxin substrate 1 (*Rac1*) and hepatocyte growth factor receptor (*Met*) (Fig. 2f). All these genes have previously been shown to be involved in the control of cell migration during cutaneous wound healing^40–43^ and, more importantly, are potential target genes of miR-200c identified by the TargetScan software^44^.

Next, we examined the involvement of miR-200c in keratinocyte differentiation. Keratinocyte differentiation was induced in both primary mouse and human keratinocytes *in vitro* by their exposure to high calcium medium^45^; it was associated with significant upregulation in the expression of miR-200c (Fig. 2g,h). Transfection of mouse keratinocytes with miR-200c inhibitor resulted in decreased expression of differentiation-associated genes, such as *Krt1*, *Lor* and *Ivl*, while the expression of *Krt14*, a marker of undifferentiated keratinocytes, was not affected by miR-200c inhibition (Fig. 2g). Thus, miR-200c can exert stimulatory effects on epidermal differentiation that is also known to be essential for proper wound healing. Indeed, it has been demonstrated that there is incomplete activation and deregulated differentiation of keratinocytes in human chronic wounds^46, 47^. Specifically, the expression of early differentiation markers is suppressed, whereas late differentiation markers such as involucrin and transglutaminase 1 are upregulated in venous ulcers, when compared to healthy skin^46^. Therefore, the aberrant expression of miR-200c in the epithelial edges of chronic wounds (Fig. 1g) could have a negatively impact not only on keratinocyte migration, but may also interfere with their differentiation.

Taken together, these data suggest that miR-200c can be involved in the regulation of different aspects of wound healing, sustaining keratinocyte differentiation and inhibiting their migration.

### miR-200c compromises wound healing in human *ex vivo* skin

**Figure 3:**
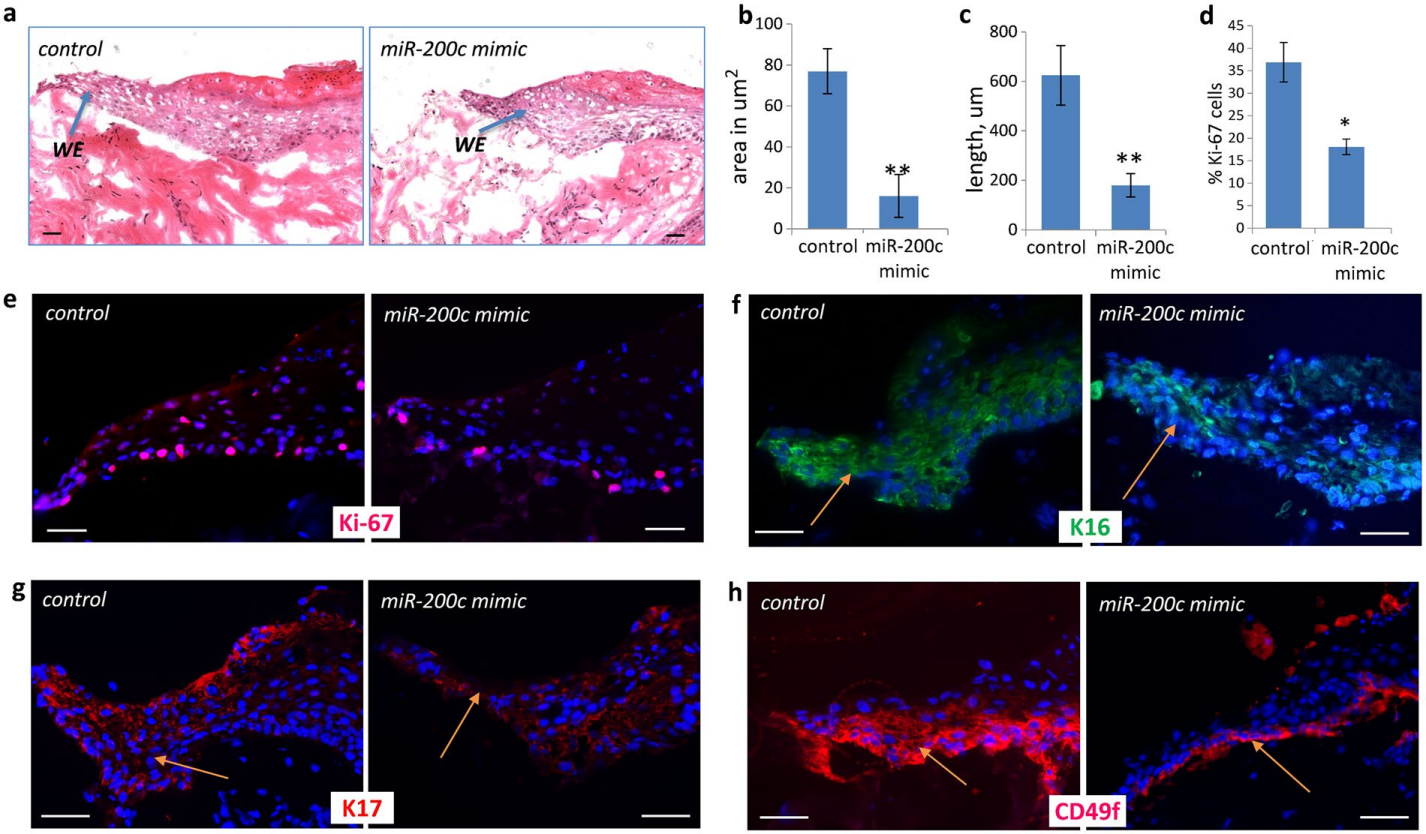
miR-200c delays wound healing in human skin *ex vivo*. (**a**) Representative images of wound histology of miR-200c mimic and negative control RNA treated skins 5 days post-wounding (H&E staining, WE – wound epithelium, scale bar 50 µm); (**b**) Significantly reduced area of wound epithelium in miR-200c mimic treated wound on day 5 after wounding versus the control (n = 5; mean ± SE, ***p < 0.0001, Student’s t-test); (**c**) Significantly reduced wound epithelial tongue length in miR-200c mimic treated wounds on day 5 post-wounding (n = 5; mean ± SE, ***p < 0.0001, Student’s t-test); (**d–e)** Ki-67+ proliferating keratinocytes seen in the wound epithelial tongue of *ex vivo* human wounds following 5 days of miR-200c mimic treatment (scale bars 50 μm); significantly decreased percentage of Ki-67+ proliferating cells in the wound epithelium after 5 days of miR-200c mimic treatment compared to the control (n = 4; mean ± SE; *p < 0.05; Student’s t test). (**f**) Reduced expression of Keratin 16 in miR-200c mimic treated wounds compared to the control (arrows; scale bar 50 µm); (**g**) Decreased expression of Keratin 17 in miR-200c mimic treated wounds compared to the control (arrows; scale bar 50 µm); (**h**) The expression of CD49f is suppressed in miR-200c mimic treated wounds compared to the control (arrows; scale bar 50 µm).

To further demonstrate the inhibitory effects of miR-200c on skin repair induced by injury, a human *ex vivo* skin wound healing model was used as described before^48^. Excisional wounds were treated with either miR-200c mimic or a scrambled control for 5 consecutive days (Fig. 3a). Histomorphological analysis of wound epithelium revealed that the area of the hyper-proliferative epithelium and the epithelial tongue length were significantly reduced in miR-200c treated biopsies compared to the controls (Fig. 3b,c). This was associated with significantly decreased proliferation in the regenerating epithelium, as was determined by quantitative analysis of Ki-67 positive cells (Fig. 3d,e). The wound epithelium treated with miR-200c mimic exhibited reduced expression of Keratin 16, Keratin 17 and CD49f (Integrin, alpha 6), markers of keratinocyte migration (Fig. 3f,g,h). The expression of Keratins 16 and 17 is normally induced in response to injury and stimulates the epithelialisation potential of keratinocytes^49, 50^. CD49f or laminin-binding integrin alpha6 also contributes to the successful re-epithelialisation by stimulating keratinocyte migration^51–53^. Therefore, this experiment confirms that aberrant levels of miR-200c may indeed compromise wound healing by suppressing the process of re-epithelialisation.

In conclusion, our study for the first time 1) reports differences in the expression of miRNAs in young and aged mouse skin wounds that suggest involvement of various miRNAs in age-associated impairment in wound healing; 2) provides evidence about contribution of miR-31 to the delay in wound healing in aged skin; 3) identifies miR-200c as an important player in successful re-epithelialisation during cutaneous wound healing that can exert positive and negative effects on keratinocyte differentiation and migration, respectively. Elevated levels of miR-200c in the skin could contribute to the age-associated delay in wound healing and compromised skin repair in chronic wounds.

## Material and Methods

### Animals and tissue collection

Animal studies were performed under protocols approved by UK Home Office Project License. A full-thickness 3 mm wound was introduced by punch biopsy onto back skin of 8-week-old and 2-year-old mice at the telogen stage of the hair cycle^54, 55^. Skin samples were collected on days 0, 3, 5, and 7 after wounding and were snap-frozen in liquid nitrogen^54, 55^.

### Human wound healing organ culture assay

Human skin was obtained with inform consent from elective plastic surgery cases (rhytidectomy) of healthy donors, and was used for experiments that were approved by the Ethics committee of the University of Bradford, and under the auspices of the Human Tissue Act UK (2006). To create a partial thickness cutaneous wound, two parallel incisions were made in the skin 1 mm apart extending to the mid-dermis and the central strip was sharply excised using dissecting scissors^48^. Punch biopsies with the linear partial thickness wound in the centre were excised and transferred to six-well plates containing Dulbecco’s modified Eagle medium (DMEM), supplemented with 10% foetal bovine serum, sodium pyruvate and antibiotics^48^. Acute wounds were topically treated at the time of wounding with 50 μM of miR-200c mimic and corresponding scrambled control (Dharmacon) dissolved in 30% pluronic F-127 gel (Sigma)^13^.

### Microarray and RT-qPCR analysis

Total RNA was isolated from homogenised tissue or cultured cells using the manufacturer’s protocol of TRI Reagent or Direct-zol RNA MiniPrep kit (Zymo Research). For eliminating genomic DNA, RNA samples were treated with DNase I (6 u/ul) (Zymo Research).

miRNA microarray profiling of young mouse wounds was performed using miRCURY LNA microRNA Array (7th Gen) (Exiqon, Vedbaek, Denmark). miRNA microarray profiling of aged mouse wounds was performed by LC Sciences (Houston, TX USA). Microarray data have been deposited to the Gene Expression Omnibus (GSE97034).

RT-qPCR for miR-31 and the members of miR-200 family was performed using corresponding TaqMan Real Time PCR Assays (Applied Biosystems) as described before^32, 56, 57^. For mRNA detection, total RNA was converted into complementary DNA using Reverse Transcription System (Promega, UK). RT-qPCR was performed on Applied Biosystems StepOne Plus system (Applied Biosystems) using Fast SYBR Green Master Mix (Applied Biosystems) and the corresponding primers (Table 2). Relative gene expression was calculated using the Genex software (Bio-Rad) based on the Ct (ΔΔCt) equitation method and normalised to U6 or *Gapdh*. Statistical analysis of RT-qPCR data was performed using Wilcoxon rank sum test.

**Table 2:** RT-qPCR primers.

Sequence definition	Sense primers	Antisense primers
Chloride intracellular channel protein 4 *(Clic4)*	CCGGAAGTGATGGTGAAAGC	AGGTTTCCTTTTCAGGTCAACG
Involucrin (*Ivl*)	CTCCTGTGAGTTTGTTTGGTC	CACACAGTCTTGAGAGGTCCC
Glyceraldehyde-3-phosphate dehydrogenase *(Gapdh)*	GTGTTCCTACCCCCAATGTG	AGGAGACAACCTGGTCCTCA
Keratin 1 *(Krt1)*	CTTCATCGACAAGGTGCGCT	GCGAGTCCACCTTCCTTCTG
Keratin 14 *(Krt14)*	CCACCTTTCATCTTCCCAATTCTC	GGTGGAGGTCACATCTCTGG
Hepatocyte growth factor receptor *(Met)*	CCAGCCCCTCTGCTTTCTTT	TCTCTCCACAGCCATCCTCG
Loricrin (*Lor*)	TCCCTGGTGCTTCAGGGTAAC	TCTTTCCACAACCCACAGGA
Ras-related C3 botulinum toxin substrate 1 *(Rac1)*	CGACACCACTGTCCCAATAC	GGTATTTGACAGCACCGATCT
Serum response factor *(Srf)*	GCAGTGATGTATGCCCCCAC	CAGCCATCTGGTGAAGCTGAA
Zinc finger E-box-binding homeobox 1 *(Zeb1)*	ACCCCTTCAAGAACCGCTTT	CAATTGGCCACCACTGCTAA

### Cell culture and transfections

Primary mouse epidermal keratinocytes (PMKs) were prepared from newborn mice and were grown in Eagle’s minimal essential medium EMEM (Lonza, UK) supplemented with 4% chelated foetal bovine serum as previously described^56, 58^. Primary human keratinocytes (PHKs) were isolated as previously described^59^ and grown in Keratinocyte Growth Medium 2 (PromoCell, Germany). HaCaT keratinocytes were grown in Dulbecco’s modified Eagle’s medium (Invitrogen) supplemented with heat-inactivated 10% foetal bovine serum and 1% penicillin-streptomycin at 37 °C, 5% CO_2_. To induce keratinocyte differentiation, cells were maintained in high-calcium medium (1.8 mM) for up to 72 hours. Transfections of the cells were performed using Lipofectamine RNAiMAX (ThermoFisher Scientific) using 100 nM miR-200c mimic (ThermoFisher Scientific), 200 nM inhibitor (Dharmacon) and corresponding negative controls according to the manufacturers’ protocol. All transfection and treatments were performed in triplicate.

### Transwell migration assay

Transwell assay was performed as previously described^54^. Twenty-four hours after cell seeding to the membrane in the upper chamber of the transwell insert, keratinocytes were transfected with miR-200c mimic or negative control RNA as described above. Keratinocytes were allowed to migrate over 48 hours through the insert membrane, after which the cells attached to the top surface of the membrane were removed with a cotton swab. Cells that had migrated to the bottom surface were fixed in 4% paraformaldehyde and counterstained with DAPI (Vector Laboratories, USA). The number of DAPI positive nuclei of migrated keratinocytes per microscopic field was counted. Statistical analysis was performed using Wilcoxon rank sum test.

### Scratch assay

Scratch assay was performed as previously described^54, 57^. A scratch was made in the monolayer of transfected keratinocytes using a P10 pipette tip. Mitomycin C (2 mg/ml; VWR) was included in the migration assay to block cell proliferation. The distance between the leading edges of the migrating keratinocytes was measured using ImageJ software (National Institutes of Health, Bethesda) and normalised to 0 hours. Statistical analysis was performed using unpaired Student’s t-test.

### Quantitative wound histomorphometry

*Ex vivo* wound samples (n = 5) were processed for hematoxylin and eosin (H&E) staining, and analysed using VisiCam (VWR International, UK) software. The epithelial tongue area (µm^2^) and length (µm) were measured and compared at day 5 post-wounding. To assess cell proliferation, the number of Ki-67+ and DAPI+ cells was counted along the basal layer of the wound epithelial tongue using ImageJ software (National Institutes of Health, Bethesda) and converted to a percentage, as previously described^55^. Statistical analysis was performed using unpaired Student’s t-test.

### Immunofluorescence

Formalin-fixed cryosections (10 µM-thickness) were incubated with primary antibodies against Ki-67 (Abcam; 1:100), Keratin 16 (Abcam; 1:250), Keratin 17 (Abcam; 1:100) and CD49f (BD Pharmingen; 1:100) overnight followed by application of the corresponding Alexa-546 or Alexa-555-labeled antibodies (Invitrogen, UK) for 45 min at 37 °C. Cell nuclei were counterstained with DAPI (Vector Labs, UK). Image analysis was performed using a fluorescent microscope in combination with DS-C1 digital camera and ACT-2U image analysis software (Nikon).

For BrdU analysis, the keratinocytes were seeded on collagen-coated sterile glass coverslips in a 6-well cell culture dish and transfected with miR-200c inhibitor or negative control RNA. 48 h after treatment, cells were treated with 10 µM BrdU (Sigma; 2 hours; 37 °C). Next, the cells were fixed with 4% paraformaldehyde (30 min, RT) followed by denaturation in 2 M HCl (30 min, 37 °C) and neutralisation in 0.1 M sodium borate. The cells were stained with FITC-conjugated Anti-BrdU (BD Biosciences; 30 min). Cell nuclei were counterstained with DAPI (Vector Labs, UK). Fluorescent microscopy images from 10 randomly selected fields per coverslip were taken, and the numbers of DAPI+ nuclei and FITC+ nuclei were counted using ImageJ software (NIH, Bethesda, MD USA). Statistical analysis was performed using Wilcoxon rank sum test.

### Flow cytometry

To assess proliferation rate, fluorescence-activated cell sorting (FACS) was performed with HaCaT cells stained with 20 ug/ml of 7-aminoactinomycin D (7-AAD) (VWR, UK; 15 min, RT) as previously described^32^. The percentage of cells at distinct phases of the cell cycle was analysed with a Beckman Coulter – CyAn2 ADP analyser (Beckman Coulter, UK).

## Electronic supplementary material

Supplementary information
