# Supplementary material for: Exploring a Role for Regulatory miRNAs In Wound Healing during Ageing:Involvement of miR-200c in wound repair
Source: Sci Rep. 2017 Jun 12;7:3257. doi: 10.1038/s41598-017-03331-6 (PMC5468284; doi:10.1038/s41598-017-03331-6)
Supplement: Supplementary file 1 — Supplementary information [file 41598_2017_3331_MOESM1_ESM.doc]

**Exploring a Role for Regulatory miRNAs In Wound Healing during Ageing: Involvement of miR-200c in wound repair**

Eerik Aunin, David Broadley, Mohammed I Ahmed, Andrei N Mardaryev and Natalia V Botchkareva

**Supplementary Table 1a.** Microarray analysis – dynamic changes of miRNA expression during distinct time points of wound healing in skin of 8 week-old mice

| Reporter name | day 0 | day 3 | day 5 |
| --- | --- | --- | --- |
| mmu-let-7a-2-3p | 249.8 | 273.6 | 356.6 |
| mmu-let-7a-5p | 2867.0 | 1626.0 | 1704.8 |
| mmu-let-7b-5p | 6119.6 | 2345.2 | 2311.2 |
| mmu-let-7c-5p | 6897.4 | 2808.4 | 2955.0 |
| mmu-let-7d-3p | 169.3 | 140.7 | 152.0 |
| mmu-let-7d-5p | 2519.0 | 1275.4 | 1439.0 |
| mmu-let-7e-5p | 1339.1 | 1156.2 | 1261.0 |
| mmu-let-7f-1-3p | 108.5 | 120.0 | 113.9 |
| mmu-let-7f-5p | 1053.1 | 603.3 | 718.9 |
| mmu-let-7g-5p | 3038.3 | 1750.1 | 1889.9 |
| mmu-let-7i-5p | 2546.2 | 1506.8 | 1816.2 |
| mmu-miR-100-5p | 586.0 | 199.7 | 229.4 |
| mmu-miR-101a-3p | 2378.1 | 964.5 | 983.0 |
| mmu-miR-101a-3p/mmu-miR-101c | 1398.4 | 527.4 | 558.7 |
| mmu-miR-101b-3p | 369.3 | 217.3 | 235.7 |
| mmu-miR-103-3p | 902.6 | 620.8 | 754.7 |
| mmu-miR-106a-5p | 533.8 | 591.3 | 456.6 |
| mmu-miR-106b-5p | 906.8 | 969.5 | 814.6 |
| mmu-miR-107-3p | 436.4 | 304.1 | 359.0 |
| mmu-miR-10a-5p | 1294.5 | 656.4 | 645.6 |
| mmu-miR-10b-5p | 1634.7 | 660.8 | 669.9 |
| mmu-miR-1186a | 92.7 | 104.6 | 106.8 |
| mmu-miR-1186b | 2123.7 | 1674.9 | 1562.0 |
| mmu-miR-1187 | 591.3 | 709.7 | 697.2 |
| mmu-miR-1192 | 1273.5 | 1459.5 | 1309.3 |
| mmu-miR-1196-5p | 1512.5 | 1904.5 | 2126.6 |
| mmu-miR-1224-3p | 367.1 | 377.9 | 447.2 |
| mmu-miR-1231-3p | 116.7 | 130.6 | 143.6 |
| mmu-miR-124-3p | 117.9 | 83.5 | 93.3 |
| mmu-miR-125a-5p | 2235.4 | 1158.1 | 1293.6 |
| mmu-miR-125b-2-3p | 112.4 | 68.9 | 72.9 |
| mmu-miR-125b-5p | 11861.4 | 4778.1 | 4602.7 |
| mmu-miR-126-3p | 2876.8 | 1623.1 | 3359.1 |
| mmu-miR-126-5p | 830.1 | 464.9 | 1034.6 |
| mmu-miR-1264-3p | 359.0 | 359.1 | 417.1 |
| mmu-miR-127-3p | 579.8 | 178.6 | 274.5 |
| mmu-miR-127-5p | 121.0 | 74.8 | 88.1 |
| mmu-miR-1298-5p | 125.7 | 110.5 | 84.7 |
| mmu-miR-130a-3p | 947.2 | 625.6 | 1039.8 |
| mmu-miR-133a-3p | 4358.4 | 1203.8 | 1658.4 |
| mmu-miR-133a-5p | 399.2 | 102.7 | 143.3 |
| mmu-miR-133b-3p | 6437.5 | 1699.9 | 2254.3 |
| mmu-miR-136-3p | 193.2 | 81.5 | 97.4 |
| mmu-miR-136-5p | 1032.7 | 454.3 | 769.9 |
| mmu-miR-138-2-3p | 269.4 | 227.5 | 287.6 |
| mmu-miR-138-5p | 211.3 | 241.1 | 156.5 |
| mmu-miR-139-5p | 464.2 | 595.2 | 704.0 |
| mmu-miR-140-3p | 586.5 | 520.9 | 458.5 |
| mmu-miR-140-5p | 287.2 | 237.5 | 206.7 |
| mmu-miR-141-3p | 1169.9 | 778.6 | 413.4 |
| mmu-miR-142-3p | 1851.3 | 9541.0 | 5694.8 |
| mmu-miR-142-5p | 406.6 | 1759.5 | 1017.0 |
| mmu-miR-143-3p | 3115.8 | 1384.1 | 1771.4 |
| mmu-miR-144-3p | 324.2 | 397.8 | 671.4 |
| mmu-miR-145a-5p | 959.6 | 409.3 | 519.1 |
| mmu-miR-146a-5p | 593.5 | 261.5 | 298.4 |
| mmu-miR-146b-5p | 729.7 | 710.9 | 606.9 |
| mmu-miR-147-3p | 91.9 | 181.7 | 134.4 |
| mmu-miR-148a-3p | 476.3 | 227.9 | 277.1 |
| mmu-miR-148b-3p | 157.0 | 161.0 | 157.9 |
| mmu-miR-149-5p | 115.1 | 100.4 | 94.8 |
| mmu-miR-150-5p | 555.6 | 335.1 | 393.8 |
| mmu-miR-151-5p | 167.5 | 104.7 | 128.1 |
| mmu-miR-152-3p | 882.5 | 361.7 | 514.5 |
| mmu-miR-154-5p | 234.9 | 99.9 | 122.5 |
| mmu-miR-155-3p | 232.6 | 186.9 | 213.5 |
| mmu-miR-15a-5p | 2548.6 | 2291.4 | 1829.7 |
| mmu-miR-15b-5p | 910.5 | 1419.8 | 1078.8 |
| mmu-miR-16-1-3p | 281.8 | 147.1 | 197.6 |
| mmu-miR-16-5p | 5718.0 | 5393.1 | 4292.1 |
| mmu-miR-17-3p | 158.6 | 182.4 | 171.3 |
| mmu-miR-17-5p | 558.1 | 577.1 | 419.3 |
| mmu-miR-181a-5p | 809.6 | 440.1 | 448.5 |
| mmu-miR-181b-5p | 185.9 | 113.8 | 121.6 |
| mmu-miR-181c-5p | 122.4 | 76.0 | 67.5 |
| mmu-miR-181d-5p | 342.8 | 201.9 | 203.4 |
| mmu-miR-182-5p | 388.2 | 148.2 | 106.5 |
| mmu-miR-183-3p | 278.9 | 270.0 | 273.7 |
| mmu-miR-183-5p | 219.8 | 140.4 | 107.5 |
| mmu-miR-1839-3p | 280.5 | 166.2 | 212.5 |
| mmu-miR-184-5p | 721.9 | 763.3 | 800.6 |
| mmu-miR-1843a-3p | 551.5 | 461.9 | 480.7 |
| mmu-miR-1843a-5p | 163.0 | 121.4 | 114.2 |
| mmu-miR-1843b-3p | 4779.6 | 4968.3 | 4818.4 |
| mmu-miR-1843b-5p | 190.4 | 138.5 | 132.1 |
| mmu-miR-185-3p | 1287.9 | 2601.8 | 1266.0 |
| mmu-miR-185-5p | 262.8 | 237.5 | 245.6 |
| mmu-miR-1892 | 103.6 | 161.5 | 129.6 |
| mmu-miR-1894-3p | 281.1 | 255.7 | 186.9 |
| mmu-miR-1895 | 246.5 | 259.1 | 256.1 |
| mmu-miR-1897-5p | 4652.9 | 4693.7 | 5063.6 |
| mmu-miR-1899 | 153.6 | 191.5 | 122.3 |
| mmu-miR-1900 | 1196.0 | 1069.8 | 1094.1 |
| mmu-miR-1903 | 161.1 | 201.9 | 210.7 |
| mmu-miR-190a-5p | 153.2 | 106.7 | 167.8 |
| mmu-miR-191-5p | 676.3 | 568.9 | 526.8 |
| mmu-miR-1929-5p | 337.1 | 359.2 | 313.2 |
| mmu-miR-1934-5p | 423.6 | 365.4 | 536.1 |
| mmu-miR-1935 | 442.5 | 629.0 | 486.2 |
| mmu-miR-193a-3p | 536.4 | 479.6 | 667.5 |
| mmu-miR-193b-3p | 112.2 | 62.7 | 78.1 |
| mmu-miR-1941-3p | 156.0 | 164.8 | 160.6 |
| mmu-miR-1943-3p | 278.0 | 368.1 | 329.3 |
| mmu-miR-1946b | 119.6 | 171.5 | 137.2 |
| mmu-miR-1947-3p | 2625.6 | 2928.8 | 2737.4 |
| mmu-miR-1949 | 192.1 | 427.5 | 333.1 |
| mmu-miR-1952 | 1398.1 | 1210.2 | 1487.7 |
| mmu-miR-1954 | 370.5 | 321.7 | 378.7 |
| mmu-miR-1957a | 341.7 | 536.6 | 431.3 |
| mmu-miR-1958 | 248.1 | 316.1 | 327.4 |
| mmu-miR-195a-3p | 123.2 | 118.7 | 126.7 |
| mmu-miR-195a-5p | 3635.8 | 1303.6 | 1293.8 |
| mmu-miR-1960 | 103.3 | 120.4 | 115.3 |
| mmu-miR-1961 | 193.2 | 131.1 | 128.9 |
| mmu-miR-196a-2-3p | 134.1 | 124.0 | 169.0 |
| mmu-miR-196a-5p | 184.7 | 153.0 | 120.1 |
| mmu-miR-196b-5p | 109.4 | 92.2 | 79.5 |
| mmu-miR-1971 | 966.1 | 1776.2 | 953.9 |
| mmu-miR-1981-3p | 1028.1 | 542.0 | 818.3 |
| mmu-miR-1983 | 2440.2 | 2809.6 | 2934.1 |
| mmu-miR-199a-3p/mmu-miR-199b-3p | 3488.5 | 1993.6 | 2718.7 |
| mmu-miR-199a-5p | 3865.2 | 2116.1 | 3020.3 |
| mmu-miR-199b-5p | 2768.7 | 1316.0 | 1795.6 |
| mmu-miR-19a-3p | 420.7 | 626.3 | 540.7 |
| mmu-miR-19b-3p | 1000.2 | 973.2 | 845.1 |
| mmu-miR-1a-1-5p | 221.5 | 81.1 | 98.9 |
| mmu-miR-1a-2-5p | 210.0 | 75.9 | 93.7 |
| mmu-miR-1a-3p | 12351.8 | 3400.9 | 4340.5 |
| mmu-miR-200a-3p | 1260.5 | 520.5 | 236.8 |
| mmu-miR-200b-3p | 1312.0 | 551.8 | 324.9 |
| mmu-miR-200c-3p | 893.9 | 605.2 | 299.5 |
| mmu-miR-203-3p | 9109.0 | 4087.1 | 1998.2 |
| mmu-miR-203-5p | 234.9 | 150.7 | 97.6 |
| mmu-miR-204-3p | 301.6 | 402.3 | 430.3 |
| mmu-miR-204-5p | 117.1 | 75.0 | 71.7 |
| mmu-miR-205-5p | 15501.1 | 8513.0 | 4138.8 |
| mmu-miR-206-3p | 1144.4 | 466.5 | 1219.8 |
| mmu-miR-207 | 998.4 | 781.9 | 994.8 |
| mmu-miR-20a-5p | 840.3 | 969.6 | 741.7 |
| mmu-miR-20b-5p | 353.7 | 375.1 | 281.5 |
| mmu-miR-210-3p | 344.0 | 413.6 | 363.5 |
| mmu-miR-2137 | 2128.1 | 7569.0 | 4595.4 |
| mmu-miR-2139 | 113.2 | 109.1 | 116.5 |
| mmu-miR-214-3p | 1079.1 | 1040.7 | 1503.6 |
| mmu-miR-214-5p | 128.7 | 127.5 | 174.4 |
| mmu-miR-218-5p | 153.2 | 113.5 | 129.8 |
| mmu-miR-2183 | 351.7 | 307.1 | 349.5 |
| mmu-miR-21a-3p | 478.3 | 771.7 | 659.5 |
| mmu-miR-21a-5p | 2448.3 | 6421.8 | 6902.5 |
| mmu-miR-22-3p | 6106.8 | 3986.4 | 4758.0 |
| mmu-miR-22-5p | 344.8 | 229.2 | 259.9 |
| mmu-miR-221-3p | 400.7 | 378.2 | 364.4 |
| mmu-miR-221-5p | 656.6 | 257.8 | 387.3 |
| mmu-miR-222-3p | 399.7 | 337.3 | 333.0 |
| mmu-miR-223-3p | 1037.8 | 10333.7 | 5199.3 |
| mmu-miR-224-3p | 462.9 | 236.7 | 311.7 |
| mmu-miR-23a-3p | 9875.7 | 4847.5 | 4024.2 |
| mmu-miR-23b-3p | 8665.4 | 4386.8 | 3503.6 |
| mmu-miR-24-1-5p | 272.5 | 181.9 | 164.9 |
| mmu-miR-24-2-5p | 458.4 | 297.5 | 253.1 |
| mmu-miR-24-3p | 8211.4 | 4181.4 | 3461.5 |
| mmu-miR-25-3p | 309.7 | 308.6 | 270.6 |
| mmu-miR-25-5p | 159.0 | 184.7 | 222.5 |
| mmu-miR-26a-5p | 1028.0 | 468.6 | 462.6 |
| mmu-miR-26b-5p | 3059.7 | 1930.5 | 1654.5 |
| mmu-miR-27a-3p | 3788.7 | 2385.7 | 1943.9 |
| mmu-miR-27b-3p | 2589.3 | 1427.2 | 1276.5 |
| mmu-miR-2861 | 167.1 | 227.5 | 243.9 |
| mmu-miR-28a-5p/mmu-miR-28c | 224.0 | 141.1 | 171.1 |
| mmu-miR-290-3p | 639.5 | 723.4 | 698.5 |
| mmu-miR-290-5p | 489.8 | 874.3 | 811.8 |
| mmu-miR-291a-5p | 192.9 | 269.1 | 259.1 |
| mmu-miR-291b-5p | 138.3 | 189.6 | 176.8 |
| mmu-miR-294-5p | 570.2 | 863.1 | 746.0 |
| mmu-miR-297a-3p/mmu-miR-297b-3p/mmu-miR-297c-3p | 555.8 | 697.2 | 598.6 |
| mmu-miR-297a-5p | 146.5 | 197.9 | 185.6 |
| mmu-miR-297c-5p | 226.0 | 304.7 | 306.8 |
| mmu-miR-299a-5p | 463.2 | 347.5 | 439.7 |
| mmu-miR-299a-5p/mmu-miR-299b-5p | 138.6 | 78.2 | 93.4 |
| mmu-miR-29a-3p | 3973.6 | 2339.7 | 2363.3 |
| mmu-miR-29a-5p | 157.5 | 111.4 | 113.6 |
| mmu-miR-29b-1-5p | 88.2 | 158.3 | 112.8 |
| mmu-miR-29b-3p | 1934.7 | 1454.4 | 1583.7 |
| mmu-miR-29c-3p | 1113.0 | 552.9 | 632.8 |
| mmu-miR-300-3p | 161.9 | 113.0 | 145.2 |
| mmu-miR-300-5p | 1730.3 | 2156.1 | 1837.3 |
| mmu-miR-301a-3p | 196.3 | 218.0 | 203.9 |
| mmu-miR-302a-3p | 1793.2 | 1148.6 | 1602.6 |
| mmu-miR-3060-3p | 178.4 | 214.1 | 204.9 |
| mmu-miR-3064-5p | 102.7 | 113.3 | 104.8 |
| mmu-miR-3065-3p | 62.4 | 185.2 | 141.1 |
| mmu-miR-3068-3p | 1725.4 | 2220.5 | 2480.7 |
| mmu-miR-3068-5p | 143.6 | 102.6 | 110.9 |
| mmu-miR-3069-3p | 155.1 | 177.6 | 186.7 |
| mmu-miR-3072-3p | 104.9 | 127.8 | 111.8 |
| mmu-miR-3076-3p | 178.2 | 125.5 | 131.9 |
| mmu-miR-3077-3p | 185.0 | 173.3 | 192.3 |
| mmu-miR-3078-3p | 105.5 | 110.9 | 92.8 |
| mmu-miR-3082-5p | 2769.6 | 2993.5 | 2765.6 |
| mmu-miR-3084-3p | 1152.3 | 1170.8 | 1086.2 |
| mmu-miR-3084-5p | 382.6 | 415.3 | 430.4 |
| mmu-miR-3090-5p | 140.2 | 180.1 | 187.9 |
| mmu-miR-3095-3p | 256.2 | 440.8 | 369.3 |
| mmu-miR-3096a-3p | 465.3 | 854.1 | 753.3 |
| mmu-miR-3096a-3p/mmu-miR-3096b-3p | 280.3 | 487.3 | 448.3 |
| mmu-miR-3096a-5p | 86.3 | 121.3 | 113.5 |
| mmu-miR-3096b-5p | 80.0 | 123.2 | 112.4 |
| mmu-miR-3097-5p | 146.9 | 202.4 | 188.9 |
| mmu-miR-3098-3p | 557.1 | 542.0 | 643.0 |
| mmu-miR-3099-3p | 104.2 | 122.2 | 119.2 |
| mmu-miR-3099-5p | 134.7 | 166.6 | 157.1 |
| mmu-miR-30a-5p | 905.0 | 399.8 | 490.5 |
| mmu-miR-30b-3p | 237.2 | 284.7 | 297.5 |
| mmu-miR-30b-5p | 2462.0 | 1199.8 | 1271.6 |
| mmu-miR-30c-1-3p | 87.8 | 117.0 | 91.5 |
| mmu-miR-30c-5p | 2838.6 | 1265.6 | 1473.4 |
| mmu-miR-30d-5p | 882.8 | 441.2 | 481.3 |
| mmu-miR-30e-3p | 306.9 | 151.3 | 169.7 |
| mmu-miR-30e-5p | 1451.3 | 795.9 | 927.5 |
| mmu-miR-31-5p | 95.7 | 175.6 | 152.3 |
| mmu-miR-3100-3p | 1497.1 | 1942.2 | 1544.7 |
| mmu-miR-3102-5p | 106.6 | 103.5 | 124.9 |
| mmu-miR-3103-3p | 1258.0 | 1063.5 | 1446.4 |
| mmu-miR-3103-5p | 175.2 | 201.8 | 227.6 |
| mmu-miR-3107-5p/mmu-miR-486-5p | 157.0 | 112.1 | 136.4 |
| mmu-miR-32-3p | 1991.1 | 2085.6 | 1961.5 |
| mmu-miR-32-5p | 169.5 | 197.6 | 205.0 |
| mmu-miR-320-3p | 430.5 | 193.7 | 190.4 |
| mmu-miR-320-5p | 139.1 | 153.4 | 161.5 |
| mmu-miR-322-5p | 309.7 | 323.1 | 533.8 |
| mmu-miR-325-3p | 559.8 | 573.0 | 601.9 |
| mmu-miR-325-5p | 181.3 | 96.5 | 104.6 |
| mmu-miR-328-3p | 129.3 | 114.4 | 122.6 |
| mmu-miR-329-3p | 346.0 | 221.1 | 282.8 |
| mmu-miR-329-5p | 130.5 | 95.4 | 110.9 |
| mmu-miR-33-5p | 311.4 | 326.1 | 242.2 |
| mmu-miR-330-3p | 87.5 | 126.0 | 112.3 |
| mmu-miR-331-3p | 138.4 | 105.5 | 121.3 |
| mmu-miR-335-3p | 2035.9 | 1894.1 | 2462.0 |
| mmu-miR-335-5p | 128.5 | 97.5 | 246.6 |
| mmu-miR-337-3p | 234.7 | 168.4 | 215.4 |
| mmu-miR-338-3p | 434.3 | 293.3 | 222.8 |
| mmu-miR-338-5p | 379.9 | 153.7 | 165.5 |
| mmu-miR-339-5p | 171.1 | 176.5 | 180.4 |
| mmu-miR-340-5p | 442.4 | 600.0 | 553.2 |
| mmu-miR-341-3p | 139.2 | 161.0 | 164.5 |
| mmu-miR-342-3p | 141.8 | 111.4 | 155.4 |
| mmu-miR-342-5p | 161.9 | 84.4 | 96.2 |
| mmu-miR-344h-3p | 338.8 | 379.6 | 414.7 |
| mmu-miR-345-5p | 136.5 | 142.6 | 126.9 |
| mmu-miR-346-3p | 557.5 | 928.8 | 510.4 |
| mmu-miR-3470a | 140.0 | 181.2 | 181.0 |
| mmu-miR-3470b | 299.8 | 420.5 | 454.6 |
| mmu-miR-3473a | 1706.3 | 2073.6 | 1712.1 |
| mmu-miR-3473b | 9382.1 | 12431.6 | 12597.0 |
| mmu-miR-3474 | 2061.2 | 1868.9 | 2764.9 |
| mmu-miR-34a-5p | 364.5 | 269.1 | 375.0 |
| mmu-miR-34b-3p | 310.0 | 378.3 | 413.3 |
| mmu-miR-34b-5p | 91.7 | 121.9 | 184.6 |
| mmu-miR-34c-3p | 104.4 | 115.5 | 113.7 |
| mmu-miR-34c-5p | 127.4 | 169.1 | 211.4 |
| mmu-miR-350-3p | 193.0 | 186.7 | 213.7 |
| mmu-miR-351-5p | 671.7 | 876.1 | 838.4 |
| mmu-miR-3544-3p | 203.7 | 117.2 | 178.9 |
| mmu-miR-3572-3p | 689.0 | 679.9 | 914.6 |
| mmu-miR-361-3p | 97.0 | 111.9 | 95.9 |
| mmu-miR-361-5p | 229.8 | 253.5 | 226.1 |
| mmu-miR-362-3p | 175.0 | 176.0 | 184.4 |
| mmu-miR-365-3p | 697.7 | 370.9 | 585.1 |
| mmu-miR-374b-5p | 178.1 | 138.4 | 142.6 |
| mmu-miR-374b-5p/mmu-miR-374c-5p | 134.4 | 108.6 | 111.9 |
| mmu-miR-376a-3p | 170.3 | 103.7 | 198.8 |
| mmu-miR-376b-3p | 120.5 | 85.0 | 149.7 |
| mmu-miR-376b-5p | 145.4 | 68.7 | 74.6 |
| mmu-miR-376c-3p | 120.2 | 79.5 | 102.4 |
| mmu-miR-377-3p | 214.5 | 103.9 | 179.0 |
| mmu-miR-378a-3p | 1661.7 | 734.1 | 1198.6 |
| mmu-miR-378b | 1623.3 | 693.7 | 1144.3 |
| mmu-miR-379-5p | 639.5 | 220.7 | 398.5 |
| mmu-miR-381-5p | 128.8 | 98.9 | 97.2 |
| mmu-miR-382-5p | 149.5 | 104.8 | 126.9 |
| mmu-miR-3960 | 614.1 | 786.8 | 729.4 |
| mmu-miR-3961 | 1619.4 | 1557.7 | 1453.7 |
| mmu-miR-3962 | 1776.3 | 1008.9 | 1051.4 |
| mmu-miR-3963 | 51301.1 | 46377.3 | 43228.9 |
| mmu-miR-3964 | 110.0 | 203.6 | 155.6 |
| mmu-miR-3968 | 818.9 | 946.1 | 1192.4 |
| mmu-miR-3970 | 838.8 | 531.0 | 679.0 |
| mmu-miR-411-3p | 132.5 | 82.1 | 150.8 |
| mmu-miR-411-5p | 289.9 | 106.3 | 138.5 |
| mmu-miR-412-3p | 356.4 | 111.3 | 151.2 |
| mmu-miR-423-3p | 118.4 | 155.7 | 137.4 |
| mmu-miR-423-5p | 339.5 | 425.7 | 403.4 |
| mmu-miR-425-5p | 137.3 | 134.8 | 138.3 |
| mmu-miR-429-3p | 525.3 | 214.3 | 122.1 |
| mmu-miR-433-5p | 758.8 | 606.7 | 717.4 |
| mmu-miR-434-3p | 341.5 | 132.7 | 196.8 |
| mmu-miR-434-5p | 243.2 | 93.1 | 132.1 |
| mmu-miR-450a-1-3p | 124.7 | 135.3 | 144.8 |
| mmu-miR-451a | 4040.9 | 3183.0 | 5040.4 |
| mmu-miR-455-3p | 98.3 | 86.3 | 208.2 |
| mmu-miR-465b-5p | 1222.6 | 879.9 | 830.1 |
| mmu-miR-465c-5p | 102.0 | 123.5 | 109.9 |
| mmu-miR-466a-3p/mmu-miR-466b-3p/mmu-miR-466c-3p/mmu-miR-466e-3p/mmu-miR-466p-3p | 388.5 | 437.4 | 382.1 |
| mmu-miR-466a-3p/mmu-miR-466e-3p | 102.8 | 123.1 | 106.3 |
| mmu-miR-466a-5p | 597.7 | 690.7 | 691.1 |
| mmu-miR-466a-5p/mmu-miR-466p-5p | 1178.7 | 1220.2 | 1191.6 |
| mmu-miR-466b-5p/mmu-miR-466o-5p | 658.7 | 878.5 | 861.8 |
| mmu-miR-466c-5p | 932.1 | 993.8 | 989.2 |
| mmu-miR-466d-3p | 267.8 | 308.4 | 284.3 |
| mmu-miR-466d-5p | 1234.8 | 1471.9 | 1395.2 |
| mmu-miR-466e-5p | 697.0 | 721.5 | 763.8 |
| mmu-miR-466f | 1893.5 | 2116.0 | 1969.0 |
| mmu-miR-466f-3p | 1465.7 | 1780.0 | 1425.8 |
| mmu-miR-466f-5p | 426.1 | 558.1 | 577.1 |
| mmu-miR-466i-3p | 499.6 | 607.4 | 508.7 |
| mmu-miR-466i-5p | 3601.9 | 3582.6 | 3434.1 |
| mmu-miR-466j | 350.8 | 510.3 | 481.8 |
| mmu-miR-466m-3p | 112.3 | 121.9 | 102.9 |
| mmu-miR-466m-5p/mmu-miR-669m-5p | 381.8 | 484.2 | 469.0 |
| mmu-miR-466n-3p | 143.0 | 166.4 | 161.6 |
| mmu-miR-466n-5p | 537.0 | 707.8 | 667.6 |
| mmu-miR-466q | 982.7 | 1204.4 | 1044.4 |
| mmu-miR-467a-3p | 709.2 | 945.1 | 775.5 |
| mmu-miR-467b-3p | 224.8 | 275.6 | 234.1 |
| mmu-miR-467c-3p | 332.0 | 444.6 | 377.0 |
| mmu-miR-467d-3p | 195.8 | 227.3 | 196.0 |
| mmu-miR-467e-3p | 1661.8 | 2298.1 | 1802.7 |
| mmu-miR-467e-5p | 236.7 | 280.5 | 281.6 |
| mmu-miR-467f | 857.6 | 1220.7 | 1006.6 |
| mmu-miR-467g | 1012.1 | 1343.1 | 1079.5 |
| mmu-miR-467h | 328.6 | 308.5 | 356.7 |
| mmu-miR-468-3p | 412.1 | 539.1 | 490.6 |
| mmu-miR-470-5p | 151.7 | 149.1 | 148.0 |
| mmu-miR-485-3p | 381.2 | 311.7 | 308.3 |
| mmu-miR-487b-3p | 210.7 | 205.2 | 248.2 |
| mmu-miR-487b-5p | 109.6 | 117.3 | 116.9 |
| mmu-miR-490-3p | 861.9 | 551.0 | 826.7 |
| mmu-miR-491-3p | 20434.3 | 15326.3 | 17451.9 |
| mmu-miR-493-5p | 299.9 | 365.6 | 405.7 |
| mmu-miR-495-5p | 249.0 | 247.9 | 281.1 |
| mmu-miR-497-5p | 646.8 | 239.5 | 263.3 |
| mmu-miR-500-3p | 238.4 | 191.2 | 192.7 |
| mmu-miR-503-3p | 670.0 | 749.1 | 845.0 |
| mmu-miR-503-5p | 260.0 | 341.3 | 442.8 |
| mmu-miR-505-5p | 181.4 | 201.7 | 192.7 |
| mmu-miR-5097 | 1244.1 | 2310.5 | 2662.2 |
| mmu-miR-5098 | 186.0 | 164.8 | 192.6 |
| mmu-miR-5099 | 837.1 | 1620.2 | 1276.8 |
| mmu-miR-5100 | 39857.4 | 28952.3 | 40816.8 |
| mmu-miR-5105 | 257.5 | 260.5 | 271.1 |
| mmu-miR-5107-5p | 179.8 | 223.1 | 202.2 |
| mmu-miR-5108 | 123.2 | 128.9 | 143.9 |
| mmu-miR-5109 | 7226.3 | 6849.9 | 5999.0 |
| mmu-miR-511-3p | 173.7 | 239.6 | 207.0 |
| mmu-miR-5113 | 2857.1 | 3519.4 | 3320.2 |
| mmu-miR-5114 | 145.6 | 146.4 | 175.0 |
| mmu-miR-5115 | 149.1 | 242.8 | 218.0 |
| mmu-miR-5116 | 1425.2 | 3482.9 | 2052.5 |
| mmu-miR-5117-3p | 1896.6 | 2489.4 | 2335.2 |
| mmu-miR-5117-5p | 87.6 | 107.3 | 108.4 |
| mmu-miR-5119 | 210.2 | 195.1 | 236.1 |
| mmu-miR-5120 | 184.3 | 211.1 | 236.6 |
| mmu-miR-5121 | 176.7 | 160.9 | 152.8 |
| mmu-miR-5125 | 555.8 | 477.8 | 585.3 |
| mmu-miR-5126 | 91.2 | 135.0 | 145.1 |
| mmu-miR-5128 | 163.2 | 220.4 | 202.2 |
| mmu-miR-5129-5p | 110.8 | 116.4 | 140.0 |
| mmu-miR-5132-5p | 84.2 | 123.3 | 89.1 |
| mmu-miR-532-5p | 104.3 | 101.3 | 106.9 |
| mmu-miR-541-3p | 192.2 | 188.1 | 282.2 |
| mmu-miR-541-5p | 284.3 | 143.4 | 250.0 |
| mmu-miR-542-5p | 121.2 | 150.0 | 130.5 |
| mmu-miR-544-5p | 249.2 | 262.3 | 219.3 |
| mmu-miR-551b-5p | 385.8 | 295.0 | 475.5 |
| mmu-miR-5616-3p | 160.7 | 133.5 | 157.0 |
| mmu-miR-5616-5p | 230.3 | 298.6 | 297.6 |
| mmu-miR-5621-3p | 203.0 | 106.8 | 117.5 |
| mmu-miR-5621-5p | 178.7 | 168.9 | 194.5 |
| mmu-miR-5622-5p | 185.8 | 214.6 | 187.4 |
| mmu-miR-5624-3p | 205.5 | 253.3 | 234.5 |
| mmu-miR-5624-5p | 509.8 | 587.8 | 719.6 |
| mmu-miR-5626-5p | 137.6 | 146.8 | 136.2 |
| mmu-miR-574-3p | 199.5 | 164.1 | 151.5 |
| mmu-miR-574-5p | 925.3 | 1044.2 | 1007.7 |
| mmu-miR-592-3p | 238.0 | 194.8 | 270.6 |
| mmu-miR-615-3p | 972.0 | 307.1 | 340.0 |
| mmu-miR-652-3p | 217.7 | 222.0 | 176.9 |
| mmu-miR-653-3p | 113.2 | 119.6 | 112.6 |
| mmu-miR-664-3p | 174.9 | 206.3 | 177.9 |
| mmu-miR-665-3p | 617.1 | 1095.2 | 1616.9 |
| mmu-miR-665-5p | 905.8 | 905.2 | 1024.4 |
| mmu-miR-666-5p | 97.0 | 135.4 | 130.1 |
| mmu-miR-667-3p | 993.5 | 884.1 | 990.6 |
| mmu-miR-668-3p | 168.5 | 257.9 | 315.3 |
| mmu-miR-669a-3-3p | 1362.1 | 1735.5 | 1397.0 |
| mmu-miR-669a-3p/mmu-miR-669o-3p | 824.0 | 1052.1 | 934.0 |
| mmu-miR-669a-5p/mmu-miR-669p-5p | 250.5 | 322.8 | 331.9 |
| mmu-miR-669b-3p | 856.9 | 1042.6 | 909.3 |
| mmu-miR-669c-3p | 1436.9 | 1791.3 | 1439.5 |
| mmu-miR-669c-5p | 2442.2 | 2570.7 | 2533.5 |
| mmu-miR-669d-2-3p | 158.3 | 180.5 | 164.1 |
| mmu-miR-669d-2-3p/mmu-miR-669d-3p | 848.6 | 1044.8 | 964.7 |
| mmu-miR-669d-5p | 1356.0 | 1417.2 | 1323.7 |
| mmu-miR-669e-3p | 278.3 | 295.8 | 263.9 |
| mmu-miR-669e-5p | 635.9 | 735.3 | 752.0 |
| mmu-miR-669f-3p | 1133.3 | 1470.5 | 1185.5 |
| mmu-miR-669f-5p | 1132.5 | 1315.4 | 1301.7 |
| mmu-miR-669h-3p | 103.0 | 111.6 | 93.9 |
| mmu-miR-669i | 131.1 | 147.6 | 118.7 |
| mmu-miR-669k-5p | 1007.4 | 1434.8 | 1199.8 |
| mmu-miR-669l-3p | 876.7 | 1067.2 | 974.7 |
| mmu-miR-669l-5p | 768.8 | 952.6 | 887.9 |
| mmu-miR-669m-3p | 502.6 | 565.2 | 543.2 |
| mmu-miR-669n | 1570.8 | 1870.6 | 1904.1 |
| mmu-miR-669o-5p | 932.6 | 1156.6 | 1115.1 |
| mmu-miR-669p-3p | 1731.7 | 2360.7 | 1825.9 |
| mmu-miR-673-3p | 109.3 | 137.8 | 118.1 |
| mmu-miR-674-5p | 250.4 | 253.3 | 282.6 |
| mmu-miR-675-5p | 486.5 | 545.1 | 387.4 |
| mmu-miR-676-3p | 124.2 | 78.3 | 77.7 |
| mmu-miR-677-3p | 1764.6 | 1242.2 | 2315.9 |
| mmu-miR-677-5p | 132.7 | 143.5 | 140.2 |
| mmu-miR-678 | 143.0 | 178.4 | 174.0 |
| mmu-miR-688 | 95.9 | 136.6 | 164.0 |
| mmu-miR-690 | 12878.4 | 15955.5 | 15608.7 |
| mmu-miR-691 | 2538.9 | 2981.3 | 3015.6 |
| mmu-miR-693-5p | 253.0 | 270.2 | 288.2 |
| mmu-miR-695 | 113.6 | 131.0 | 143.9 |
| mmu-miR-697 | 3223.1 | 1306.8 | 1743.7 |
| mmu-miR-705 | 298.0 | 344.3 | 295.9 |
| mmu-miR-706 | 2641.1 | 3599.9 | 3533.5 |
| mmu-miR-708-5p | 229.0 | 147.9 | 179.9 |
| mmu-miR-709 | 10352.3 | 13378.2 | 13361.8 |
| mmu-miR-710 | 326.0 | 631.1 | 499.0 |
| mmu-miR-711 | 326.9 | 480.8 | 401.2 |
| mmu-miR-713 | 113.7 | 118.2 | 126.1 |
| mmu-miR-714 | 190.4 | 390.8 | 373.7 |
| mmu-miR-721 | 133.4 | 156.9 | 154.4 |
| mmu-miR-744-5p | 546.6 | 783.5 | 619.5 |
| mmu-miR-758-5p | 224.6 | 293.8 | 284.0 |
| mmu-miR-762 | 201.2 | 308.4 | 369.9 |
| mmu-miR-763 | 344.5 | 294.4 | 371.5 |
| mmu-miR-767 | 179.7 | 285.8 | 326.3 |
| mmu-miR-7a-2-3p | 165.5 | 135.9 | 111.5 |
| mmu-miR-7a-5p | 167.4 | 256.9 | 245.5 |
| mmu-miR-7b-3p | 126.5 | 123.0 | 123.4 |
| mmu-miR-872-3p | 655.7 | 784.3 | 842.6 |
| mmu-miR-875-3p | 1565.7 | 1526.2 | 1589.0 |
| mmu-miR-877-5p | 162.2 | 178.4 | 166.4 |
| mmu-miR-881-3p | 143.0 | 161.8 | 150.6 |
| mmu-miR-881-5p | 333.0 | 444.0 | 404.6 |
| mmu-miR-882 | 791.4 | 1207.3 | 1047.7 |
| mmu-miR-883a-5p | 1070.3 | 1453.6 | 1338.8 |
| mmu-miR-883b-5p | 304.9 | 256.7 | 238.2 |
| mmu-miR-92a-2-5p | 96.6 | 107.8 | 118.7 |
| mmu-miR-92a-3p | 427.8 | 422.6 | 329.3 |
| mmu-miR-92b-3p | 115.8 | 106.2 | 108.5 |
| mmu-miR-93-5p | 493.1 | 568.5 | 445.2 |
| mmu-miR-96-5p | 285.9 | 161.8 | 98.0 |
| mmu-miR-98-5p | 1503.3 | 700.2 | 776.5 |
| mmu-miR-99a-5p | 1735.0 | 607.8 | 650.8 |
| mmu-miR-99b-3p | 129.3 | 149.6 | 136.4 |
| mmu-miR-99b-5p | 439.6 | 258.4 | 325.6 |

**Supplementary Table 1b.** Microarray analysis – dynamic changes of miRNA expression during distinct time points of wound healing in skin of 2 year-old mice

| Reporter Name | day 0 | day 3 | day 5 | day 7 |
| --- | --- | --- | --- | --- |
| mmu-let-7a-1-3p | 47 | 41 | 27 | 63 |
| mmu-let-7a-2-3p | 6 | 4 | 4 | 8 |
| mmu-let-7a-5p | 7,950 | 4,287 | 6,030 | 7,021 |
| mmu-let-7b-3p | 25 | 27 | 27 | 12 |
| mmu-let-7b-5p | 4,012 | 1,572 | 2,694 | 3,196 |
| mmu-let-7c-1-3p | 9 | 19 | 0 | 12 |
| mmu-let-7c-5p | 6,997 | 3,276 | 4,442 | 5,113 |
| mmu-let-7d-3p | 135 | 83 | 113 | 87 |
| mmu-let-7d-5p | 5,547 | 3,817 | 5,278 | 6,347 |
| mmu-let-7e-3p | 12 | 5 | 9 | 6 |
| mmu-let-7e-5p | 780 | 336 | 720 | 1,284 |
| mmu-let-7f-1-3p | 8 | 7 | 1 | 12 |
| mmu-let-7f-2-3p | 5 | 2 | 0 | 7 |
| mmu-let-7f-5p | 6,991 | 4,372 | 5,605 | 7,517 |
| mmu-let-7g-3p | 14 | 11 | 10 | 8 |
| mmu-let-7g-5p | 2,189 | 1,854 | 2,184 | 2,207 |
| mmu-let-7i-3p | 2 | 4 | 0 | 4 |
| mmu-let-7i-5p | 1,944 | 2,150 | 2,714 | 2,448 |
| mmu-let-7j | 1,419 | 1,367 | 2,123 | 2,029 |
| mmu-let-7k | 1,852 | 686 | 1,037 | 1,379 |
| mmu-miR-1a-1-5p | 38 | 7 | 0 | 20 |
| mmu-miR-1a-2-5p | 1 | 0 | 0 | 0 |
| mmu-miR-1a-3p | 7,348 | 2,209 | 1,295 | 3,845 |
| mmu-miR-1b-3p | 1 | 0 | 0 | 0 |
| mmu-miR-1b-5p | 10 | 1 | 0 | 0 |
| mmu-miR-7a-1-3p | 0 | 24 | 3 | 30 |
| mmu-miR-7a-2-3p | 0 | 10 | 0 | 1 |
| mmu-miR-7a-5p | 4 | 90 | 102 | 94 |
| mmu-miR-7b-3p | 13 | 123 | 44 | 69 |
| mmu-miR-7b-5p | 0 | 71 | 0 | 0 |
| mmu-miR-9-3p | 1 | 0 | 0 | 0 |
| mmu-miR-9-5p | 1 | 0 | 0 | 4 |
| mmu-miR-10a-3p | 2 | 12 | 0 | 20 |
| mmu-miR-10a-5p | 843 | 667 | 327 | 705 |
| mmu-miR-10b-3p | 0 | 4 | 0 | 13 |
| mmu-miR-10b-5p | 1,862 | 885 | 816 | 1,799 |
| mmu-miR-15a-3p | 23 | 15 | 16 | 19 |
| mmu-miR-15a-5p | 455 | 607 | 498 | 1,053 |
| mmu-miR-15b-3p | 0 | 32 | 3 | 39 |
| mmu-miR-15b-5p | 1,224 | 2,475 | 1,584 | 1,683 |
| mmu-miR-16-1-3p | 0 | 20 | 8 | 14 |
| mmu-miR-16-2-3p | 0 | 18 | 0 | 19 |
| mmu-miR-16-5p | 5,537 | 7,727 | 7,132 | 6,983 |
| mmu-miR-17-3p | 34 | 53 | 51 | 56 |
| mmu-miR-17-5p | 1,121 | 1,250 | 1,172 | 1,374 |
| mmu-miR-18a-3p | 17 | 18 | 30 | 14 |
| mmu-miR-18a-5p | 56 | 65 | 69 | 50 |
| mmu-miR-18b-3p | 3 | 0 | 1 | 0 |
| mmu-miR-18b-5p | 7 | 0 | 2 | 3 |
| mmu-miR-19a-3p | 0 | 4 | 0 | 13 |
| mmu-miR-19a-5p | 0 | 0 | 0 | 1 |
| mmu-miR-19b-1-5p | 3 | 9 | 3 | 11 |
| mmu-miR-19b-2-5p | 0 | 2 | 0 | 1 |
| mmu-miR-19b-3p | 242 | 235 | 127 | 454 |
| mmu-miR-20a-3p | 25 | 5 | 9 | 13 |
| mmu-miR-20a-5p | 1,085 | 1,344 | 1,263 | 1,106 |
| mmu-miR-20b-3p | 5 | 0 | 5 | 0 |
| mmu-miR-20b-5p | 190 | 151 | 173 | 327 |
| mmu-miR-21a-3p | 10 | 16 | 13 | 10 |
| mmu-miR-21a-5p | 1,854 | 14,884 | 7,557 | 12,759 |
| mmu-miR-21b | 0 | 0 | 0 | 0 |
| mmu-miR-21c | 0 | 0 | 0 | 1 |
| mmu-miR-22-3p | 2,525 | 3,855 | 3,009 | 3,238 |
| mmu-miR-22-5p | 226 | 140 | 91 | 142 |
| mmu-miR-23a-3p | 9,872 | 8,650 | 7,058 | 9,862 |
| mmu-miR-23a-5p | 5 | 17 | 10 | 14 |
| mmu-miR-23b-3p | 8,921 | 7,733 | 6,397 | 9,670 |
| mmu-miR-23b-5p | 0 | 3 | 6 | 9 |
| mmu-miR-24-1-5p | 12 | 12 | 13 | 23 |
| mmu-miR-24-2-5p | 423 | 281 | 229 | 344 |
| mmu-miR-24-3p | 10,589 | 8,055 | 10,035 | 10,807 |
| mmu-miR-25-3p | 822 | 1,621 | 663 | 797 |
| mmu-miR-25-5p | 1 | 8 | 5 | 3 |
| mmu-miR-26a-1-3p | 0 | 2 | 0 | 0 |
| mmu-miR-26a-2-3p | 0 | 6 | 1 | 9 |
| mmu-miR-26a-5p | 11,763 | 10,225 | 7,233 | 11,588 |
| mmu-miR-26b-3p | 0 | 5 | 3 | 2 |
| mmu-miR-26b-5p | 1,754 | 2,576 | 1,400 | 2,630 |
| mmu-miR-27a-3p | 2,228 | 2,693 | 1,880 | 3,033 |
| mmu-miR-27a-5p | 1 | 11 | 10 | 7 |
| mmu-miR-27b-3p | 1,997 | 1,416 | 1,436 | 2,337 |
| mmu-miR-27b-5p | 0 | 13 | 8 | 7 |
| mmu-miR-28a-3p | 36 | 50 | 42 | 50 |
| mmu-miR-28a-5p | 86 | 56 | 78 | 121 |
| mmu-miR-28b | 0 | 10 | 5 | 38 |
| mmu-miR-28c | 228 | 97 | 148 | 195 |
| mmu-miR-29a-3p | 4,180 | 4,394 | 2,906 | 3,348 |
| mmu-miR-29a-5p | 0 | 2 | 0 | 10 |
| mmu-miR-29b-1-5p | 0 | 0 | 1 | 2 |
| mmu-miR-29b-2-5p | 2 | 0 | 1 | 4 |
| mmu-miR-29b-3p | 560 | 1,420 | 766 | 2,043 |
| mmu-miR-29c-3p | 236 | 221 | 95 | 306 |
| mmu-miR-29c-5p | 25 | 5 | 8 | 18 |
| mmu-miR-30a-3p | 175 | 29 | 36 | 83 |
| mmu-miR-30a-5p | 2,843 | 2,287 | 2,743 | 3,100 |
| mmu-miR-30b-3p | 1 | 15 | 3 | 13 |
| mmu-miR-30b-5p | 3,482 | 3,055 | 2,061 | 3,893 |
| mmu-miR-30c-1-3p | 261 | 12,479 | 1,417 | 926 |
| mmu-miR-30c-2-3p | 16 | 327 | 17 | 53 |
| mmu-miR-30c-5p | 4,390 | 2,374 | 2,425 | 3,848 |
| mmu-miR-30d-3p | 0 | 5 | 2 | 17 |
| mmu-miR-30d-5p | 1,809 | 1,893 | 2,569 | 2,289 |
| mmu-miR-30e-3p | 118 | 33 | 26 | 99 |
| mmu-miR-30e-5p | 1,156 | 877 | 819 | 1,625 |
| mmu-miR-30f | 0 | 11 | 76 | 56 |
| mmu-miR-31-3p | 127 | 23 | 35 | 107 |
| mmu-miR-31-5p | 2,518 | 537 | 1,572 | 1,245 |
| mmu-miR-32-3p | 80 | 52 | 130 | 74 |
| mmu-miR-32-5p | 3 | 3 | 1 | 18 |
| mmu-miR-33-3p | 5 | 2 | 8 | 10 |
| mmu-miR-33-5p | 0 | 0 | 0 | 3 |
| mmu-miR-34a-3p | 14 | 9 | 7 | 9 |
| mmu-miR-34a-5p | 536 | 253 | 341 | 356 |
| mmu-miR-34b-3p | 15 | 26 | 34 | 42 |
| mmu-miR-34b-5p | 2 | 11 | 19 | 24 |
| mmu-miR-34c-3p | 116 | 105 | 110 | 133 |
| mmu-miR-34c-5p | 9 | 32 | 60 | 47 |
| mmu-miR-92a-1-5p | 3 | 4 | 0 | 1 |
| mmu-miR-92a-2-5p | 4 | 6 | 0 | 5 |
| mmu-miR-92a-3p | 922 | 1,083 | 1,044 | 494 |
| mmu-miR-92b-3p | 521 | 494 | 656 | 293 |
| mmu-miR-92b-5p | 5 | 11 | 1 | 20 |
| mmu-miR-93-3p | 23 | 17 | 18 | 22 |
| mmu-miR-93-5p | 1,064 | 1,301 | 1,191 | 864 |
| mmu-miR-96-3p | 0 | 0 | 0 | 1 |
| mmu-miR-96-5p | 40 | 10 | 11 | 87 |
| mmu-miR-98-3p | 2 | 2 | 0 | 7 |
| mmu-miR-98-5p | 213 | 112 | 153 | 278 |
| mmu-miR-99a-3p | 2 | 1 | 1 | 10 |
| mmu-miR-99a-5p | 3,054 | 2,319 | 2,314 | 3,237 |
| mmu-miR-99b-3p | 11 | 18 | 11 | 18 |
| mmu-miR-99b-5p | 1,046 | 784 | 1,079 | 719 |
| mmu-miR-100-3p | 0 | 0 | 0 | 0 |
| mmu-miR-100-5p | 1,323 | 910 | 913 | 2,029 |
| mmu-miR-101a-3p | 120 | 67 | 22 | 144 |
| mmu-miR-101a-5p | 1 | 1 | 0 | 3 |
| mmu-miR-101b-3p | 58 | 51 | 17 | 72 |
| mmu-miR-101b-5p | 0 | 0 | 0 | 0 |
| mmu-miR-101c | 28 | 12 | 0 | 43 |
| mmu-miR-103-1-5p | 1 | 7 | 4 | 6 |
| mmu-miR-103-2-5p | 0 | 7 | 3 | 5 |
| mmu-miR-103-3p | 1,927 | 1,596 | 1,517 | 1,513 |
| mmu-miR-105 | 0 | 0 | 0 | 2 |
| mmu-miR-106a-3p | 0 | 3 | 0 | 0 |
| mmu-miR-106a-5p | 536 | 577 | 573 | 764 |
| mmu-miR-106b-3p | 23 | 34 | 27 | 35 |
| mmu-miR-106b-5p | 514 | 431 | 260 | 455 |
| mmu-miR-107-3p | 1,941 | 1,603 | 1,597 | 1,579 |
| mmu-miR-107-5p | 0 | 4 | 0 | 7 |
| mmu-miR-122-3p | 0 | 0 | 0 | 6 |
| mmu-miR-122-5p | 0 | 0 | 0 | 2 |
| mmu-miR-124-3p | 6 | 1 | 6 | 1 |
| mmu-miR-124-5p | 2 | 0 | 0 | 2 |
| mmu-miR-125a-3p | 34 | 25 | 28 | 16 |
| mmu-miR-125a-5p | 2,514 | 1,661 | 3,060 | 1,976 |
| mmu-miR-125b-1-3p | 5 | 1 | 1 | 0 |
| mmu-miR-125b-2-3p | 27 | 1 | 9 | 24 |
| mmu-miR-125b-5p | 7,168 | 4,845 | 6,837 | 6,353 |
| mmu-miR-126a-3p | 3,059 | 2,764 | 3,680 | 3,265 |
| mmu-miR-126a-5p | 0 | 0 | 0 | 0 |
| mmu-miR-126b-3p | 0 | 0 | 0 | 0 |
| mmu-miR-126b-5p | 0 | 0 | 0 | 0 |
| mmu-miR-127-3p | 388 | 413 | 1,069 | 793 |
| mmu-miR-127-5p | 8 | 0 | 12 | 14 |
| mmu-miR-128-1-5p | 5 | 0 | 1 | 6 |
| mmu-miR-128-2-5p | 2 | 0 | 0 | 2 |
| mmu-miR-128-3p | 239 | 64 | 83 | 93 |
| mmu-miR-129-1-3p | 7 | 19 | 1 | 0 |
| mmu-miR-129-2-3p | 18 | 31 | 5 | 0 |
| mmu-miR-129-5p | 24 | 14 | 16 | 20 |
| mmu-miR-129b-3p | 4 | 1 | 1 | 0 |
| mmu-miR-129b-5p | 6 | 14 | 6 | 0 |
| mmu-miR-130a-3p | 1,101 | 568 | 912 | 969 |
| mmu-miR-130a-5p | 5 | 0 | 0 | 2 |
| mmu-miR-130b-3p | 31 | 96 | 58 | 33 |
| mmu-miR-130b-5p | 4 | 1 | 2 | 0 |
| mmu-miR-130c | 3 | 0 | 0 | 0 |
| mmu-miR-132-3p | 88 | 117 | 100 | 66 |
| mmu-miR-132-5p | 1 | 3 | 5 | 0 |
| mmu-miR-133a-3p | 10,224 | 3,885 | 4,531 | 4,626 |
| mmu-miR-133a-5p | 281 | 20 | 26 | 49 |
| mmu-miR-133b-3p | 9,240 | 3,516 | 4,095 | 4,412 |
| mmu-miR-133b-5p | 1,760 | 4,962 | 727 | 25,238 |
| mmu-miR-133c | 4 | 1 | 3 | 1 |
| mmu-miR-134-3p | 11 | 0 | 3 | 7 |
| mmu-miR-134-5p | 34 | 64 | 88 | 58 |
| mmu-miR-135a-1-3p | 19 | 9 | 6 | 5 |
| mmu-miR-135a-2-3p | 9 | 6 | 4 | 5 |
| mmu-miR-135a-5p | 0 | 0 | 0 | 0 |
| mmu-miR-135b-3p | 5 | 0 | 3 | 0 |
| mmu-miR-135b-5p | 0 | 0 | 0 | 0 |
| mmu-miR-136-3p | 0 | 5 | 1 | 43 |
| mmu-miR-136-5p | 0 | 1 | 0 | 0 |
| mmu-miR-137-3p | 1 | 0 | 0 | 0 |
| mmu-miR-137-5p | 0 | 0 | 0 | 0 |
| mmu-miR-138-1-3p | 23 | 12 | 13 | 14 |
| mmu-miR-138-2-3p | 0 | 2 | 0 | 2 |
| mmu-miR-138-5p | 158 | 165 | 218 | 145 |
| mmu-miR-139-3p | 9 | 22 | 10 | 10 |
| mmu-miR-139-5p | 198 | 722 | 472 | 188 |
| mmu-miR-140-3p | 2,303 | 2,971 | 2,266 | 1,434 |
| mmu-miR-140-5p | 44 | 68 | 42 | 80 |
| mmu-miR-141-3p | 207 | 69 | 77 | 183 |
| mmu-miR-141-5p | 6 | 6 | 2 | 12 |
| mmu-miR-142a-3p | 1 | 152 | 44 | 288 |
| mmu-miR-142a-5p | 49 | 651 | 305 | 438 |
| mmu-miR-142b | 3 | 2 | 0 | 2 |
| mmu-miR-143-3p | 4,353 | 2,041 | 2,179 | 2,712 |
| mmu-miR-143-5p | 9 | 8 | 4 | 4 |
| mmu-miR-144-3p | 0 | 0 | 0 | 0 |
| mmu-miR-144-5p | 0 | 0 | 0 | 0 |
| mmu-miR-145a-3p | 113 | 37 | 23 | 70 |
| mmu-miR-145a-5p | 4,703 | 2,676 | 3,623 | 3,945 |
| mmu-miR-145b | 3,102 | 1,862 | 2,972 | 3,266 |
| mmu-miR-146a-3p | 0 | 0 | 0 | 0 |
| mmu-miR-146a-5p | 1,869 | 1,175 | 1,388 | 1,795 |
| mmu-miR-146b-3p | 0 | 8 | 3 | 4 |
| mmu-miR-146b-5p | 630 | 1,566 | 455 | 765 |
| mmu-miR-147-3p | 0 | 11 | 1 | 0 |
| mmu-miR-147-5p | 0 | 9 | 1 | 0 |
| mmu-miR-148a-3p | 481 | 348 | 313 | 793 |
| mmu-miR-148a-5p | 1 | 12 | 5 | 50 |
| mmu-miR-148b-3p | 265 | 146 | 114 | 240 |
| mmu-miR-148b-5p | 0 | 7 | 4 | 5 |
| mmu-miR-149-3p | 1,928 | 7,174 | 4,816 | 3,093 |
| mmu-miR-149-5p | 39 | 33 | 59 | 56 |
| mmu-miR-150-3p | 0 | 21 | 9 | 3 |
| mmu-miR-150-5p | 395 | 354 | 656 | 518 |
| mmu-miR-151-3p | 159 | 73 | 108 | 107 |
| mmu-miR-151-5p | 701 | 234 | 332 | 383 |
| mmu-miR-152-3p | 786 | 459 | 521 | 1,114 |
| mmu-miR-152-5p | 0 | 6 | 0 | 57 |
| mmu-miR-153-3p | 0 | 0 | 0 | 0 |
| mmu-miR-153-5p | 0 | 4 | 0 | 8 |
| mmu-miR-154-3p | 0 | 20 | 10 | 55 |
| mmu-miR-154-5p | 0 | 13 | 10 | 46 |
| mmu-miR-155-3p | 0 | 0 | 0 | 0 |
| mmu-miR-155-5p | 50 | 143 | 158 | 188 |
| mmu-miR-181a-1-3p | 0 | 8 | 3 | 61 |
| mmu-miR-181a-2-3p | 0 | 5 | 0 | 14 |
| mmu-miR-181a-5p | 1,869 | 1,706 | 1,771 | 1,699 |
| mmu-miR-181b-1-3p | 0 | 2 | 0 | 8 |
| mmu-miR-181b-2-3p | 0 | 0 | 0 | 0 |
| mmu-miR-181b-5p | 332 | 108 | 161 | 260 |
| mmu-miR-181c-3p | 1 | 6 | 4 | 29 |
| mmu-miR-181c-5p | 486 | 302 | 492 | 316 |
| mmu-miR-181d-3p | 0 | 0 | 0 | 4 |
| mmu-miR-181d-5p | 178 | 41 | 56 | 152 |
| mmu-miR-182-3p | 0 | 0 | 0 | 1 |
| mmu-miR-182-5p | 1,332 | 263 | 360 | 503 |
| mmu-miR-183-3p | 1 | 1 | 1 | 5 |
| mmu-miR-183-5p | 813 | 152 | 183 | 304 |
| mmu-miR-184-3p | 4 | 0 | 0 | 0 |
| mmu-miR-184-5p | 14 | 35 | 32 | 50 |
| mmu-miR-185-3p | 1 | 0 | 0 | 1 |
| mmu-miR-185-5p | 234 | 140 | 127 | 121 |
| mmu-miR-186-3p | 0 | 0 | 0 | 0 |
| mmu-miR-186-5p | 145 | 119 | 146 | 175 |
| mmu-miR-187-3p | 57 | 18 | 17 | 52 |
| mmu-miR-187-5p | 13 | 2 | 5 | 13 |
| mmu-miR-188-3p | 0 | 0 | 0 | 0 |
| mmu-miR-188-5p | 42 | 64 | 37 | 39 |
| mmu-miR-190a-3p | 0 | 0 | 0 | 0 |
| mmu-miR-190a-5p | 1 | 0 | 1 | 0 |
| mmu-miR-190b-3p | 1 | 0 | 1 | 0 |
| mmu-miR-190b-5p | 1 | 0 | 0 | 0 |
| mmu-miR-191-3p | 2 | 0 | 2 | 0 |
| mmu-miR-191-5p | 4,368 | 4,311 | 4,016 | 2,879 |
| mmu-miR-192-3p | 4 | 0 | 4 | 0 |
| mmu-miR-192-5p | 43 | 44 | 25 | 31 |
| mmu-miR-193a-3p | 799 | 304 | 468 | 565 |
| mmu-miR-193a-5p | 18 | 4 | 6 | 12 |
| mmu-miR-193b-3p | 443 | 102 | 210 | 268 |
| mmu-miR-193b-5p | 11 | 1 | 2 | 2 |
| mmu-miR-194-1-3p | 0 | 1 | 0 | 1 |
| mmu-miR-194-2-3p | 3 | 9 | 2 | 6 |
| mmu-miR-194-5p | 90 | 65 | 47 | 59 |
| mmu-miR-195a-3p | 16 | 34 | 21 | 22 |
| mmu-miR-195a-5p | 3,898 | 1,642 | 1,913 | 2,713 |
| mmu-miR-195b | 0 | 0 | 0 | 0 |
| mmu-miR-196a-1-3p | 0 | 2 | 0 | 27 |
| mmu-miR-196a-2-3p | 0 | 0 | 0 | 3 |
| mmu-miR-196a-5p | 0 | 19 | 9 | 125 |
| mmu-miR-196b-3p | 0 | 14 | 11 | 21 |
| mmu-miR-196b-5p | 0 | 4 | 0 | 34 |
| mmu-miR-199a-3p | 2,755 | 2,429 | 3,430 | 6,792 |
| mmu-miR-199a-5p | 1,296 | 1,356 | 1,909 | 4,096 |
| mmu-miR-199b-5p | 380 | 330 | 440 | 2,005 |
| mmu-miR-200a-3p | 1,796 | 563 | 380 | 1,009 |
| mmu-miR-200a-5p | 16 | 5 | 7 | 5 |
| mmu-miR-200b-3p | 1,944 | 834 | 569 | 1,101 |
| mmu-miR-200b-5p | 51 | 20 | 26 | 80 |
| mmu-miR-200c-3p | 2,276 | 6,903 | 1,886 | 1,437 |
| mmu-miR-200c-5p | 0 | 0 | 2 | 0 |
| mmu-miR-201-3p | 0 | 0 | 0 | 0 |
| mmu-miR-201-5p | 0 | 0 | 0 | 0 |
| mmu-miR-202-3p | 0 | 0 | 0 | 0 |
| mmu-miR-202-5p | 0 | 0 | 0 | 0 |
| mmu-miR-203-3p | 10,177 | 4,619 | 5,097 | 8,700 |
| mmu-miR-203-5p | 61 | 15 | 22 | 62 |
| mmu-miR-204-3p | 10 | 15 | 11 | 10 |
| mmu-miR-204-5p | 52 | 9 | 10 | 25 |
| mmu-miR-205-3p | 7 | 5 | 7 | 10 |
| mmu-miR-205-5p | 16,838 | 10,480 | 16,221 | 14,445 |
| mmu-miR-206-3p | 1,657 | 293 | 761 | 1,207 |
| mmu-miR-206-5p | 0 | 0 | 0 | 0 |
| mmu-miR-207 | 14 | 2 | 9 | 5 |
| mmu-miR-208a-3p | 1 | 0 | 0 | 0 |
| mmu-miR-208a-5p | 6 | 43 | 17 | 8 |
| mmu-miR-208b-3p | 1 | 0 | 0 | 0 |
| mmu-miR-208b-5p | 1 | 0 | 0 | 0 |
| mmu-miR-210-3p | 290 | 466 | 258 | 160 |
| mmu-miR-210-5p | 46 | 32 | 36 | 33 |
| mmu-miR-211-3p | 38 | 17 | 23 | 11 |
| mmu-miR-211-5p | 7 | 0 | 4 | 3 |
| mmu-miR-212-3p | 22 | 10 | 32 | 10 |
| mmu-miR-212-5p | 6 | 0 | 10 | 1 |
| mmu-miR-214-3p | 3,054 | 3,258 | 5,682 | 4,709 |
| mmu-miR-214-5p | 88 | 83 | 112 | 164 |
| mmu-miR-215-3p | 1 | 0 | 0 | 0 |
| mmu-miR-215-5p | 57 | 9 | 39 | 12 |
| mmu-miR-216a-3p | 2 | 0 | 1 | 0 |
| mmu-miR-216a-5p | 6 | 0 | 8 | 0 |
| mmu-miR-216b-3p | 0 | 0 | 0 | 0 |
| mmu-miR-216b-5p | 7 | 0 | 4 | 2 |
| mmu-miR-216c-3p | 1 | 0 | 3 | 0 |
| mmu-miR-216c-5p | 1 | 0 | 0 | 0 |
| mmu-miR-217-3p | 0 | 0 | 54 | 0 |
| mmu-miR-217-5p | 7 | 1 | 6 | 0 |
| mmu-miR-218-1-3p | 14 | 9 | 16 | 0 |
| mmu-miR-218-2-3p | 20 | 13 | 13 | 13 |
| mmu-miR-218-5p | 157 | 48 | 39 | 153 |
| mmu-miR-219a-1-3p | 20 | 10 | 15 | 3 |
| mmu-miR-219a-2-3p | 3 | 0 | 3 | 2 |
| mmu-miR-219a-5p | 2 | 2 | 1 | 30 |
| mmu-miR-219b-3p | 0 | 0 | 0 | 0 |
| mmu-miR-219b-5p | 5 | 0 | 5 | 31 |
| mmu-miR-219c-3p | 0 | 0 | 0 | 0 |
| mmu-miR-219c-5p | 22 | 13 | 9 | 36 |
| mmu-miR-221-3p | 1,121 | 2,059 | 1,027 | 927 |
| mmu-miR-221-5p | 0 | 5 | 0 | 2 |
| mmu-miR-222-3p | 773 | 1,170 | 617 | 522 |
| mmu-miR-222-5p | 6 | 11 | 7 | 7 |
| mmu-miR-223-3p | 142 | 4,066 | 1,953 | 1,795 |
| mmu-miR-223-5p | 2 | 46 | 16 | 9 |
| mmu-miR-224-3p | 0 | 1 | 0 | 0 |
| mmu-miR-224-5p | 37 | 24 | 29 | 25 |
| mmu-miR-290a-3p | 0 | 6 | 3 | 2 |
| mmu-miR-290a-5p | 0 | 52 | 27 | 35 |
| mmu-miR-290b-3p | 0 | 0 | 0 | 0 |
| mmu-miR-290b-5p | 0 | 1 | 0 | 0 |
| mmu-miR-291a-3p | 0 | 0 | 0 | 1 |
| mmu-miR-291a-5p | 0 | 4 | 1 | 3 |
| mmu-miR-291b-3p | 0 | 0 | 0 | 0 |
| mmu-miR-291b-5p | 0 | 8 | 4 | 6 |
| mmu-miR-292a-3p | 0 | 0 | 0 | 7 |
| mmu-miR-292a-5p | 0 | 61 | 11 | 19 |
| mmu-miR-292b-3p | 0 | 0 | 0 | 0 |
| mmu-miR-292b-5p | 0 | 0 | 0 | 1 |
| mmu-miR-293-3p | 0 | 0 | 0 | 5 |
| mmu-miR-293-5p | 0 | 0 | 0 | 2 |
| mmu-miR-294-3p | 0 | 0 | 0 | 1 |
| mmu-miR-294-5p | 0 | 5 | 2 | 3 |
| mmu-miR-295-3p | 0 | 0 | 0 | 0 |
| mmu-miR-295-5p | 0 | 8 | 2 | 4 |
| mmu-miR-296-3p | 5 | 14 | 6 | 25 |
| mmu-miR-296-5p | 6 | 21 | 30 | 10 |
| mmu-miR-297a-3p | 0 | 8 | 19 | 39 |
| mmu-miR-297a-5p | 0 | 21 | 11 | 26 |
| mmu-miR-297b-5p | 15 | 2 | 1 | 13 |
| mmu-miR-297c-5p | 0 | 1 | 0 | 11 |
| mmu-miR-298-3p | 0 | 4 | 0 | 7 |
| mmu-miR-298-5p | 2 | 31 | 4 | 20 |
| mmu-miR-299a-3p | 5 | 9 | 10 | 26 |
| mmu-miR-299a-5p | 6 | 24 | 61 | 100 |
| mmu-miR-299b-3p | 4 | 13 | 19 | 24 |
| mmu-miR-299b-5p | 11 | 23 | 53 | 68 |
| mmu-miR-300-3p | 13 | 34 | 100 | 53 |
| mmu-miR-300-5p | 0 | 0 | 0 | 3 |
| mmu-miR-301a-3p | 1 | 7 | 4 | 33 |
| mmu-miR-301a-5p | 0 | 0 | 0 | 0 |
| mmu-miR-301b-3p | 0 | 0 | 0 | 2 |
| mmu-miR-301b-5p | 0 | 0 | 0 | 0 |
| mmu-miR-302a-3p | 0 | 0 | 0 | 0 |
| mmu-miR-302a-5p | 0 | 0 | 0 | 0 |
| mmu-miR-302b-3p | 0 | 0 | 0 | 0 |
| mmu-miR-302b-5p | 0 | 0 | 0 | 0 |
| mmu-miR-302c-3p | 0 | 0 | 0 | 0 |
| mmu-miR-302c-5p | 0 | 0 | 2 | 0 |
| mmu-miR-302d-3p | 0 | 0 | 0 | 0 |
| mmu-miR-302d-5p | 2 | 0 | 1 | 0 |
| mmu-miR-320-3p | 1,268 | 941 | 761 | 484 |
| mmu-miR-320-5p | 3 | 2 | 10 | 10 |
| mmu-miR-322-3p | 41 | 22 | 37 | 42 |
| mmu-miR-322-5p | 131 | 218 | 243 | 370 |
| mmu-miR-323-3p | 4 | 2 | 9 | 9 |
| mmu-miR-323-5p | 25 | 11 | 14 | 9 |
| mmu-miR-324-3p | 44 | 25 | 29 | 29 |
| mmu-miR-324-5p | 172 | 114 | 126 | 95 |
| mmu-miR-325-3p | 1 | 1 | 1 | 0 |
| mmu-miR-325-5p | 4 | 0 | 0 | 6 |
| mmu-miR-326-3p | 36 | 31 | 39 | 29 |
| mmu-miR-326-5p | 11 | 3 | 9 | 3 |
| mmu-miR-327 | 12 | 17 | 12 | 11 |
| mmu-miR-328-3p | 69 | 39 | 56 | 31 |
| mmu-miR-328-5p | 231 | 558 | 380 | 331 |
| mmu-miR-329-3p | 400 | 410 | 881 | 732 |
| mmu-miR-329-5p | 1 | 19 | 23 | 32 |
| mmu-miR-330-3p | 31 | 16 | 28 | 15 |
| mmu-miR-330-5p | 13 | 4 | 8 | 7 |
| mmu-miR-331-3p | 80 | 47 | 72 | 65 |
| mmu-miR-331-5p | 16 | 4 | 10 | 6 |
| mmu-miR-335-3p | 2 | 3 | 7 | 34 |
| mmu-miR-335-5p | 148 | 46 | 110 | 349 |
| mmu-miR-337-3p | 7 | 46 | 31 | 42 |
| mmu-miR-337-5p | 48 | 27 | 77 | 103 |
| mmu-miR-338-3p | 12 | 9 | 5 | 22 |
| mmu-miR-338-5p | 50 | 47 | 27 | 9 |
| mmu-miR-339-3p | 16 | 12 | 21 | 13 |
| mmu-miR-339-5p | 21 | 31 | 43 | 29 |
| mmu-miR-340-3p | 11 | 65 | 26 | 32 |
| mmu-miR-340-5p | 13 | 307 | 124 | 196 |
| mmu-miR-341-3p | 79 | 83 | 50 | 118 |
| mmu-miR-341-5p | 38,658 | 59,133 | 56,047 | 42,205 |
| mmu-miR-342-3p | 464 | 580 | 560 | 653 |
| mmu-miR-342-5p | 4 | 13 | 9 | 6 |
| mmu-miR-343 | 2 | 8 | 6 | 5 |
| mmu-miR-344-3p | 1 | 5 | 6 | 3 |
| mmu-miR-344-5p | 0 | 6 | 3 | 15 |
| mmu-miR-344b-3p | 0 | 2 | 4 | 0 |
| mmu-miR-344b-5p | 1 | 3 | 2 | 7 |
| mmu-miR-344c-3p | 0 | 3 | 4 | 4 |
| mmu-miR-344c-5p | 5 | 11 | 9 | 32 |
| mmu-miR-344d-1-5p | 0 | 4 | 3 | 15 |
| mmu-miR-344d-2-5p | 0 | 0 | 0 | 0 |
| mmu-miR-344d-3-5p | 0 | 5 | 1 | 15 |
| mmu-miR-344d-3p | 1 | 7 | 6 | 8 |
| mmu-miR-344e-3p | 0 | 0 | 1 | 4 |
| mmu-miR-344e-5p | 0 | 2 | 2 | 9 |
| mmu-miR-344f-3p | 3 | 10 | 5 | 17 |
| mmu-miR-344f-5p | 2 | 9 | 4 | 12 |
| mmu-miR-344g-3p | 3 | 11 | 6 | 14 |
| mmu-miR-344g-5p | 19 | 40 | 37 | 104 |
| mmu-miR-344h-3p | 1 | 6 | 3 | 35 |
| mmu-miR-344i | 7 | 18 | 6 | 31 |
| mmu-miR-345-3p | 12 | 19 | 18 | 30 |
| mmu-miR-345-5p | 71 | 58 | 60 | 114 |
| mmu-miR-346-3p | 76 | 376 | 203 | 189 |
| mmu-miR-346-5p | 211 | 126 | 138 | 70 |
| mmu-miR-350-3p | 128 | 171 | 100 | 215 |
| mmu-miR-350-5p | 10 | 10 | 10 | 2 |
| mmu-miR-351-3p | 12 | 15 | 23 | 2 |
| mmu-miR-351-5p | 59 | 73 | 173 | 108 |
| mmu-miR-361-3p | 22 | 30 | 31 | 22 |
| mmu-miR-361-5p | 352 | 325 | 226 | 122 |
| mmu-miR-362-3p | 19 | 58 | 27 | 67 |
| mmu-miR-362-5p | 91 | 121 | 98 | 89 |
| mmu-miR-363-3p | 0 | 2 | 0 | 0 |
| mmu-miR-363-5p | 78 | 59 | 7 | 418 |
| mmu-miR-365-1-5p | 2 | 39 | 14 | 16 |
| mmu-miR-365-2-5p | 5 | 14 | 9 | 10 |
| mmu-miR-365-3p | 109 | 40 | 32 | 211 |
| mmu-miR-367-3p | 0 | 0 | 0 | 0 |
| mmu-miR-367-5p | 0 | 1 | 0 | 0 |
| mmu-miR-369-3p | 0 | 0 | 1 | 16 |
| mmu-miR-369-5p | 3 | 5 | 12 | 27 |
| mmu-miR-370-3p | 10 | 13 | 14 | 8 |
| mmu-miR-370-5p | 0 | 1 | 1 | 6 |
| mmu-miR-374b-3p | 0 | 0 | 1 | 0 |
| mmu-miR-374b-5p | 125 | 149 | 101 | 244 |
| mmu-miR-374c-3p | 0 | 2 | 0 | 8 |
| mmu-miR-374c-5p | 47 | 79 | 51 | 160 |
| mmu-miR-375-3p | 32 | 8 | 34 | 6 |
| mmu-miR-375-5p | 13 | 11 | 12 | 11 |
| mmu-miR-376a-3p | 9 | 34 | 35 | 124 |
| mmu-miR-376a-5p | 1 | 0 | 0 | 3 |
| mmu-miR-376b-3p | 37 | 76 | 126 | 245 |
| mmu-miR-376b-5p | 2 | 8 | 11 | 32 |
| mmu-miR-376c-3p | 17 | 25 | 49 | 135 |
| mmu-miR-376c-5p | 1 | 0 | 4 | 7 |
| mmu-miR-377-3p | 8 | 9 | 26 | 64 |
| mmu-miR-377-5p | 8 | 2 | 5 | 5 |
| mmu-miR-378a-3p | 2,894 | 933 | 1,353 | 1,057 |
| mmu-miR-378a-5p | 282 | 69 | 136 | 85 |
| mmu-miR-378b | 2,142 | 586 | 1,001 | 824 |
| mmu-miR-378c | 2,317 | 663 | 1,079 | 864 |
| mmu-miR-378d | 1,740 | 490 | 858 | 757 |
| mmu-miR-379-3p | 16 | 24 | 37 | 69 |
| mmu-miR-379-5p | 114 | 82 | 237 | 250 |
| mmu-miR-380-3p | 8 | 5 | 10 | 22 |
| mmu-miR-380-5p | 4 | 1 | 4 | 0 |
| mmu-miR-381-3p | 37 | 52 | 123 | 130 |
| mmu-miR-381-5p | 0 | 2 | 2 | 13 |
| mmu-miR-382-3p | 1 | 0 | 6 | 25 |
| mmu-miR-382-5p | 11 | 11 | 34 | 25 |
| mmu-miR-383-3p | 33 | 12 | 16 | 19 |
| mmu-miR-383-5p | 10 | 3 | 8 | 1 |
| mmu-miR-384-3p | 0 | 0 | 0 | 0 |
| mmu-miR-384-5p | 2 | 0 | 2 | 0 |
| mmu-miR-409-3p | 34 | 40 | 43 | 90 |
| mmu-miR-409-5p | 0 | 11 | 34 | 53 |
| mmu-miR-410-3p | 0 | 27 | 58 | 74 |
| mmu-miR-410-5p | 0 | 0 | 0 | 4 |
| mmu-miR-411-3p | 22 | 57 | 79 | 167 |
| mmu-miR-411-5p | 65 | 58 | 141 | 199 |
| mmu-miR-412-3p | 5 | 14 | 14 | 11 |
| mmu-miR-412-5p | 0 | 0 | 3 | 2 |
| mmu-miR-421-3p | 26 | 46 | 52 | 37 |
| mmu-miR-421-5p | 0 | 0 | 0 | 0 |
| mmu-miR-423-3p | 7 | 22 | 25 | 23 |
| mmu-miR-423-5p | 277 | 244 | 265 | 86 |
| mmu-miR-425-3p | 14 | 32 | 34 | 19 |
| mmu-miR-425-5p | 366 | 287 | 312 | 262 |
| mmu-miR-429-3p | 1,318 | 377 | 258 | 634 |
| mmu-miR-429-5p | 0 | 0 | 0 | 2 |
| mmu-miR-431-3p | 1 | 12 | 10 | 22 |
| mmu-miR-431-5p | 10 | 28 | 57 | 68 |
| mmu-miR-432 | 0 | 8 | 1 | 6 |
| mmu-miR-433-3p | 6 | 30 | 36 | 40 |
| mmu-miR-433-5p | 0 | 10 | 1 | 14 |
| mmu-miR-434-3p | 31 | 65 | 100 | 236 |
| mmu-miR-434-5p | 0 | 14 | 28 | 63 |
| mmu-miR-448-3p | 0 | 1 | 0 | 3 |
| mmu-miR-448-5p | 0 | 4 | 0 | 4 |
| mmu-miR-449a-3p | 0 | 10 | 5 | 16 |
| mmu-miR-449a-5p | 0 | 1 | 0 | 11 |
| mmu-miR-449b | 0 | 7 | 1 | 14 |
| mmu-miR-449c-3p | 0 | 6 | 2 | 4 |
| mmu-miR-449c-5p | 0 | 4 | 2 | 4 |
| mmu-miR-450a-1-3p | 0 | 0 | 0 | 4 |
| mmu-miR-450a-2-3p | 0 | 0 | 0 | 1 |
| mmu-miR-450a-5p | 0 | 2 | 0 | 0 |
| mmu-miR-450b-3p | 1 | 3 | 0 | 1 |
| mmu-miR-450b-5p | 0 | 0 | 0 | 0 |
| mmu-miR-451a | 1,523 | 1,127 | 1,497 | 732 |
| mmu-miR-451b | 13 | 8 | 15 | 31 |
| mmu-miR-452-3p | 2 | 1 | 1 | 3 |
| mmu-miR-452-5p | 11 | 5 | 20 | 11 |
| mmu-miR-453 | 4 | 0 | 2 | 0 |
| mmu-miR-455-3p | 345 | 138 | 453 | 346 |
| mmu-miR-455-5p | 9 | 1 | 2 | 6 |
| mmu-miR-463-3p | 0 | 0 | 0 | 0 |
| mmu-miR-463-5p | 0 | 0 | 0 | 0 |
| mmu-miR-465a-3p | 1 | 0 | 0 | 0 |
| mmu-miR-465a-5p | 1 | 0 | 0 | 0 |
| mmu-miR-465b-5p | 2 | 0 | 0 | 0 |
| mmu-miR-465c-5p | 2 | 1 | 0 | 0 |
| mmu-miR-465d-3p | 1 | 0 | 0 | 0 |
| mmu-miR-465d-5p | 0 | 0 | 0 | 0 |
| mmu-miR-466a-3p | 17 | 19 | 18 | 20 |
| mmu-miR-466a-5p | 2 | 0 | 4 | 8 |
| mmu-miR-466b-3p | 11 | 16 | 22 | 22 |
| mmu-miR-466b-5p | 5 | 5 | 15 | 12 |
| mmu-miR-466c-5p | 41 | 42 | 95 | 81 |
| mmu-miR-466d-3p | 25 | 18 | 31 | 25 |
| mmu-miR-466d-5p | 6 | 0 | 4 | 0 |
| mmu-miR-466e-5p | 4 | 2 | 4 | 3 |
| mmu-miR-466f | 20 | 29 | 35 | 20 |
| mmu-miR-466f-3p | 693 | 552 | 876 | 671 |
| mmu-miR-466f-5p | 30 | 34 | 52 | 40 |
| mmu-miR-466g | 299 | 211 | 408 | 231 |
| mmu-miR-466h-3p | 556 | 550 | 836 | 482 |
| mmu-miR-466h-5p | 13 | 13 | 12 | 9 |
| mmu-miR-466i-3p | 308 | 288 | 503 | 405 |
| mmu-miR-466i-5p | 1,984 | 2,499 | 2,730 | 1,805 |
| mmu-miR-466j | 19 | 29 | 31 | 30 |
| mmu-miR-466k | 1 | 0 | 0 | 0 |
| mmu-miR-466l-3p | 0 | 0 | 8 | 0 |
| mmu-miR-466l-5p | 0 | 0 | 0 | 0 |
| mmu-miR-466m-3p | 157 | 112 | 236 | 239 |
| mmu-miR-466m-5p | 150 | 110 | 170 | 153 |
| mmu-miR-466n-3p | 0 | 0 | 0 | 0 |
| mmu-miR-466n-5p | 0 | 2 | 0 | 4 |
| mmu-miR-466o-3p | 0 | 0 | 8 | 9 |
| mmu-miR-466p-5p | 0 | 0 | 5 | 22 |
| mmu-miR-466q | 175 | 150 | 336 | 223 |
| mmu-miR-467a-3p | 68 | 41 | 105 | 86 |
| mmu-miR-467a-5p | 0 | 17 | 13 | 19 |
| mmu-miR-467b-3p | 312 | 214 | 445 | 308 |
| mmu-miR-467b-5p | 3 | 10 | 8 | 20 |
| mmu-miR-467c-3p | 26 | 22 | 69 | 57 |
| mmu-miR-467c-5p | 4 | 12 | 4 | 12 |
| mmu-miR-467d-3p | 193 | 130 | 291 | 206 |
| mmu-miR-467d-5p | 2 | 6 | 3 | 7 |
| mmu-miR-467e-3p | 0 | 11 | 45 | 55 |
| mmu-miR-467e-5p | 0 | 11 | 3 | 14 |
| mmu-miR-467f | 289 | 299 | 506 | 401 |
| mmu-miR-467g | 14 | 24 | 60 | 63 |
| mmu-miR-467h | 0 | 9 | 4 | 8 |
| mmu-miR-468-3p | 34 | 41 | 53 | 33 |
| mmu-miR-468-5p | 0 | 0 | 0 | 0 |
| mmu-miR-470-3p | 0 | 1 | 0 | 0 |
| mmu-miR-470-5p | 5 | 15 | 9 | 40 |
| mmu-miR-471-3p | 0 | 0 | 0 | 0 |
| mmu-miR-471-5p | 1 | 0 | 3 | 2 |
| mmu-miR-483-3p | 24 | 11 | 16 | 8 |
| mmu-miR-483-5p | 241 | 825 | 392 | 188 |
| mmu-miR-484 | 297 | 163 | 289 | 152 |
| mmu-miR-485-3p | 48 | 59 | 83 | 51 |
| mmu-miR-485-5p | 14 | 6 | 13 | 7 |
| mmu-miR-486a-3p | 310 | 106 | 82 | 389 |
| mmu-miR-486a-5p | 511 | 75 | 195 | 68 |
| mmu-miR-486b-3p | 75 | 18 | 31 | 48 |
| mmu-miR-487b-3p | 16 | 9 | 32 | 33 |
| mmu-miR-487b-5p | 7 | 1 | 4 | 2 |
| mmu-miR-488-3p | 0 | 0 | 0 | 0 |
| mmu-miR-488-5p | 2 | 0 | 1 | 0 |
| mmu-miR-489-3p | 89 | 20 | 48 | 72 |
| mmu-miR-489-5p | 0 | 0 | 1 | 0 |
| mmu-miR-490-3p | 4 | 5 | 11 | 0 |
| mmu-miR-490-5p | 9 | 3 | 7 | 2 |
| mmu-miR-491-3p | 0 | 0 | 4 | 0 |
| mmu-miR-491-5p | 3 | 2 | 7 | 4 |
| mmu-miR-493-3p | 29 | 17 | 20 | 22 |
| mmu-miR-493-5p | 0 | 0 | 2 | 2 |
| mmu-miR-494-3p | 1,106 | 1,347 | 1,787 | 2,220 |
| mmu-miR-494-5p | 1 | 0 | 1 | 2 |
| mmu-miR-495-3p | 18 | 31 | 60 | 65 |
| mmu-miR-495-5p | 0 | 0 | 0 | 7 |
| mmu-miR-496a-3p | 1 | 1 | 10 | 24 |
| mmu-miR-496a-5p | 0 | 0 | 0 | 4 |
| mmu-miR-496b | 0 | 0 | 0 | 0 |
| mmu-miR-497a-3p | 0 | 0 | 0 | 0 |
| mmu-miR-497a-5p | 771 | 274 | 389 | 630 |
| mmu-miR-497b | 0 | 2 | 8 | 5 |
| mmu-miR-499-3p | 0 | 0 | 4 | 1 |
| mmu-miR-499-5p | 0 | 0 | 0 | 6 |
| mmu-miR-500-3p | 116 | 218 | 207 | 132 |
| mmu-miR-500-5p | 0 | 1 | 5 | 4 |
| mmu-miR-501-3p | 125 | 254 | 229 | 140 |
| mmu-miR-501-5p | 0 | 26 | 17 | 13 |
| mmu-miR-503-3p | 2 | 18 | 26 | 26 |
| mmu-miR-503-5p | 22 | 76 | 148 | 120 |
| mmu-miR-504-3p | 27 | 77 | 61 | 67 |
| mmu-miR-504-5p | 0 | 8 | 13 | 10 |
| mmu-miR-505-3p | 0 | 3 | 3 | 0 |
| mmu-miR-505-5p | 0 | 8 | 8 | 9 |
| mmu-miR-509-3p | 0 | 0 | 0 | 0 |
| mmu-miR-509-5p | 0 | 0 | 1 | 3 |
| mmu-miR-511-3p | 85 | 178 | 52 | 143 |
| mmu-miR-511-5p | 0 | 5 | 1 | 18 |
| mmu-miR-532-3p | 60 | 51 | 53 | 31 |
| mmu-miR-532-5p | 234 | 301 | 224 | 115 |
| mmu-miR-539-3p | 0 | 0 | 0 | 0 |
| mmu-miR-539-5p | 9 | 10 | 22 | 15 |
| mmu-miR-540-3p | 12 | 16 | 30 | 14 |
| mmu-miR-540-5p | 13 | 2 | 8 | 3 |
| mmu-miR-541-3p | 9 | 2 | 2 | 18 |
| mmu-miR-541-5p | 49 | 107 | 266 | 192 |
| mmu-miR-542-3p | 0 | 0 | 0 | 0 |
| mmu-miR-542-5p | 7 | 2 | 3 | 0 |
| mmu-miR-543-3p | 7 | 5 | 10 | 13 |
| mmu-miR-543-5p | 0 | 1 | 1 | 5 |
| mmu-miR-544-3p | 2 | 0 | 0 | 0 |
| mmu-miR-544-5p | 29 | 21 | 14 | 21 |
| mmu-miR-546 | 17 | 14 | 6 | 16 |
| mmu-miR-547-3p | 7 | 4 | 1 | 4 |
| mmu-miR-547-5p | 6 | 7 | 1 | 0 |
| mmu-miR-551b-3p | 1 | 1 | 0 | 2 |
| mmu-miR-551b-5p | 4 | 3 | 0 | 0 |
| mmu-miR-568 | 8 | 1 | 24 | 2 |
| mmu-miR-574-3p | 247 | 242 | 303 | 237 |
| mmu-miR-574-5p | 434 | 382 | 649 | 337 |
| mmu-miR-582-3p | 12 | 2 | 7 | 4 |
| mmu-miR-582-5p | 16 | 6 | 5 | 12 |
| mmu-miR-590-3p | 1 | 0 | 0 | 0 |
| mmu-miR-590-5p | 1 | 0 | 0 | 0 |
| mmu-miR-592-3p | 3 | 1 | 5 | 3 |
| mmu-miR-592-5p | 1 | 0 | 0 | 0 |
| mmu-miR-598-3p | 2 | 3 | 7 | 1 |
| mmu-miR-598-5p | 18 | 14 | 11 | 5 |
| mmu-miR-599 | 0 | 0 | 1 | 0 |
| mmu-miR-615-3p | 28 | 22 | 49 | 37 |
| mmu-miR-615-5p | 135 | 90 | 71 | 310 |
| mmu-miR-652-3p | 762 | 812 | 697 | 350 |
| mmu-miR-652-5p | 29 | 77 | 25 | 39 |
| mmu-miR-653-3p | 0 | 1 | 0 | 2 |
| mmu-miR-653-5p | 0 | 6 | 0 | 0 |
| mmu-miR-654-3p | 15 | 8 | 11 | 7 |
| mmu-miR-654-5p | 4 | 0 | 3 | 0 |
| mmu-miR-664-3p | 87 | 73 | 61 | 45 |
| mmu-miR-664-5p | 34 | 31 | 16 | 18 |
| mmu-miR-665-3p | 55 | 1,146 | 507 | 160 |
| mmu-miR-665-5p | 90 | 53 | 108 | 44 |
| mmu-miR-666-3p | 22 | 8 | 14 | 1 |
| mmu-miR-666-5p | 28 | 14 | 16 | 5 |
| mmu-miR-667-3p | 27 | 28 | 36 | 25 |
| mmu-miR-667-5p | 38 | 27 | 31 | 11 |
| mmu-miR-668-3p | 288 | 763 | 519 | 1,644 |
| mmu-miR-668-5p | 14 | 12 | 11 | 9 |
| mmu-miR-669a-3-3p | 32 | 17 | 87 | 43 |
| mmu-miR-669a-3p | 208 | 154 | 357 | 230 |
| mmu-miR-669a-5p | 17 | 15 | 47 | 35 |
| mmu-miR-669b-3p | 6 | 4 | 14 | 11 |
| mmu-miR-669b-5p | 33 | 24 | 39 | 33 |
| mmu-miR-669c-3p | 163 | 198 | 385 | 300 |
| mmu-miR-669c-5p | 359 | 236 | 385 | 221 |
| mmu-miR-669d-2-3p | 9 | 6 | 16 | 21 |
| mmu-miR-669d-3p | 9 | 5 | 16 | 18 |
| mmu-miR-669d-5p | 7 | 2 | 8 | 5 |
| mmu-miR-669e-3p | 110 | 51 | 174 | 95 |
| mmu-miR-669e-5p | 7 | 7 | 10 | 8 |
| mmu-miR-669f-3p | 144 | 103 | 292 | 161 |
| mmu-miR-669f-5p | 125 | 92 | 180 | 128 |
| mmu-miR-669g | 1 | 1 | 1 | 0 |
| mmu-miR-669h-3p | 60 | 38 | 72 | 32 |
| mmu-miR-669h-5p | 3 | 1 | 4 | 0 |
| mmu-miR-669i | 20 | 13 | 22 | 10 |
| mmu-miR-669j | 2 | 0 | 0 | 0 |
| mmu-miR-669k-3p | 4 | 3 | 2 | 0 |
| mmu-miR-669k-5p | 29 | 24 | 45 | 43 |
| mmu-miR-669l-3p | 1 | 2 | 1 | 4 |
| mmu-miR-669l-5p | 27 | 39 | 78 | 77 |
| mmu-miR-669m-3p | 0 | 2 | 1 | 11 |
| mmu-miR-669n | 315 | 248 | 381 | 261 |
| mmu-miR-669o-5p | 16 | 32 | 64 | 67 |
| mmu-miR-669p-3p | 351 | 307 | 587 | 426 |
| mmu-miR-670-3p | 0 | 0 | 0 | 0 |
| mmu-miR-670-5p | 6 | 11 | 10 | 17 |
| mmu-miR-671-3p | 29 | 23 | 21 | 28 |
| mmu-miR-671-5p | 119 | 389 | 175 | 128 |
| mmu-miR-672-3p | 0 | 1 | 0 | 3 |
| mmu-miR-672-5p | 4 | 13 | 23 | 15 |
| mmu-miR-673-3p | 9 | 8 | 5 | 4 |
| mmu-miR-673-5p | 20 | 7 | 12 | 10 |
| mmu-miR-674-3p | 50 | 36 | 42 | 54 |
| mmu-miR-674-5p | 243 | 229 | 250 | 213 |
| mmu-miR-675-3p | 12 | 7 | 5 | 16 |
| mmu-miR-675-5p | 4 | 10 | 9 | 7 |
| mmu-miR-676-3p | 188 | 48 | 50 | 71 |
| mmu-miR-676-5p | 6 | 7 | 1 | 13 |
| mmu-miR-677-3p | 246 | 295 | 390 | 261 |
| mmu-miR-677-5p | 1 | 25 | 29 | 45 |
| mmu-miR-678 | 59 | 67 | 47 | 41 |
| mmu-miR-679-3p | 1 | 8 | 1 | 5 |
| mmu-miR-679-5p | 1 | 2 | 1 | 0 |
| mmu-miR-680 | 57 | 106 | 31 | 25 |
| mmu-miR-681 | 75 | 63 | 66 | 72 |
| mmu-miR-682 | 9 | 6 | 9 | 10 |
| mmu-miR-683 | 7 | 2 | 2 | 4 |
| mmu-miR-684 | 0 | 0 | 0 | 0 |
| mmu-miR-686 | 7 | 2 | 7 | 2 |
| mmu-miR-687 | 0 | 0 | 0 | 0 |
| mmu-miR-688 | 2 | 0 | 0 | 2 |
| mmu-miR-690 | 18,637 | 25,015 | 26,929 | 30,305 |
| mmu-miR-691 | 78 | 100 | 145 | 174 |
| mmu-miR-692 | 2 | 19 | 20 | 22 |
| mmu-miR-693-3p | 0 | 0 | 0 | 0 |
| mmu-miR-693-5p | 0 | 0 | 0 | 0 |
| mmu-miR-694 | 0 | 1 | 0 | 0 |
| mmu-miR-695 | 0 | 0 | 0 | 0 |
| mmu-miR-696 | 30 | 30 | 20 | 35 |
| mmu-miR-697 | 5 | 1 | 7 | 1 |
| mmu-miR-698-3p | 0 | 1 | 1 | 4 |
| mmu-miR-698-5p | 8 | 16 | 10 | 5 |
| mmu-miR-700-3p | 128 | 76 | 102 | 116 |
| mmu-miR-700-5p | 18 | 17 | 20 | 17 |
| mmu-miR-701-3p | 0 | 2 | 0 | 0 |
| mmu-miR-701-5p | 0 | 0 | 0 | 0 |
| mmu-miR-702-3p | 20 | 2 | 12 | 0 |
| mmu-miR-702-5p | 42 | 23 | 26 | 18 |
| mmu-miR-703 | 65 | 77 | 60 | 45 |
| mmu-miR-704 | 1 | 7 | 4 | 5 |
| mmu-miR-705 | 509 | 3,049 | 1,023 | 778 |
| mmu-miR-706 | 121 | 469 | 277 | 340 |
| mmu-miR-707 | 1 | 12 | 8 | 13 |
| mmu-miR-708-3p | 16 | 0 | 0 | 0 |
| mmu-miR-708-5p | 1,043 | 214 | 338 | 248 |
| mmu-miR-709 | 26,074 | 31,196 | 39,888 | 23,854 |
| mmu-miR-710 | 3 | 7 | 2 | 1 |
| mmu-miR-711 | 12 | 1,241 | 182 | 52 |
| mmu-miR-712-3p | 8 | 12 | 19 | 10 |
| mmu-miR-712-5p | 30 | 35 | 31 | 36 |
| mmu-miR-713 | 47 | 36 | 53 | 42 |
| mmu-miR-714 | 161 | 809 | 426 | 246 |
| mmu-miR-717 | 0 | 0 | 0 | 0 |
| mmu-miR-718 | 81 | 104 | 112 | 45 |
| mmu-miR-719 | 0 | 0 | 0 | 0 |
| mmu-miR-721 | 5 | 23 | 2 | 1 |
| mmu-miR-741-3p | 0 | 0 | 0 | 0 |
| mmu-miR-741-5p | 2 | 0 | 0 | 0 |
| mmu-miR-742-3p | 0 | 0 | 0 | 1 |
| mmu-miR-742-5p | 0 | 0 | 0 | 1 |
| mmu-miR-743a-3p | 0 | 0 | 0 | 0 |
| mmu-miR-743a-5p | 0 | 0 | 0 | 4 |
| mmu-miR-743b-3p | 0 | 0 | 0 | 0 |
| mmu-miR-743b-5p | 1 | 0 | 0 | 3 |
| mmu-miR-744-3p | 13 | 10 | 5 | 9 |
| mmu-miR-744-5p | 498 | 225 | 433 | 148 |
| mmu-miR-758-3p | 29 | 0 | 0 | 0 |
| mmu-miR-758-5p | 111 | 32 | 67 | 49 |
| mmu-miR-759 | 2 | 0 | 0 | 0 |
| mmu-miR-760-3p | 20 | 2 | 5 | 1 |
| mmu-miR-760-5p | 25 | 4 | 17 | 7 |
| mmu-miR-761 | 7 | 0 | 4 | 0 |
| mmu-miR-762 | 751 | 10,706 | 5,217 | 2,174 |
| mmu-miR-763 | 40 | 18 | 39 | 5 |
| mmu-miR-764-3p | 19 | 0 | 17 | 1 |
| mmu-miR-764-5p | 3 | 0 | 3 | 0 |
| mmu-miR-767 | 1 | 2 | 2 | 0 |
| mmu-miR-770-3p | 26 | 90 | 42 | 64 |
| mmu-miR-770-5p | 2 | 14 | 7 | 6 |
| mmu-miR-802-3p | 0 | 0 | 0 | 1 |
| mmu-miR-802-5p | 0 | 0 | 0 | 0 |
| mmu-miR-804 | 0 | 0 | 0 | 2 |
| mmu-miR-871-3p | 1 | 0 | 0 | 0 |
| mmu-miR-871-5p | 5 | 0 | 0 | 0 |
| mmu-miR-872-3p | 28 | 23 | 21 | 12 |
| mmu-miR-872-5p | 86 | 38 | 31 | 69 |
| mmu-miR-873a-3p | 2 | 0 | 0 | 0 |
| mmu-miR-873a-5p | 3 | 0 | 0 | 0 |
| mmu-miR-873b | 2 | 0 | 2 | 0 |
| mmu-miR-874-3p | 22 | 10 | 11 | 17 |
| mmu-miR-874-5p | 9 | 2 | 3 | 10 |
| mmu-miR-875-3p | 2 | 0 | 0 | 1 |
| mmu-miR-875-5p | 1 | 0 | 0 | 0 |
| mmu-miR-876-3p | 0 | 0 | 0 | 0 |
| mmu-miR-876-5p | 2 | 0 | 0 | 0 |
| mmu-miR-877-3p | 8 | 3 | 3 | 0 |
| mmu-miR-877-5p | 58 | 32 | 28 | 30 |
| mmu-miR-878-3p | 2 | 0 | 2 | 0 |
| mmu-miR-878-5p | 1 | 0 | 0 | 0 |
| mmu-miR-879-3p | 3 | 0 | 0 | 0 |
| mmu-miR-879-5p | 3 | 1 | 0 | 0 |
| mmu-miR-880-3p | 1 | 0 | 0 | 0 |
| mmu-miR-880-5p | 0 | 0 | 0 | 0 |
| mmu-miR-881-3p | 0 | 0 | 0 | 0 |
| mmu-miR-881-5p | 1 | 0 | 0 | 0 |
| mmu-miR-882 | 0 | 0 | 0 | 0 |
| mmu-miR-883a-3p | 1 | 0 | 1 | 0 |
| mmu-miR-883a-5p | 1 | 0 | 0 | 0 |
| mmu-miR-883b-3p | 0 | 0 | 0 | 0 |
| mmu-miR-883b-5p | 3 | 3 | 1 | 0 |
| mmu-miR-935 | 3 | 4 | 2 | 0 |
| mmu-miR-1187 | 112 | 115 | 213 | 174 |
| mmu-miR-1188-3p | 0 | 10 | 8 | 14 |
| mmu-miR-1188-5p | 5 | 37 | 8 | 42 |
| mmu-miR-1190 | 0 | 7 | 0 | 6 |
| mmu-miR-1191a | 0 | 0 | 0 | 4 |
| mmu-miR-1191b-3p | 0 | 0 | 0 | 4 |
| mmu-miR-1191b-5p | 16 | 60 | 35 | 51 |
| mmu-miR-1192 | 0 | 10 | 0 | 61 |
| mmu-miR-1193-3p | 0 | 0 | 0 | 3 |
| mmu-miR-1193-5p | 0 | 1 | 0 | 0 |
| mmu-miR-1194 | 0 | 1 | 0 | 0 |
| mmu-miR-1195 | 1,022 | 2,342 | 2,262 | 1,706 |
| mmu-miR-1197-3p | 0 | 0 | 0 | 0 |
| mmu-miR-1197-5p | 0 | 1 | 0 | 2 |
| mmu-miR-1198-3p | 0 | 0 | 0 | 0 |
| mmu-miR-1198-5p | 0 | 32 | 19 | 9 |
| mmu-miR-1199-3p | 2 | 15 | 10 | 18 |
| mmu-miR-1199-5p | 4 | 11 | 12 | 8 |
| mmu-miR-1224-3p | 0 | 5 | 0 | 13 |
| mmu-miR-1224-5p | 827 | 6,858 | 2,005 | 1,400 |
| mmu-miR-1231-3p | 0 | 9 | 7 | 14 |
| mmu-miR-1231-5p | 18 | 16 | 29 | 22 |
| mmu-miR-1247-3p | 18 | 6 | 7 | 3 |
| mmu-miR-1247-5p | 6 | 2 | 7 | 0 |
| mmu-miR-1249-3p | 19 | 23 | 45 | 4 |
| mmu-miR-1249-5p | 9 | 1 | 1 | 1 |
| mmu-miR-1251-3p | 15 | 3 | 2 | 15 |
| mmu-miR-1251-5p | 2 | 1 | 2 | 0 |
| mmu-miR-1258-3p | 1 | 0 | 0 | 3 |
| mmu-miR-1258-5p | 0 | 0 | 0 | 0 |
| mmu-miR-1264-3p | 1 | 0 | 0 | 1 |
| mmu-miR-1264-5p | 0 | 0 | 0 | 0 |
| mmu-miR-1291 | 16 | 5 | 8 | 7 |
| mmu-miR-1298-3p | 4 | 0 | 1 | 0 |
| mmu-miR-1298-5p | 3 | 0 | 0 | 0 |
| mmu-miR-1306-3p | 23 | 11 | 15 | 8 |
| mmu-miR-1306-5p | 35 | 11 | 20 | 23 |
| mmu-miR-1668 | 0 | 2 | 0 | 0 |
| mmu-miR-1839-3p | 255 | 160 | 111 | 231 |
| mmu-miR-1839-5p | 216 | 87 | 67 | 77 |
| mmu-miR-1843a-3p | 29 | 24 | 17 | 14 |
| mmu-miR-1843a-5p | 55 | 23 | 16 | 12 |
| mmu-miR-1843b-3p | 78 | 31 | 45 | 13 |
| mmu-miR-1843b-5p | 68 | 23 | 26 | 35 |
| mmu-miR-1892 | 138 | 468 | 117 | 92 |
| mmu-miR-1893 | 12 | 9 | 10 | 2 |
| mmu-miR-1894-3p | 136 | 611 | 274 | 257 |
| mmu-miR-1894-5p | 2 | 6 | 0 | 2 |
| mmu-miR-1895 | 301 | 441 | 379 | 201 |
| mmu-miR-1896 | 5 | 5 | 0 | 5 |
| mmu-miR-1897-3p | 0 | 0 | 0 | 0 |
| mmu-miR-1897-5p | 36 | 87 | 40 | 19 |
| mmu-miR-1898 | 3 | 0 | 2 | 2 |
| mmu-miR-1899 | 3 | 0 | 1 | 1 |
| mmu-miR-1900 | 8 | 0 | 7 | 2 |
| mmu-miR-1901 | 19 | 4 | 8 | 2 |
| mmu-miR-1902 | 6 | 0 | 12 | 3 |
| mmu-miR-1903 | 24 | 9 | 22 | 19 |
| mmu-miR-1904 | 19 | 9 | 11 | 6 |
| mmu-miR-1905 | 6 | 0 | 4 | 1 |
| mmu-miR-1906 | 89 | 55 | 62 | 69 |
| mmu-miR-1907 | 546 | 321 | 656 | 483 |
| mmu-miR-1912-3p | 3 | 0 | 4 | 0 |
| mmu-miR-1912-5p | 6 | 1 | 5 | 0 |
| mmu-miR-1927 | 6 | 0 | 2 | 0 |
| mmu-miR-1928 | 8 | 2 | 7 | 0 |
| mmu-miR-1929-3p | 7 | 0 | 7 | 1 |
| mmu-miR-1929-5p | 42 | 30 | 34 | 23 |
| mmu-miR-1930-3p | 19 | 7 | 8 | 3 |
| mmu-miR-1930-5p | 5 | 1 | 5 | 4 |
| mmu-miR-1931 | 15 | 21 | 15 | 20 |
| mmu-miR-1932 | 11 | 2 | 4 | 2 |
| mmu-miR-1933-3p | 1 | 0 | 1 | 0 |
| mmu-miR-1933-5p | 0 | 0 | 1 | 0 |
| mmu-miR-1934-3p | 142 | 582 | 273 | 169 |
| mmu-miR-1934-5p | 5 | 6 | 6 | 5 |
| mmu-miR-1936 | 2 | 2 | 2 | 2 |
| mmu-miR-1938 | 0 | 1 | 0 | 2 |
| mmu-miR-1941-3p | 0 | 0 | 0 | 0 |
| mmu-miR-1941-5p | 7 | 12 | 7 | 12 |
| mmu-miR-1942 | 0 | 0 | 0 | 2 |
| mmu-miR-1943-3p | 5 | 13 | 5 | 8 |
| mmu-miR-1943-5p | 20 | 16 | 12 | 7 |
| mmu-miR-1945 | 10 | 12 | 8 | 4 |
| mmu-miR-1946a | 10 | 12 | 16 | 9 |
| mmu-miR-1946b | 14 | 28 | 31 | 25 |
| mmu-miR-1947-3p | 4 | 5 | 2 | 3 |
| mmu-miR-1947-5p | 10 | 8 | 4 | 5 |
| mmu-miR-1948-3p | 6 | 11 | 8 | 14 |
| mmu-miR-1948-5p | 0 | 1 | 0 | 0 |
| mmu-miR-1949 | 120 | 492 | 167 | 200 |
| mmu-miR-1950 | 0 | 0 | 0 | 0 |
| mmu-miR-1951 | 14 | 22 | 10 | 23 |
| mmu-miR-1952 | 3 | 11 | 12 | 14 |
| mmu-miR-1953 | 1 | 4 | 2 | 1 |
| mmu-miR-1954 | 5 | 15 | 11 | 13 |
| mmu-miR-1955-3p | 0 | 7 | 1 | 0 |
| mmu-miR-1955-5p | 0 | 3 | 7 | 4 |
| mmu-miR-1956 | 21 | 74 | 65 | 52 |
| mmu-miR-1957a | 0 | 6 | 3 | 3 |
| mmu-miR-1957b | 0 | 7 | 1 | 12 |
| mmu-miR-1958 | 0 | 1 | 0 | 1 |
| mmu-miR-1960 | 0 | 15 | 5 | 8 |
| mmu-miR-1961 | 0 | 0 | 0 | 3 |
| mmu-miR-1962 | 0 | 6 | 8 | 31 |
| mmu-miR-1963 | 0 | 1 | 1 | 1 |
| mmu-miR-1964-3p | 0 | 41 | 12 | 21 |
| mmu-miR-1964-5p | 0 | 5 | 6 | 15 |
| mmu-miR-1966-3p | 0 | 1 | 0 | 3 |
| mmu-miR-1966-5p | 0 | 24 | 26 | 22 |
| mmu-miR-1967 | 0 | 23 | 10 | 14 |
| mmu-miR-1968-3p | 0 | 1 | 0 | 6 |
| mmu-miR-1968-5p | 0 | 11 | 12 | 8 |
| mmu-miR-1969 | 0 | 0 | 0 | 0 |
| mmu-miR-1970 | 0 | 7 | 0 | 2 |
| mmu-miR-1971 | 2 | 37 | 23 | 27 |
| mmu-miR-1981-3p | 0 | 8 | 0 | 5 |
| mmu-miR-1981-5p | 3 | 20 | 12 | 16 |
| mmu-miR-1982-3p | 0 | 5 | 0 | 9 |
| mmu-miR-1982-5p | 22 | 83 | 35 | 51 |
| mmu-miR-1983 | 22 | 85 | 77 | 102 |
| mmu-miR-2136 | 9 | 2 | 5 | 11 |
| mmu-miR-2137 | 1,653 | 9,262 | 8,878 | 4,982 |
| mmu-miR-2139 | 16 | 1 | 12 | 6 |
| mmu-miR-2183 | 2 | 2 | 6 | 0 |
| mmu-miR-2861 | 619 | 3,694 | 1,718 | 516 |
| mmu-miR-3057-3p | 10 | 0 | 10 | 8 |
| mmu-miR-3057-5p | 6 | 0 | 0 | 0 |
| mmu-miR-3058-3p | 23 | 6 | 10 | 12 |
| mmu-miR-3058-5p | 6 | 0 | 0 | 5 |
| mmu-miR-3059-3p | 24 | 53 | 33 | 10 |
| mmu-miR-3059-5p | 7 | 0 | 6 | 2 |
| mmu-miR-3060-3p | 28 | 7 | 18 | 19 |
| mmu-miR-3060-5p | 7 | 0 | 4 | 2 |
| mmu-miR-3061-3p | 11 | 0 | 9 | 2 |
| mmu-miR-3061-5p | 5 | 0 | 1 | 0 |
| mmu-miR-3062-3p | 5 | 0 | 6 | 7 |
| mmu-miR-3062-5p | 0 | 0 | 0 | 0 |
| mmu-miR-3063-3p | 5 | 0 | 5 | 0 |
| mmu-miR-3063-5p | 3 | 0 | 2 | 0 |
| mmu-miR-3064-3p | 35 | 8 | 13 | 45 |
| mmu-miR-3064-5p | 7 | 0 | 5 | 2 |
| mmu-miR-3065-3p | 3 | 0 | 1 | 0 |
| mmu-miR-3065-5p | 3 | 0 | 4 | 0 |
| mmu-miR-3066-3p | 13 | 8 | 9 | 12 |
| mmu-miR-3066-5p | 0 | 0 | 0 | 1 |
| mmu-miR-3067-3p | 32 | 22 | 24 | 11 |
| mmu-miR-3067-5p | 10 | 8 | 12 | 2 |
| mmu-miR-3068-3p | 672 | 1,033 | 462 | 472 |
| mmu-miR-3068-5p | 39 | 30 | 25 | 33 |
| mmu-miR-3069-3p | 13 | 12 | 13 | 14 |
| mmu-miR-3069-5p | 0 | 0 | 0 | 1 |
| mmu-miR-3070-2-3p | 160 | 91 | 48 | 326 |
| mmu-miR-3070-3p | 23 | 4 | 5 | 26 |
| mmu-miR-3070-5p | 10 | 3 | 7 | 13 |
| mmu-miR-3071-3p | 0 | 0 | 0 | 0 |
| mmu-miR-3071-5p | 12 | 16 | 24 | 10 |
| mmu-miR-3072-3p | 9 | 3 | 6 | 14 |
| mmu-miR-3072-5p | 1,443 | 1,769 | 1,361 | 868 |
| mmu-miR-3073a-3p | 1 | 0 | 0 | 3 |
| mmu-miR-3073a-5p | 6 | 2 | 5 | 3 |
| mmu-miR-3073b-3p | 10 | 7 | 9 | 18 |
| mmu-miR-3073b-5p | 1 | 1 | 3 | 0 |
| mmu-miR-3074-1-3p | 12 | 12 | 12 | 22 |
| mmu-miR-3074-2-3p | 13 | 7 | 6 | 18 |
| mmu-miR-3074-5p | 46 | 13 | 11 | 3 |
| mmu-miR-3075-3p | 2 | 1 | 0 | 2 |
| mmu-miR-3075-5p | 34 | 45 | 34 | 73 |
| mmu-miR-3076-3p | 4 | 2 | 4 | 4 |
| mmu-miR-3076-5p | 23 | 12 | 5 | 20 |
| mmu-miR-3077-3p | 12 | 6 | 5 | 12 |
| mmu-miR-3077-5p | 220 | 951 | 264 | 497 |
| mmu-miR-3078-3p | 0 | 0 | 0 | 5 |
| mmu-miR-3078-5p | 6 | 7 | 7 | 9 |
| mmu-miR-3079-3p | 2 | 0 | 1 | 0 |
| mmu-miR-3079-5p | 1 | 2 | 3 | 0 |
| mmu-miR-3080-3p | 7 | 5 | 6 | 11 |
| mmu-miR-3080-5p | 1 | 2 | 0 | 2 |
| mmu-miR-3081-3p | 5 | 6 | 6 | 10 |
| mmu-miR-3081-5p | 13 | 15 | 6 | 6 |
| mmu-miR-3082-3p | 4 | 4 | 5 | 1 |
| mmu-miR-3082-5p | 384 | 313 | 448 | 300 |
| mmu-miR-3083-3p | 0 | 5 | 1 | 8 |
| mmu-miR-3083-5p | 0 | 0 | 0 | 0 |
| mmu-miR-3084-3p | 144 | 137 | 125 | 112 |
| mmu-miR-3084-5p | 23 | 29 | 22 | 34 |
| mmu-miR-3085-3p | 6 | 10 | 11 | 11 |
| mmu-miR-3085-5p | 0 | 5 | 3 | 2 |
| mmu-miR-3086-3p | 0 | 11 | 6 | 11 |
| mmu-miR-3086-5p | 0 | 2 | 0 | 6 |
| mmu-miR-3087-3p | 0 | 1 | 0 | 1 |
| mmu-miR-3087-5p | 17 | 8 | 5 | 10 |
| mmu-miR-3088-3p | 0 | 5 | 2 | 2 |
| mmu-miR-3088-5p | 0 | 2 | 1 | 0 |
| mmu-miR-3089-3p | 0 | 1 | 1 | 5 |
| mmu-miR-3089-5p | 1 | 9 | 4 | 2 |
| mmu-miR-3090-3p | 2 | 6 | 6 | 5 |
| mmu-miR-3090-5p | 13 | 70 | 32 | 39 |
| mmu-miR-3091-3p | 0 | 5 | 3 | 4 |
| mmu-miR-3091-5p | 3 | 27 | 13 | 22 |
| mmu-miR-3092-3p | 0 | 54 | 10 | 17 |
| mmu-miR-3092-5p | 0 | 2 | 0 | 1 |
| mmu-miR-3093-3p | 1 | 20 | 10 | 9 |
| mmu-miR-3093-5p | 1 | 8 | 9 | 12 |
| mmu-miR-3094-3p | 0 | 0 | 0 | 1 |
| mmu-miR-3094-5p | 0 | 0 | 0 | 0 |
| mmu-miR-3095-3p | 15 | 93 | 97 | 58 |
| mmu-miR-3095-5p | 0 | 2 | 0 | 3 |
| mmu-miR-3097-3p | 0 | 3 | 0 | 5 |
| mmu-miR-3097-5p | 20 | 26 | 28 | 21 |
| mmu-miR-3098-3p | 0 | 18 | 18 | 25 |
| mmu-miR-3098-5p | 15 | 20 | 15 | 27 |
| mmu-miR-3099-3p | 0 | 44 | 29 | 25 |
| mmu-miR-3099-5p | 0 | 15 | 13 | 33 |
| mmu-miR-3100-3p | 4 | 14 | 13 | 21 |
| mmu-miR-3100-5p | 1 | 16 | 6 | 31 |
| mmu-miR-3101-3p | 0 | 15 | 20 | 30 |
| mmu-miR-3101-5p | 0 | 0 | 0 | 0 |
| mmu-miR-3102-3p | 25 | 14 | 21 | 37 |
| mmu-miR-3102-3p.2-3p | 9 | 9 | 10 | 9 |
| mmu-miR-3102-5p | 34 | 33 | 16 | 24 |
| mmu-miR-3102-5p.2-5p | 375 | 222 | 273 | 251 |
| mmu-miR-3103-3p | 1 | 0 | 0 | 0 |
| mmu-miR-3103-5p | 0 | 14 | 3 | 8 |
| mmu-miR-3104-3p | 12 | 5 | 21 | 10 |
| mmu-miR-3104-5p | 10 | 25 | 26 | 20 |
| mmu-miR-3105-3p | 0 | 0 | 0 | 2 |
| mmu-miR-3105-5p | 1 | 4 | 10 | 24 |
| mmu-miR-3106-3p | 0 | 0 | 0 | 3 |
| mmu-miR-3106-5p | 1 | 12 | 14 | 60 |
| mmu-miR-3108-3p | 0 | 0 | 0 | 0 |
| mmu-miR-3108-5p | 0 | 0 | 0 | 0 |
| mmu-miR-3109-3p | 0 | 0 | 0 | 0 |
| mmu-miR-3109-5p | 1 | 0 | 0 | 0 |
| mmu-miR-3110-3p | 147 | 85 | 90 | 40 |
| mmu-miR-3110-5p | 6 | 4 | 7 | 11 |
| mmu-miR-3112-3p | 0 | 0 | 0 | 0 |
| mmu-miR-3112-5p | 0 | 0 | 0 | 0 |
| mmu-miR-3113-3p | 12 | 10 | 12 | 15 |
| mmu-miR-3113-5p | 18 | 10 | 16 | 18 |
| mmu-miR-3154 | 32 | 75 | 24 | 36 |
| mmu-miR-3470a | 38 | 146 | 67 | 56 |
| mmu-miR-3470b | 55 | 156 | 62 | 53 |
| mmu-miR-3471 | 4 | 5 | 3 | 0 |
| mmu-miR-3472 | 11 | 6 | 6 | 2 |
| mmu-miR-3473a | 71 | 158 | 124 | 107 |
| mmu-miR-3473b | 1,198 | 5,909 | 2,581 | 2,061 |
| mmu-miR-3473c | 5 | 7 | 8 | 0 |
| mmu-miR-3473d | 21 | 46 | 33 | 49 |
| mmu-miR-3473e | 903 | 6,785 | 2,443 | 2,017 |
| mmu-miR-3473f | 451 | 1,698 | 645 | 784 |
| mmu-miR-3473g | 116 | 197 | 71 | 65 |
| mmu-miR-3474 | 25 | 70 | 30 | 26 |
| mmu-miR-3475-3p | 1 | 0 | 0 | 0 |
| mmu-miR-3475-5p | 6 | 4 | 8 | 0 |
| mmu-miR-3535 | 2,908 | 3,640 | 3,321 | 2,622 |
| mmu-miR-3544-3p | 12 | 9 | 13 | 1 |
| mmu-miR-3544-5p | 6 | 0 | 2 | 0 |
| mmu-miR-3547-3p | 18 | 9 | 18 | 4 |
| mmu-miR-3547-5p | 175 | 324 | 115 | 93 |
| mmu-miR-3552 | 19 | 15 | 17 | 9 |
| mmu-miR-3569-3p | 12 | 8 | 9 | 7 |
| mmu-miR-3569-5p | 160 | 109 | 110 | 111 |
| mmu-miR-3572-3p | 1 | 1 | 1 | 1 |
| mmu-miR-3572-5p | 5 | 10 | 8 | 2 |
| mmu-miR-3618-3p | 0 | 0 | 0 | 0 |
| mmu-miR-3618-5p | 0 | 0 | 0 | 0 |
| mmu-miR-3620-3p | 29 | 17 | 33 | 15 |
| mmu-miR-3620-5p | 564 | 1,773 | 882 | 705 |
| mmu-miR-3960 | 1,373 | 8,815 | 4,321 | 1,557 |
| mmu-miR-3961 | 1 | 0 | 4 | 10 |
| mmu-miR-3962 | 207 | 89 | 210 | 583 |
| mmu-miR-3963 | 888 | 1,563 | 829 | 1,777 |
| mmu-miR-3964 | 0 | 1 | 0 | 0 |
| mmu-miR-3965 | 0 | 1 | 0 | 79 |
| mmu-miR-3966 | 4 | 1 | 3 | 7 |
| mmu-miR-3967 | 0 | 1 | 1 | 1 |
| mmu-miR-3968 | 19 | 15 | 20 | 19 |
| mmu-miR-3969 | 0 | 0 | 1 | 0 |
| mmu-miR-3970 | 196 | 187 | 562 | 1,101 |
| mmu-miR-3971 | 0 | 8 | 9 | 8 |
| mmu-miR-5046 | 3 | 16 | 23 | 26 |
| mmu-miR-5098 | 0 | 5 | 4 | 5 |
| mmu-miR-5099 | 4,172 | 7,345 | 11,433 | 8,272 |
| mmu-miR-5100 | 567 | 863 | 1,042 | 838 |
| mmu-miR-5101 | 0 | 1 | 0 | 5 |
| mmu-miR-5103 | 0 | 8 | 5 | 10 |
| mmu-miR-5104 | 0 | 10 | 7 | 7 |
| mmu-miR-5106 | 0 | 10 | 3 | 11 |
| mmu-miR-5107-3p | 0 | 14 | 11 | 27 |
| mmu-miR-5107-5p | 781 | 897 | 1,565 | 1,138 |
| mmu-miR-5108 | 0 | 5 | 1 | 5 |
| mmu-miR-5110 | 0 | 24 | 27 | 32 |
| mmu-miR-5112 | 3,907 | 6,386 | 4,410 | 2,277 |
| mmu-miR-5113 | 0 | 35 | 32 | 31 |
| mmu-miR-5114 | 2 | 9 | 4 | 21 |
| mmu-miR-5116 | 0 | 9 | 8 | 12 |
| mmu-miR-5118 | 0 | 12 | 8 | 17 |
| mmu-miR-5119 | 105 | 450 | 125 | 90 |
| mmu-miR-5120 | 18 | 10 | 3 | 9 |
| mmu-miR-5121 | 1,147 | 886 | 2,000 | 943 |
| mmu-miR-5122 | 51 | 306 | 83 | 52 |
| mmu-miR-5123 | 2 | 1 | 0 | 1 |
| mmu-miR-5124a | 3 | 1 | 0 | 0 |
| mmu-miR-5124b | 2 | 0 | 2 | 0 |
| mmu-miR-5125 | 2 | 0 | 0 | 3 |
| mmu-miR-5126 | 3,779 | 17,578 | 10,696 | 5,054 |
| mmu-miR-5127 | 8 | 0 | 3 | 0 |
| mmu-miR-5128 | 62 | 136 | 61 | 42 |
| mmu-miR-5129-3p | 5 | 0 | 5 | 0 |
| mmu-miR-5129-5p | 3 | 10 | 2 | 0 |
| mmu-miR-5130 | 80 | 454 | 171 | 62 |
| mmu-miR-5131 | 31 | 21 | 16 | 13 |
| mmu-miR-5132-3p | 6 | 4 | 6 | 0 |
| mmu-miR-5132-5p | 40 | 75 | 53 | 46 |
| mmu-miR-5133 | 52 | 27 | 32 | 12 |
| mmu-miR-5134-3p | 10 | 1 | 6 | 0 |
| mmu-miR-5134-5p | 28 | 17 | 19 | 28 |
| mmu-miR-5135 | 20 | 8 | 8 | 2 |
| mmu-miR-5136 | 11 | 2 | 6 | 0 |
| mmu-miR-5615-3p | 0 | 4 | 0 | 0 |
| mmu-miR-5615-5p | 11 | 8 | 20 | 7 |
| mmu-miR-5616-3p | 25 | 16 | 25 | 9 |
| mmu-miR-5616-5p | 0 | 0 | 0 | 0 |
| mmu-miR-5617-3p | 1 | 6 | 4 | 11 |
| mmu-miR-5617-5p | 0 | 2 | 0 | 0 |
| mmu-miR-5618-3p | 0 | 1 | 0 | 7 |
| mmu-miR-5618-5p | 0 | 0 | 0 | 7 |
| mmu-miR-5619-3p | 0 | 0 | 0 | 2 |
| mmu-miR-5619-5p | 0 | 0 | 0 | 0 |
| mmu-miR-5620-3p | 3 | 13 | 16 | 1 |
| mmu-miR-5620-5p | 0 | 12 | 1 | 9 |
| mmu-miR-5621-3p | 1 | 11 | 5 | 13 |
| mmu-miR-5621-5p | 11 | 20 | 23 | 21 |
| mmu-miR-5622-3p | 49 | 67 | 49 | 57 |
| mmu-miR-5622-5p | 0 | 7 | 1 | 9 |
| mmu-miR-5623-3p | 0 | 2 | 1 | 4 |
| mmu-miR-5623-5p | 0 | 4 | 1 | 3 |
| mmu-miR-5624-3p | 0 | 1 | 0 | 0 |
| mmu-miR-5624-5p | 1 | 17 | 23 | 13 |
| mmu-miR-5625-3p | 0 | 3 | 4 | 0 |
| mmu-miR-5625-5p | 0 | 2 | 0 | 5 |
| mmu-miR-5626-3p | 0 | 5 | 2 | 2 |
| mmu-miR-5626-5p | 0 | 6 | 5 | 8 |
| mmu-miR-5627-3p | 35 | 35 | 28 | 8 |
| mmu-miR-5627-5p | 31 | 23 | 33 | 9 |
| mmu-miR-5709-3p | 8 | 4 | 10 | 3 |
| mmu-miR-5709-5p | 4 | 0 | 3 | 0 |
| mmu-miR-5710 | 3 | 1 | 1 | 0 |
| mmu-miR-6236 | 77 | 135 | 158 | 158 |
| mmu-miR-6237 | 0 | 0 | 0 | 1 |
| mmu-miR-6238 | 343 | 661 | 272 | 1,360 |
| mmu-miR-6239 | 39,029 | 70,523 | 65,905 | 56,333 |
| mmu-miR-6240 | 937 | 4,401 | 1,850 | 1,741 |
| mmu-miR-6241 | 7 | 7 | 16 | 7 |
| mmu-miR-6244 | 17 | 39 | 42 | 80 |
| mmu-miR-6335 | 0 | 0 | 2 | 0 |
| mmu-miR-6336 | 0 | 1 | 0 | 0 |
| mmu-miR-6337 | 2 | 5 | 6 | 8 |
| mmu-miR-6338 | 0 | 0 | 0 | 1 |
| mmu-miR-6339 | 0 | 0 | 0 | 0 |
| mmu-miR-6340 | 0 | 10 | 25 | 35 |
| mmu-miR-6341 | 0 | 0 | 0 | 0 |
| mmu-miR-6342 | 0 | 1 | 3 | 5 |
| mmu-miR-6343 | 0 | 3 | 2 | 1 |
| mmu-miR-6344 | 0 | 8 | 16 | 15 |
| mmu-miR-6345 | 0 | 4 | 8 | 4 |
| mmu-miR-6346 | 0 | 1 | 1 | 1 |
| mmu-miR-6347 | 12 | 32 | 30 | 18 |
| mmu-miR-6348 | 333 | 193 | 292 | 141 |
| mmu-miR-6349 | 145 | 751 | 167 | 122 |
| mmu-miR-6350 | 0 | 1 | 0 | 0 |
| mmu-miR-6351 | 2 | 17 | 20 | 16 |
| mmu-miR-6352 | 0 | 9 | 7 | 2 |
| mmu-miR-6353 | 0 | 0 | 0 | 0 |
| mmu-miR-6354 | 0 | 9 | 11 | 14 |
| mmu-miR-6355 | 0 | 2 | 0 | 0 |
| mmu-miR-6356 | 0 | 5 | 3 | 3 |
| mmu-miR-6357 | 0 | 4 | 3 | 7 |
| mmu-miR-6358 | 0 | 0 | 0 | 2 |
| mmu-miR-6359 | 0 | 11 | 12 | 19 |
| mmu-miR-6360 | 281 | 226 | 230 | 253 |
| mmu-miR-6361 | 0 | 11 | 4 | 19 |
| mmu-miR-6362 | 0 | 3 | 4 | 3 |
| mmu-miR-6363 | 0 | 5 | 1 | 3 |
| mmu-miR-6364 | 0 | 12 | 9 | 17 |
| mmu-miR-6365 | 0 | 1 | 0 | 1 |
| mmu-miR-6366 | 104 | 552 | 178 | 113 |
| mmu-miR-6367 | 0 | 8 | 4 | 18 |
| mmu-miR-6368 | 43 | 50 | 45 | 47 |
| mmu-miR-6369 | 0 | 8 | 1 | 9 |
| mmu-miR-6370 | 7 | 46 | 13 | 17 |
| mmu-miR-6371 | 0 | 0 | 0 | 4 |
| mmu-miR-6372 | 1 | 2 | 1 | 5 |
| mmu-miR-6373 | 0 | 0 | 0 | 0 |
| mmu-miR-6374 | 1 | 0 | 0 | 0 |
| mmu-miR-6375 | 0 | 0 | 0 | 0 |
| mmu-miR-6376 | 0 | 0 | 0 | 0 |
| mmu-miR-6377 | 3 | 1 | 1 | 0 |
| mmu-miR-6378 | 23 | 21 | 17 | 11 |
| mmu-miR-6379 | 0 | 0 | 2 | 0 |
| mmu-miR-6380 | 2 | 0 | 1 | 0 |
| mmu-miR-6381 | 7 | 2 | 3 | 0 |
| mmu-miR-6382 | 20 | 7 | 15 | 6 |
| mmu-miR-6383 | 4 | 0 | 1 | 0 |
| mmu-miR-6384 | 4 | 4 | 12 | 8 |
| mmu-miR-6385 | 162 | 77 | 105 | 53 |
| mmu-miR-6386 | 10 | 4 | 3 | 7 |
| mmu-miR-6387 | 2 | 0 | 1 | 0 |
| mmu-miR-6388 | 6 | 4 | 7 | 2 |
| mmu-miR-6389 | 0 | 0 | 2 | 0 |
| mmu-miR-6390 | 0 | 0 | 0 | 0 |
| mmu-miR-6391 | 13 | 12 | 10 | 9 |
| mmu-miR-6392-3p | 11 | 6 | 10 | 2 |
| mmu-miR-6392-5p | 12 | 11 | 18 | 20 |
| mmu-miR-6393 | 5 | 2 | 12 | 10 |
| mmu-miR-6394 | 48 | 66 | 35 | 32 |
| mmu-miR-6395 | 24 | 14 | 23 | 23 |
| mmu-miR-6396 | 0 | 1 | 1 | 5 |
| mmu-miR-6397 | 1 | 0 | 1 | 1 |
| mmu-miR-6398 | 1 | 3 | 3 | 0 |
| mmu-miR-6399 | 0 | 0 | 0 | 0 |
| mmu-miR-6400 | 7 | 5 | 7 | 6 |
| mmu-miR-6401 | 2 | 2 | 4 | 1 |
| mmu-miR-6402 | 32 | 15 | 38 | 117 |
| mmu-miR-6403 | 0 | 2 | 4 | 2 |
| mmu-miR-6404 | 1 | 7 | 1 | 5 |
| mmu-miR-6405 | 8 | 11 | 11 | 16 |
| mmu-miR-6406 | 0 | 3 | 5 | 20 |
| mmu-miR-6407 | 8 | 9 | 5 | 6 |
| mmu-miR-6408 | 1 | 4 | 3 | 3 |
| mmu-miR-6409 | 10 | 10 | 31 | 15 |
| mmu-miR-6410 | 0 | 1 | 1 | 4 |
| mmu-miR-6411 | 0 | 0 | 0 | 0 |
| mmu-miR-6412 | 0 | 5 | 0 | 6 |
| mmu-miR-6413 | 3 | 6 | 4 | 24 |
| mmu-miR-6414 | 0 | 3 | 0 | 1 |
| mmu-miR-6415 | 0 | 5 | 3 | 1 |
| mmu-miR-6416-3p | 3 | 35 | 25 | 27 |
| mmu-miR-6416-5p | 0 | 2 | 0 | 4 |
| mmu-miR-6417 | 3 | 15 | 17 | 76 |
| mmu-miR-6418-3p | 0 | 14 | 6 | 11 |
| mmu-miR-6418-5p | 25 | 160 | 65 | 46 |
| mmu-miR-6419 | 0 | 6 | 7 | 26 |
| mmu-miR-6420 | 0 | 3 | 0 | 0 |
| mmu-miR-6481 | 0 | 0 | 0 | 6 |
| mmu-miR-6516-3p | 15 | 36 | 17 | 39 |
| mmu-miR-6516-5p | 117 | 124 | 142 | 128 |
| mmu-miR-6537-3p | 0 | 7 | 0 | 0 |
| mmu-miR-6537-5p | 0 | 10 | 1 | 14 |
| mmu-miR-6538 | 607 | 3,041 | 1,573 | 943 |
| mmu-miR-6539 | 0 | 5 | 0 | 3 |
| mmu-miR-6540-3p | 0 | 0 | 0 | 0 |
| mmu-miR-6540-5p | 34 | 89 | 101 | 135 |
| mmu-miR-6541 | 6 | 1 | 4 | 0 |
| mmu-miR-6546-3p | 23 | 17 | 19 | 10 |
| mmu-miR-6546-5p | 7 | 1 | 5 | 3 |
| mmu-miR-6715-3p | 10 | 7 | 12 | 4 |
| mmu-miR-6715-5p | 0 | 4 | 0 | 8 |
| mmu-miR-6769b-3p | 4 | 11 | 6 | 4 |
| mmu-miR-6769b-5p | 28 | 72 | 20 | 36 |
| mmu-miR-6896-3p | 16 | 15 | 29 | 56 |
| mmu-miR-6896-5p | 14 | 3 | 8 | 6 |
| mmu-miR-6897-3p | 6 | 6 | 10 | 6 |
| mmu-miR-6897-5p | 4 | 1 | 6 | 2 |
| mmu-miR-6898-3p | 3 | 0 | 0 | 3 |
| mmu-miR-6898-5p | 19 | 11 | 6 | 8 |
| mmu-miR-6899-3p | 2 | 0 | 1 | 2 |
| mmu-miR-6899-5p | 25 | 15 | 16 | 16 |
| mmu-miR-6900-3p | 8 | 0 | 2 | 2 |
| mmu-miR-6900-5p | 8 | 7 | 9 | 3 |
| mmu-miR-6901-3p | 0 | 0 | 0 | 5 |
| mmu-miR-6901-5p | 24 | 10 | 12 | 10 |
| mmu-miR-6902-3p | 3 | 6 | 7 | 5 |
| mmu-miR-6902-5p | 0 | 0 | 0 | 0 |
| mmu-miR-6903-3p | 0 | 3 | 5 | 11 |
| mmu-miR-6903-5p | 0 | 0 | 0 | 0 |
| mmu-miR-6904-3p | 0 | 0 | 0 | 0 |
| mmu-miR-6904-5p | 0 | 2 | 1 | 0 |
| mmu-miR-6905-3p | 0 | 0 | 0 | 0 |
| mmu-miR-6905-5p | 1 | 2 | 2 | 0 |
| mmu-miR-6906-3p | 0 | 5 | 4 | 0 |
| mmu-miR-6906-5p | 36 | 49 | 45 | 42 |
| mmu-miR-6907-3p | 0 | 0 | 0 | 0 |
| mmu-miR-6907-5p | 36 | 35 | 31 | 23 |
| mmu-miR-6908-3p | 0 | 5 | 6 | 12 |
| mmu-miR-6908-5p | 19 | 48 | 25 | 31 |
| mmu-miR-6909-3p | 7 | 9 | 9 | 15 |
| mmu-miR-6909-5p | 2 | 12 | 7 | 8 |
| mmu-miR-6910-3p | 0 | 4 | 0 | 1 |
| mmu-miR-6910-5p | 10 | 25 | 18 | 28 |
| mmu-miR-6911-3p | 0 | 4 | 2 | 7 |
| mmu-miR-6911-5p | 0 | 12 | 5 | 8 |
| mmu-miR-6912-3p | 1 | 6 | 3 | 10 |
| mmu-miR-6912-5p | 130 | 99 | 73 | 55 |
| mmu-miR-6913-3p | 0 | 0 | 0 | 0 |
| mmu-miR-6913-5p | 0 | 5 | 6 | 18 |
| mmu-miR-6914-3p | 0 | 5 | 2 | 14 |
| mmu-miR-6914-5p | 4 | 10 | 7 | 7 |
| mmu-miR-6915-3p | 2 | 14 | 18 | 22 |
| mmu-miR-6915-5p | 0 | 9 | 10 | 13 |
| mmu-miR-6916-3p | 0 | 6 | 2 | 5 |
| mmu-miR-6916-5p | 0 | 7 | 2 | 14 |
| mmu-miR-6917-3p | 0 | 10 | 1 | 12 |
| mmu-miR-6917-5p | 4 | 166 | 15 | 27 |
| mmu-miR-6918-3p | 1 | 6 | 2 | 8 |
| mmu-miR-6918-5p | 0 | 3 | 0 | 2 |
| mmu-miR-6919-3p | 7 | 17 | 9 | 13 |
| mmu-miR-6919-5p | 0 | 0 | 1 | 4 |
| mmu-miR-6920-3p | 0 | 3 | 0 | 4 |
| mmu-miR-6920-5p | 0 | 1 | 0 | 0 |
| mmu-miR-6921-3p | 7 | 7 | 13 | 6 |
| mmu-miR-6921-5p | 12 | 23 | 15 | 17 |
| mmu-miR-6922-3p | 8 | 2 | 8 | 1 |
| mmu-miR-6922-5p | 26 | 23 | 21 | 6 |
| mmu-miR-6923-3p | 18 | 11 | 23 | 2 |
| mmu-miR-6923-5p | 26 | 27 | 11 | 16 |
| mmu-miR-6924-3p | 9 | 3 | 7 | 0 |
| mmu-miR-6924-5p | 7 | 0 | 0 | 6 |
| mmu-miR-6925-3p | 3 | 2 | 1 | 4 |
| mmu-miR-6925-5p | 29 | 241 | 77 | 42 |
| mmu-miR-6926-3p | 3 | 1 | 6 | 2 |
| mmu-miR-6926-5p | 35 | 42 | 22 | 22 |
| mmu-miR-6927-3p | 18 | 12 | 16 | 9 |
| mmu-miR-6927-5p | 23 | 24 | 17 | 10 |
| mmu-miR-6928-3p | 0 | 1 | 2 | 0 |
| mmu-miR-6928-5p | 6 | 0 | 3 | 2 |
| mmu-miR-6929-3p | 51 | 23 | 39 | 33 |
| mmu-miR-6929-5p | 12 | 10 | 18 | 36 |
| mmu-miR-6930-3p | 6 | 2 | 8 | 3 |
| mmu-miR-6930-5p | 12 | 8 | 15 | 10 |
| mmu-miR-6931-3p | 5 | 2 | 7 | 0 |
| mmu-miR-6931-5p | 472 | 9,852 | 1,094 | 605 |
| mmu-miR-6932-3p | 2 | 3 | 3 | 6 |
| mmu-miR-6932-5p | 4 | 2 | 2 | 4 |
| mmu-miR-6933-3p | 5 | 5 | 20 | 9 |
| mmu-miR-6933-5p | 4 | 0 | 6 | 16 |
| mmu-miR-6934-3p | 35 | 25 | 31 | 27 |
| mmu-miR-6934-5p | 23 | 16 | 24 | 25 |
| mmu-miR-6935-3p | 1 | 4 | 4 | 0 |
| mmu-miR-6935-5p | 18 | 15 | 23 | 4 |
| mmu-miR-6936-3p | 20 | 14 | 17 | 5 |
| mmu-miR-6936-5p | 20 | 18 | 18 | 10 |
| mmu-miR-6937-3p | 4 | 10 | 8 | 3 |
| mmu-miR-6937-5p | 816 | 3,236 | 1,839 | 1,668 |
| mmu-miR-6938-3p | 7 | 80 | 13 | 19 |
| mmu-miR-6938-5p | 8 | 8 | 6 | 2 |
| mmu-miR-6939-3p | 12 | 14 | 10 | 7 |
| mmu-miR-6939-5p | 31 | 30 | 32 | 38 |
| mmu-miR-6940-3p | 4 | 7 | 6 | 4 |
| mmu-miR-6940-5p | 2 | 13 | 6 | 3 |
| mmu-miR-6941-3p | 12 | 13 | 3 | 7 |
| mmu-miR-6941-5p | 10 | 8 | 7 | 7 |
| mmu-miR-6942-3p | 1 | 2 | 0 | 5 |
| mmu-miR-6942-5p | 21 | 118 | 30 | 39 |
| mmu-miR-6943-3p | 2 | 4 | 3 | 3 |
| mmu-miR-6943-5p | 0 | 2 | 0 | 5 |
| mmu-miR-6944-3p | 0 | 4 | 0 | 0 |
| mmu-miR-6944-5p | 251 | 1,887 | 427 | 294 |
| mmu-miR-6945-3p | 1 | 2 | 6 | 0 |
| mmu-miR-6945-5p | 106 | 143 | 133 | 92 |
| mmu-miR-6946-3p | 0 | 17 | 17 | 36 |
| mmu-miR-6946-5p | 0 | 35 | 22 | 20 |
| mmu-miR-6947-3p | 15 | 19 | 16 | 98 |
| mmu-miR-6947-5p | 14 | 33 | 17 | 27 |
| mmu-miR-6948-3p | 0 | 5 | 0 | 11 |
| mmu-miR-6948-5p | 0 | 4 | 5 | 3 |
| mmu-miR-6949-3p | 0 | 0 | 0 | 12 |
| mmu-miR-6949-5p | 0 | 11 | 1 | 5 |
| mmu-miR-6950-3p | 0 | 4 | 1 | 5 |
| mmu-miR-6950-5p | 2 | 14 | 14 | 17 |
| mmu-miR-6951-3p | 0 | 0 | 0 | 0 |
| mmu-miR-6951-5p | 0 | 0 | 0 | 0 |
| mmu-miR-6952-3p | 8 | 7 | 11 | 3 |
| mmu-miR-6952-5p | 5 | 8 | 5 | 8 |
| mmu-miR-6953-3p | 6 | 8 | 8 | 8 |
| mmu-miR-6953-5p | 2 | 1 | 0 | 7 |
| mmu-miR-6954-3p | 1 | 4 | 4 | 8 |
| mmu-miR-6954-5p | 1 | 88 | 29 | 40 |
| mmu-miR-6955-3p | 2 | 3 | 2 | 5 |
| mmu-miR-6955-5p | 10 | 15 | 12 | 17 |
| mmu-miR-6956-3p | 3 | 0 | 1 | 8 |
| mmu-miR-6956-5p | 29 | 45 | 72 | 19 |
| mmu-miR-6957-3p | 0 | 0 | 7 | 4 |
| mmu-miR-6957-5p | 5 | 11 | 45 | 40 |
| mmu-miR-6958-3p | 5 | 4 | 11 | 22 |
| mmu-miR-6958-5p | 8 | 19 | 10 | 18 |
| mmu-miR-6959-3p | 22 | 19 | 15 | 76 |
| mmu-miR-6959-5p | 27 | 62 | 34 | 28 |
| mmu-miR-6960-3p | 0 | 3 | 3 | 17 |
| mmu-miR-6960-5p | 0 | 0 | 0 | 3 |
| mmu-miR-6961-3p | 31 | 0 | 0 | 5 |
| mmu-miR-6961-5p | 2 | 21 | 12 | 17 |
| mmu-miR-6962-3p | 0 | 2 | 0 | 2 |
| mmu-miR-6962-5p | 10 | 5 | 2 | 3 |
| mmu-miR-6963-3p | 7 | 9 | 4 | 1 |
| mmu-miR-6963-5p | 32 | 44 | 28 | 31 |
| mmu-miR-6964-3p | 0 | 0 | 0 | 0 |
| mmu-miR-6964-5p | 5 | 0 | 2 | 4 |
| mmu-miR-6965-3p | 307 | 593 | 433 | 1,267 |
| mmu-miR-6965-5p | 2,074 | 1,100 | 2,457 | 998 |
| mmu-miR-6966-3p | 11 | 1 | 5 | 1 |
| mmu-miR-6966-5p | 13 | 3 | 9 | 2 |
| mmu-miR-6967-3p | 2 | 0 | 0 | 0 |
| mmu-miR-6967-5p | 3 | 0 | 0 | 0 |
| mmu-miR-6968-3p | 20 | 7 | 21 | 2 |
| mmu-miR-6968-5p | 233 | 316 | 768 | 468 |
| mmu-miR-6969-3p | 5 | 0 | 4 | 1 |
| mmu-miR-6969-5p | 3 | 0 | 0 | 0 |
| mmu-miR-6970-3p | 26 | 21 | 22 | 12 |
| mmu-miR-6970-5p | 146 | 351 | 230 | 150 |
| mmu-miR-6971-3p | 7 | 5 | 13 | 8 |
| mmu-miR-6971-5p | 245 | 369 | 177 | 100 |
| mmu-miR-6972-3p | 2 | 0 | 2 | 0 |
| mmu-miR-6972-5p | 27 | 24 | 36 | 20 |
| mmu-miR-6973a-3p | 16 | 6 | 14 | 15 |
| mmu-miR-6973a-5p | 131 | 190 | 95 | 73 |
| mmu-miR-6973b-3p | 2 | 0 | 5 | 2 |
| mmu-miR-6973b-5p | 59 | 136 | 41 | 26 |
| mmu-miR-6974-3p | 6 | 4 | 10 | 8 |
| mmu-miR-6974-5p | 21 | 16 | 29 | 61 |
| mmu-miR-6975-3p | 3 | 8 | 6 | 4 |
| mmu-miR-6975-5p | 34 | 135 | 38 | 36 |
| mmu-miR-6976-3p | 17 | 11 | 19 | 6 |
| mmu-miR-6976-5p | 0 | 0 | 0 | 0 |
| mmu-miR-6977-3p | 21 | 22 | 16 | 14 |
| mmu-miR-6977-5p | 17 | 15 | 13 | 9 |
| mmu-miR-6978-3p | 14 | 13 | 9 | 11 |
| mmu-miR-6978-5p | 17 | 20 | 18 | 9 |
| mmu-miR-6979-3p | 11 | 10 | 16 | 10 |
| mmu-miR-6979-5p | 26 | 20 | 24 | 14 |
| mmu-miR-6980-3p | 3 | 4 | 7 | 2 |
| mmu-miR-6980-5p | 12 | 84 | 31 | 29 |
| mmu-miR-6981-3p | 2 | 2 | 4 | 5 |
| mmu-miR-6981-5p | 210 | 719 | 408 | 182 |
| mmu-miR-6982-3p | 14 | 12 | 25 | 6 |
| mmu-miR-6982-5p | 24 | 22 | 16 | 11 |
| mmu-miR-6983-3p | 0 | 0 | 0 | 0 |
| mmu-miR-6983-5p | 1 | 5 | 0 | 0 |
| mmu-miR-6984-3p | 0 | 4 | 6 | 11 |
| mmu-miR-6984-5p | 11 | 34 | 17 | 6 |
| mmu-miR-6985-3p | 6 | 8 | 9 | 13 |
| mmu-miR-6985-5p | 10 | 14 | 8 | 12 |
| mmu-miR-6986-3p | 3 | 7 | 2 | 4 |
| mmu-miR-6986-5p | 0 | 3 | 3 | 0 |
| mmu-miR-6987-3p | 0 | 3 | 7 | 0 |
| mmu-miR-6987-5p | 32 | 54 | 43 | 47 |
| mmu-miR-6988-3p | 48 | 23 | 26 | 18 |
| mmu-miR-6988-5p | 21 | 23 | 15 | 4 |
| mmu-miR-6989-3p | 8 | 8 | 8 | 3 |
| mmu-miR-6989-5p | 31 | 50 | 48 | 25 |
| mmu-miR-6990-3p | 21 | 25 | 21 | 26 |
| mmu-miR-6990-5p | 157 | 146 | 48 | 143 |
| mmu-miR-6991-3p | 2 | 4 | 3 | 7 |
| mmu-miR-6991-5p | 23 | 23 | 16 | 8 |
| mmu-miR-6992-3p | 0 | 3 | 0 | 1 |
| mmu-miR-6992-5p | 5 | 3 | 1 | 0 |
| mmu-miR-6993-3p | 3 | 1 | 3 | 0 |
| mmu-miR-6993-5p | 13 | 13 | 9 | 5 |
| mmu-miR-6994-3p | 2 | 2 | 1 | 0 |
| mmu-miR-6994-5p | 6 | 12 | 10 | 0 |
| mmu-miR-6995-3p | 7 | 12 | 9 | 5 |
| mmu-miR-6995-5p | 12 | 16 | 6 | 9 |
| mmu-miR-6996-3p | 2 | 6 | 6 | 1 |
| mmu-miR-6996-5p | 46 | 24 | 52 | 39 |
| mmu-miR-6997-3p | 0 | 3 | 0 | 0 |
| mmu-miR-6997-5p | 18 | 28 | 19 | 19 |
| mmu-miR-6998-3p | 17 | 12 | 11 | 8 |
| mmu-miR-6998-5p | 3 | 8 | 2 | 16 |
| mmu-miR-6999-3p | 0 | 0 | 0 | 5 |
| mmu-miR-6999-5p | 14 | 15 | 12 | 20 |
| mmu-miR-7000-3p | 10 | 13 | 18 | 18 |
| mmu-miR-7000-5p | 0 | 0 | 1 | 1 |
| mmu-miR-7001-3p | 2 | 10 | 4 | 7 |
| mmu-miR-7001-5p | 3 | 7 | 6 | 15 |
| mmu-miR-7002-3p | 0 | 5 | 3 | 6 |
| mmu-miR-7002-5p | 2 | 12 | 2 | 14 |
| mmu-miR-7003-3p | 0 | 4 | 0 | 8 |
| mmu-miR-7003-5p | 34 | 51 | 22 | 35 |
| mmu-miR-7004-3p | 9 | 11 | 10 | 16 |
| mmu-miR-7004-5p | 2 | 7 | 5 | 4 |
| mmu-miR-7005-3p | 0 | 5 | 2 | 7 |
| mmu-miR-7005-5p | 391 | 1,026 | 563 | 343 |
| mmu-miR-7006-3p | 0 | 4 | 6 | 0 |
| mmu-miR-7006-5p | 1 | 8 | 2 | 9 |
| mmu-miR-7007-3p | 0 | 3 | 0 | 8 |
| mmu-miR-7007-5p | 13 | 41 | 29 | 32 |
| mmu-miR-7008-3p | 1 | 14 | 11 | 26 |
| mmu-miR-7008-5p | 1 | 21 | 8 | 18 |
| mmu-miR-7009-3p | 1 | 11 | 12 | 16 |
| mmu-miR-7009-5p | 19 | 43 | 24 | 46 |
| mmu-miR-7010-3p | 37 | 32 | 40 | 58 |
| mmu-miR-7010-5p | 0 | 4 | 0 | 7 |
| mmu-miR-7011-3p | 4 | 11 | 10 | 7 |
| mmu-miR-7011-5p | 209 | 858 | 233 | 170 |
| mmu-miR-7012-3p | 12 | 21 | 9 | 2 |
| mmu-miR-7012-5p | 10 | 30 | 14 | 9 |
| mmu-miR-7013-3p | 3 | 5 | 15 | 4 |
| mmu-miR-7013-5p | 2 | 2 | 1 | 3 |
| mmu-miR-7014-3p | 12 | 10 | 19 | 44 |
| mmu-miR-7014-5p | 20 | 20 | 16 | 18 |
| mmu-miR-7015-3p | 0 | 2 | 3 | 0 |
| mmu-miR-7015-5p | 2 | 7 | 3 | 2 |
| mmu-miR-7016-3p | 6 | 10 | 16 | 7 |
| mmu-miR-7016-5p | 22 | 47 | 37 | 34 |
| mmu-miR-7017-3p | 13 | 7 | 7 | 11 |
| mmu-miR-7017-5p | 3 | 10 | 4 | 4 |
| mmu-miR-7018-3p | 10 | 9 | 13 | 4 |
| mmu-miR-7018-5p | 34 | 29 | 42 | 33 |
| mmu-miR-7019-3p | 9 | 9 | 7 | 10 |
| mmu-miR-7019-5p | 29 | 85 | 31 | 88 |
| mmu-miR-7020-3p | 0 | 3 | 1 | 0 |
| mmu-miR-7020-5p | 25 | 97 | 26 | 38 |
| mmu-miR-7021-3p | 0 | 1 | 3 | 9 |
| mmu-miR-7021-5p | 0 | 0 | 0 | 0 |
| mmu-miR-7022-3p | 208 | 105 | 151 | 51 |
| mmu-miR-7022-5p | 7 | 19 | 12 | 10 |
| mmu-miR-7023-3p | 24 | 12 | 25 | 9 |
| mmu-miR-7023-5p | 20 | 11 | 8 | 6 |
| mmu-miR-7024-3p | 19 | 3 | 11 | 3 |
| mmu-miR-7024-5p | 15 | 8 | 8 | 4 |
| mmu-miR-7025-3p | 29 | 15 | 22 | 9 |
| mmu-miR-7025-5p | 32 | 7 | 12 | 1 |
| mmu-miR-7026-3p | 5 | 0 | 4 | 0 |
| mmu-miR-7026-5p | 6 | 0 | 0 | 0 |
| mmu-miR-7027-3p | 17 | 8 | 20 | 11 |
| mmu-miR-7027-5p | 23 | 3 | 11 | 10 |
| mmu-miR-7028-3p | 202 | 129 | 183 | 49 |
| mmu-miR-7028-5p | 14,182 | 33,686 | 2,686 | 22,084 |
| mmu-miR-7029-3p | 11 | 3 | 11 | 0 |
| mmu-miR-7029-5p | 191 | 102 | 91 | 147 |
| mmu-miR-7030-3p | 4 | 0 | 3 | 2 |
| mmu-miR-7030-5p | 13 | 3 | 14 | 2 |
| mmu-miR-7031-3p | 13 | 2 | 11 | 1 |
| mmu-miR-7031-5p | 24 | 10 | 16 | 4 |
| mmu-miR-7032-3p | 15 | 4 | 13 | 5 |
| mmu-miR-7032-5p | 20 | 5 | 15 | 2 |
| mmu-miR-7033-3p | 8 | 3 | 10 | 0 |
| mmu-miR-7033-5p | 859 | 814 | 354 | 493 |
| mmu-miR-7034-3p | 8 | 2 | 5 | 0 |
| mmu-miR-7034-5p | 179 | 126 | 99 | 75 |
| mmu-miR-7035-3p | 20 | 18 | 21 | 7 |
| mmu-miR-7035-5p | 22 | 18 | 14 | 10 |
| mmu-miR-7036a-3p | 14 | 8 | 10 | 5 |
| mmu-miR-7036a-5p | 26 | 473 | 42 | 27 |
| mmu-miR-7036b-3p | 21 | 16 | 20 | 8 |
| mmu-miR-7036b-5p | 16 | 9 | 11 | 12 |
| mmu-miR-7037-3p | 7 | 2 | 1 | 1 |
| mmu-miR-7037-5p | 5 | 0 | 4 | 0 |
| mmu-miR-7038-3p | 15 | 4 | 20 | 8 |
| mmu-miR-7038-5p | 49 | 152 | 84 | 44 |
| mmu-miR-7039-3p | 6 | 2 | 8 | 4 |
| mmu-miR-7039-5p | 14 | 11 | 12 | 6 |
| mmu-miR-7040-3p | 3 | 8 | 7 | 3 |
| mmu-miR-7040-5p | 25 | 53 | 24 | 17 |
| mmu-miR-7041-3p | 0 | 1 | 1 | 3 |
| mmu-miR-7041-5p | 0 | 0 | 0 | 0 |
| mmu-miR-7042-3p | 0 | 0 | 0 | 3 |
| mmu-miR-7042-5p | 27 | 30 | 35 | 34 |
| mmu-miR-7043-3p | 1 | 2 | 1 | 6 |
| mmu-miR-7043-5p | 1 | 21 | 14 | 12 |
| mmu-miR-7044-3p | 14 | 11 | 14 | 13 |
| mmu-miR-7044-5p | 435 | 684 | 529 | 751 |
| mmu-miR-7045-3p | 60 | 77 | 205 | 15 |
| mmu-miR-7045-5p | 508 | 2,144 | 645 | 463 |
| mmu-miR-7046-3p | 24 | 21 | 31 | 4 |
| mmu-miR-7046-5p | 111 | 171 | 100 | 78 |
| mmu-miR-7047-3p | 8 | 16 | 10 | 1 |
| mmu-miR-7047-5p | 621 | 2,692 | 1,490 | 1,279 |
| mmu-miR-7048-3p | 0 | 4 | 0 | 0 |
| mmu-miR-7048-5p | 42 | 56 | 40 | 25 |
| mmu-miR-7049-3p | 61 | 18 | 34 | 16 |
| mmu-miR-7049-5p | 16 | 25 | 16 | 34 |
| mmu-miR-7050-3p | 1 | 8 | 2 | 1 |
| mmu-miR-7050-5p | 67 | 201 | 59 | 49 |
| mmu-miR-7051-3p | 3 | 8 | 5 | 3 |
| mmu-miR-7051-5p | 2 | 20 | 7 | 8 |
| mmu-miR-7052-3p | 24 | 22 | 40 | 5 |
| mmu-miR-7052-5p | 12 | 29 | 27 | 23 |
| mmu-miR-7053-3p | 3 | 5 | 7 | 0 |
| mmu-miR-7053-5p | 27 | 31 | 38 | 37 |
| mmu-miR-7054-3p | 2 | 8 | 9 | 1 |
| mmu-miR-7054-5p | 2 | 4 | 1 | 0 |
| mmu-miR-7055-3p | 0 | 5 | 7 | 0 |
| mmu-miR-7055-5p | 0 | 1 | 0 | 0 |
| mmu-miR-7056-3p | 8 | 10 | 2 | 8 |
| mmu-miR-7056-5p | 142 | 297 | 466 | 609 |
| mmu-miR-7057-3p | 0 | 3 | 0 | 2 |
| mmu-miR-7057-5p | 0 | 0 | 0 | 4 |
| mmu-miR-7058-3p | 89 | 82 | 102 | 122 |
| mmu-miR-7058-5p | 4 | 8 | 3 | 16 |
| mmu-miR-7059-3p | 0 | 4 | 0 | 9 |
| mmu-miR-7059-5p | 0 | 7 | 0 | 12 |
| mmu-miR-7060-3p | 0 | 3 | 0 | 3 |
| mmu-miR-7060-5p | 0 | 7 | 0 | 3 |
| mmu-miR-7061-3p | 0 | 0 | 0 | 0 |
| mmu-miR-7061-5p | 0 | 0 | 0 | 0 |
| mmu-miR-7062-3p | 0 | 5 | 3 | 1 |
| mmu-miR-7062-5p | 0 | 5 | 0 | 5 |
| mmu-miR-7063-3p | 2 | 9 | 10 | 9 |
| mmu-miR-7063-5p | 43 | 86 | 57 | 144 |
| mmu-miR-7064-3p | 0 | 18 | 10 | 16 |
| mmu-miR-7064-5p | 0 | 0 | 0 | 0 |
| mmu-miR-7065-3p | 0 | 0 | 0 | 3 |
| mmu-miR-7065-5p | 1 | 12 | 0 | 55 |
| mmu-miR-7066-3p | 5 | 19 | 28 | 22 |
| mmu-miR-7066-5p | 0 | 4 | 0 | 8 |
| mmu-miR-7067-3p | 0 | 12 | 6 | 6 |
| mmu-miR-7067-5p | 6 | 17 | 4 | 13 |
| mmu-miR-7068-3p | 0 | 15 | 7 | 20 |
| mmu-miR-7068-5p | 0 | 9 | 1 | 17 |
| mmu-miR-7069-3p | 0 | 12 | 5 | 17 |
| mmu-miR-7069-5p | 123 | 103 | 88 | 70 |
| mmu-miR-7070-3p | 1 | 12 | 3 | 12 |
| mmu-miR-7070-5p | 138 | 108 | 48 | 31 |
| mmu-miR-7071-3p | 12 | 11 | 23 | 10 |
| mmu-miR-7071-5p | 15 | 18 | 17 | 12 |
| mmu-miR-7072-3p | 0 | 3 | 7 | 3 |
| mmu-miR-7072-5p | 149 | 87 | 111 | 101 |
| mmu-miR-7073-3p | 0 | 2 | 0 | 0 |
| mmu-miR-7073-5p | 0 | 0 | 0 | 0 |
| mmu-miR-7074-3p | 5 | 5 | 7 | 5 |
| mmu-miR-7074-5p | 2 | 3 | 8 | 3 |
| mmu-miR-7075-3p | 0 | 0 | 0 | 1 |
| mmu-miR-7075-5p | 5 | 42 | 26 | 27 |
| mmu-miR-7076-3p | 0 | 1 | 6 | 0 |
| mmu-miR-7076-5p | 0 | 0 | 1 | 4 |
| mmu-miR-7077-3p | 22 | 21 | 24 | 60 |
| mmu-miR-7077-5p | 4 | 4 | 6 | 11 |
| mmu-miR-7078-3p | 0 | 0 | 0 | 0 |
| mmu-miR-7078-5p | 14 | 14 | 10 | 16 |
| mmu-miR-7079-3p | 0 | 0 | 0 | 0 |
| mmu-miR-7079-5p | 4 | 5 | 1 | 10 |
| mmu-miR-7080-3p | 13 | 3 | 22 | 7 |
| mmu-miR-7080-5p | 13 | 22 | 20 | 20 |
| mmu-miR-7081-3p | 0 | 0 | 1 | 1 |
| mmu-miR-7081-5p | 172 | 557 | 100 | 842 |
| mmu-miR-7082-3p | 23 | 14 | 23 | 7 |
| mmu-miR-7082-5p | 1,806 | 2,419 | 3,284 | 1,750 |
| mmu-miR-7083-3p | 27 | 5 | 15 | 8 |
| mmu-miR-7083-5p | 53 | 8 | 22 | 17 |
| mmu-miR-7084-3p | 10 | 3 | 14 | 0 |
| mmu-miR-7084-5p | 3 | 0 | 1 | 0 |
| mmu-miR-7085-3p | 16 | 3 | 15 | 1 |
| mmu-miR-7085-5p | 112 | 388 | 220 | 192 |
| mmu-miR-7086-3p | 1 | 0 | 0 | 0 |
| mmu-miR-7086-5p | 13 | 9 | 8 | 0 |
| mmu-miR-7087-3p | 24 | 4 | 16 | 3 |
| mmu-miR-7087-5p | 33 | 11 | 14 | 20 |
| mmu-miR-7088-3p | 3 | 0 | 0 | 2 |
| mmu-miR-7088-5p | 103 | 81 | 77 | 53 |
| mmu-miR-7089-3p | 12 | 0 | 6 | 1 |
| mmu-miR-7089-5p | 18 | 1 | 11 | 4 |
| mmu-miR-7090-3p | 2 | 0 | 0 | 0 |
| mmu-miR-7090-5p | 12 | 0 | 5 | 0 |
| mmu-miR-7091-3p | 4 | 0 | 2 | 0 |
| mmu-miR-7091-5p | 4 | 0 | 3 | 0 |
| mmu-miR-7092-3p | 1 | 0 | 0 | 0 |
| mmu-miR-7092-5p | 2 | 0 | 0 | 0 |
| mmu-miR-7093-3p | 4 | 0 | 2 | 1 |
| mmu-miR-7093-5p | 3 | 0 | 0 | 0 |
| mmu-miR-7094-1-5p | 6 | 1 | 3 | 0 |
| mmu-miR-7094-3p | 2 | 0 | 2 | 0 |
| mmu-miR-7094b-2-5p | 4 | 0 | 0 | 2 |
| mmu-miR-7115-3p | 23 | 31 | 54 | 8 |
| mmu-miR-7115-5p | 2 | 0 | 2 | 4 |
| mmu-miR-7116-3p | 0 | 0 | 3 | 1 |
| mmu-miR-7116-5p | 10 | 50 | 0 | 0 |
| mmu-miR-7117-3p | 2 | 2 | 4 | 2 |
| mmu-miR-7117-5p | 3 | 1 | 2 | 2 |
| mmu-miR-7118-3p | 1 | 2 | 0 | 0 |
| mmu-miR-7118-5p | 93 | 423 | 118 | 81 |
| mmu-miR-7119-3p | 0 | 0 | 0 | 0 |
| mmu-miR-7119-5p | 3 | 3 | 75 | 8 |
| mmu-miR-7210-3p | 0 | 0 | 0 | 0 |
| mmu-miR-7210-5p | 0 | 3 | 4 | 2 |
| mmu-miR-7211-3p | 10 | 21 | 8 | 6 |
| mmu-miR-7211-5p | 34 | 34 | 28 | 23 |
| mmu-miR-7212-3p | 1 | 4 | 9 | 3 |
| mmu-miR-7212-5p | 4 | 14 | 2 | 1 |
| mmu-miR-7213-3p | 6 | 11 | 10 | 1 |
| mmu-miR-7213-5p | 0 | 2 | 0 | 0 |
| mmu-miR-7214-3p | 2 | 6 | 6 | 0 |
| mmu-miR-7214-5p | 0 | 0 | 0 | 0 |
| mmu-miR-7215-3p | 13 | 24 | 15 | 11 |
| mmu-miR-7215-5p | 1 | 5 | 0 | 0 |
| mmu-miR-7216-3p | 0 | 0 | 0 | 0 |
| mmu-miR-7216-5p | 24 | 33 | 26 | 28 |
| mmu-miR-7217-3p | 4 | 9 | 2 | 4 |
| mmu-miR-7217-5p | 14 | 11 | 19 | 3 |
| mmu-miR-7218-3p | 33 | 58 | 22 | 6 |
| mmu-miR-7218-5p | 3 | 6 | 2 | 2 |
| mmu-miR-7219-3p | 0 | 2 | 0 | 2 |
| mmu-miR-7219-5p | 0 | 1 | 0 | 0 |
| mmu-miR-7220-3p | 7 | 6 | 3 | 0 |
| mmu-miR-7220-5p | 2 | 3 | 0 | 0 |
| mmu-miR-7221-3p | 277 | 2,539 | 791 | 491 |
| mmu-miR-7221-5p | 8 | 4 | 6 | 4 |
| mmu-miR-7222-3p | 39 | 38 | 29 | 28 |
| mmu-miR-7222-5p | 1 | 8 | 2 | 4 |
| mmu-miR-7223-3p | 2 | 7 | 0 | 3 |
| mmu-miR-7223-5p | 0 | 5 | 0 | 8 |
| mmu-miR-7224-3p | 23 | 20 | 16 | 35 |
| mmu-miR-7224-5p | 6 | 11 | 6 | 2 |
| mmu-miR-7225-3p | 13 | 15 | 22 | 12 |
| mmu-miR-7225-5p | 0 | 3 | 0 | 5 |
| mmu-miR-7226-3p | 0 | 1 | 0 | 7 |
| mmu-miR-7226-5p | 0 | 0 | 0 | 0 |
| mmu-miR-7227-3p | 1 | 5 | 0 | 3 |
| mmu-miR-7227-5p | 0 | 0 | 0 | 0 |
| mmu-miR-7228-3p | 0 | 2 | 0 | 13 |
| mmu-miR-7228-5p | 0 | 3 | 0 | 14 |
| mmu-miR-7229-3p | 0 | 1 | 0 | 0 |
| mmu-miR-7229-5p | 0 | 4 | 0 | 0 |
| mmu-miR-7230-3p | 0 | 0 | 0 | 1 |
| mmu-miR-7230-5p | 0 | 1 | 0 | 1 |
| mmu-miR-7231-3p | 0 | 2 | 0 | 7 |
| mmu-miR-7231-5p | 0 | 53 | 5 | 26 |
| mmu-miR-7232-3p | 0 | 9 | 0 | 14 |
| mmu-miR-7232-5p | 0 | 0 | 0 | 0 |
| mmu-miR-7233-3p | 0 | 0 | 0 | 4 |
| mmu-miR-7233-5p | 41 | 32 | 31 | 61 |
| mmu-miR-7234-3p | 0 | 4 | 0 | 6 |
| mmu-miR-7234-5p | 0 | 0 | 0 | 0 |
| mmu-miR-7235-3p | 8 | 34 | 37 | 50 |
| mmu-miR-7235-5p | 309 | 682 | 256 | 234 |
| mmu-miR-7236-3p | 0 | 2 | 0 | 7 |
| mmu-miR-7236-5p | 0 | 3 | 0 | 4 |
| mmu-miR-7237-3p | 0 | 7 | 0 | 17 |
| mmu-miR-7237-5p | 11 | 9 | 3 | 15 |
| mmu-miR-7238-3p | 0 | 0 | 0 | 5 |
| mmu-miR-7238-5p | 24 | 25 | 17 | 10 |
| mmu-miR-7239-3p | 165 | 363 | 310 | 2,851 |
| mmu-miR-7239-5p | 2 | 2 | 7 | 4 |
| mmu-miR-7240-3p | 0 | 3 | 0 | 1 |
| mmu-miR-7240-5p | 158 | 358 | 216 | 117 |
| mmu-miR-7241-3p | 0 | 22 | 3 | 0 |
| mmu-miR-7241-5p | 0 | 0 | 0 | 0 |
| mmu-miR-7242-3p | 26 | 16 | 12 | 42 |
| mmu-miR-7242-5p | 6 | 9 | 5 | 7 |
| mmu-miR-7243-3p | 3 | 0 | 3 | 0 |
| mmu-miR-7243-5p | 0 | 0 | 0 | 0 |
| mmu-miR-7578 | 59 | 7 | 27 | 6 |
| mmu-miR-7646-3p | 8 | 0 | 2 | 0 |
| mmu-miR-7646-5p | 3 | 0 | 2 | 0 |
| mmu-miR-7647-3p | 13 | 7 | 16 | 3 |
| mmu-miR-7647-5p | 2 | 0 | 2 | 0 |
| mmu-miR-7648-3p | 73 | 57 | 58 | 20 |
| mmu-miR-7648-5p | 339 | 291 | 141 | 279 |
| mmu-miR-7649-3p | 6 | 0 | 3 | 0 |
| mmu-miR-7649-5p | 5 | 0 | 2 | 0 |
| mmu-miR-7650-3p | 0 | 0 | 0 | 0 |
| mmu-miR-7650-5p | 5 | 0 | 0 | 0 |
| mmu-miR-7651-3p | 1 | 0 | 0 | 0 |
| mmu-miR-7651-5p | 8 | 6 | 9 | 1 |
| mmu-miR-7652-3p | 7 | 1 | 0 | 0 |
| mmu-miR-7652-5p | 4 | 2 | 2 | 0 |
| mmu-miR-7653-3p | 12 | 4 | 21 | 0 |
| mmu-miR-7653-5p | 149 | 167 | 186 | 70 |
| mmu-miR-7654-3p | 25 | 29 | 23 | 1 |
| mmu-miR-7654-5p | 17 | 15 | 20 | 4 |
| mmu-miR-7655-3p | 11 | 4 | 5 | 1 |
| mmu-miR-7655-5p | 4 | 0 | 1 | 1 |
| mmu-miR-7656-3p | 8 | 5 | 5 | 0 |
| mmu-miR-7656-5p | 1 | 0 | 0 | 0 |
| mmu-miR-7657-3p | 0 | 0 | 0 | 0 |
| mmu-miR-7657-5p | 0 | 0 | 0 | 0 |
| mmu-miR-7658-3p | 11 | 8 | 7 | 1 |
| mmu-miR-7658-5p | 80 | 219 | 141 | 98 |
| mmu-miR-7659-3p | 2 | 0 | 2 | 0 |
| mmu-miR-7659-5p | 2 | 2 | 4 | 0 |
| mmu-miR-7660-3p | 0 | 0 | 0 | 0 |
| mmu-miR-7660-5p | 1 | 2 | 4 | 0 |
| mmu-miR-7661-3p | 20 | 21 | 32 | 9 |
| mmu-miR-7661-5p | 0 | 0 | 0 | 0 |
| mmu-miR-7662-3p | 10 | 4 | 9 | 2 |
| mmu-miR-7662-5p | 9 | 6 | 6 | 0 |
| mmu-miR-7663-3p | 1 | 1 | 1 | 0 |
| mmu-miR-7663-5p | 6 | 7 | 4 | 30 |
| mmu-miR-7664-3p | 0 | 0 | 0 | 0 |
| mmu-miR-7664-5p | 0 | 0 | 0 | 0 |
| mmu-miR-7665-3p | 5 | 4 | 3 | 1 |
| mmu-miR-7665-5p | 38 | 44 | 36 | 55 |
| mmu-miR-7666-3p | 25 | 57 | 27 | 18 |
| mmu-miR-7666-5p | 11 | 13 | 6 | 3 |
| mmu-miR-7667-3p | 7 | 5 | 3 | 0 |
| mmu-miR-7667-5p | 4 | 8 | 4 | 3 |
| mmu-miR-7668-3p | 8 | 6 | 1 | 9 |
| mmu-miR-7668-5p | 0 | 3 | 2 | 0 |
| mmu-miR-7669-3p | 15 | 33 | 22 | 17 |
| mmu-miR-7669-5p | 2 | 5 | 1 | 11 |
| mmu-miR-7670-3p | 0 | 0 | 0 | 0 |
| mmu-miR-7670-5p | 2 | 4 | 3 | 0 |
| mmu-miR-7671-3p | 57 | 124 | 63 | 53 |
| mmu-miR-7671-5p | 8 | 7 | 10 | 1 |
| mmu-miR-7672-3p | 3 | 6 | 3 | 3 |
| mmu-miR-7672-5p | 6 | 5 | 8 | 0 |
| mmu-miR-7673-3p | 2 | 4 | 2 | 0 |
| mmu-miR-7673-5p | 1 | 4 | 0 | 0 |
| mmu-miR-7674-3p | 6 | 12 | 9 | 4 |
| mmu-miR-7674-5p | 46 | 43 | 48 | 98 |
| mmu-miR-7675-3p | 0 | 0 | 0 | 0 |
| mmu-miR-7675-5p | 0 | 3 | 0 | 0 |
| mmu-miR-7676-3p | 12 | 11 | 8 | 10 |
| mmu-miR-7676-5p | 0 | 0 | 0 | 1 |
| mmu-miR-7677-3p | 5 | 7 | 4 | 2 |
| mmu-miR-7677-5p | 14 | 18 | 11 | 13 |
| mmu-miR-7678-3p | 0 | 1 | 0 | 0 |
| mmu-miR-7678-5p | 4 | 3 | 0 | 11 |
| mmu-miR-7679-3p | 15 | 9 | 14 | 8 |
| mmu-miR-7679-5p | 5 | 11 | 5 | 18 |
| mmu-miR-7680-3p | 0 | 1 | 0 | 5 |
| mmu-miR-7680-5p | 0 | 1 | 0 | 1 |
| mmu-miR-7681-3p | 5 | 6 | 6 | 6 |
| mmu-miR-7681-5p | 0 | 6 | 0 | 5 |
| mmu-miR-7682-3p | 9 | 19 | 8 | 18 |
| mmu-miR-7682-5p | 4 | 2 | 1 | 11 |
| mmu-miR-7683-3p | 279 | 221 | 45 | 556 |
| mmu-miR-7683-5p | 0 | 1 | 0 | 8 |
| mmu-miR-7684-3p | 23 | 91 | 53 | 53 |
| mmu-miR-7684-5p | 5 | 17 | 9 | 18 |
| mmu-miR-7685-3p | 0 | 10 | 1 | 3 |
| mmu-miR-7685-5p | 0 | 2 | 0 | 1 |
| mmu-miR-7686-3p | 0 | 5 | 3 | 11 |
| mmu-miR-7686-5p | 1 | 18 | 10 | 18 |
| mmu-miR-7687-3p | 9 | 15 | 21 | 8 |
| mmu-miR-7687-5p | 16 | 11 | 12 | 12 |
| mmu-miR-7688-3p | 0 | 4 | 0 | 1 |
| mmu-miR-7688-5p | 0 | 7 | 0 | 4 |
| mmu-miR-7689-3p | 2 | 12 | 3 | 9 |
| mmu-miR-7689-5p | 0 | 14 | 9 | 29 |
| mmu-miR-8090 | 3 | 7 | 3 | 7 |
| mmu-miR-8091 | 0 | 0 | 0 | 8 |
| mmu-miR-8092 | 2 | 6 | 7 | 10 |
| mmu-miR-8093 | 53 | 39 | 39 | 46 |
| mmu-miR-8094 | 6 | 12 | 6 | 34 |
| mmu-miR-8095 | 2 | 1 | 1 | 1 |
| mmu-miR-8096 | 2 | 0 | 0 | 3 |
| mmu-miR-8097 | 7 | 1 | 4 | 0 |
| mmu-miR-8098 | 7 | 2 | 4 | 1 |
| mmu-miR-8099 | 7 | 16 | 8 | 3 |
| mmu-miR-8100 | 79 | 57 | 58 | 82 |
| mmu-miR-8101 | 80 | 182 | 130 | 49 |
| mmu-miR-8102 | 39 | 65 | 29 | 15 |
| mmu-miR-8103 | 0 | 3 | 0 | 0 |
| mmu-miR-8104 | 24 | 26 | 15 | 19 |
| mmu-miR-8105 | 11 | 7 | 6 | 11 |
| mmu-miR-8106 | 0 | 0 | 0 | 0 |
| mmu-miR-8107 | 11 | 0 | 2 | 0 |
| mmu-miR-8108 | 20 | 37 | 16 | 15 |
| mmu-miR-8109 | 26 | 21 | 27 | 0 |
| mmu-miR-8110 | 197 | 603 | 200 | 187 |
| mmu-miR-8111 | 3 | 0 | 0 | 0 |
| mmu-miR-8112 | 28 | 16 | 15 | 5 |
| mmu-miR-8113 | 9 | 22 | 13 | 14 |
| mmu-miR-8114 | 9 | 7 | 10 | 0 |
| mmu-miR-8115 | 4 | 0 | 0 | 0 |
| mmu-miR-8116 | 1 | 3 | 1 | 1 |
| mmu-miR-8117 | 17 | 10 | 9 | 2 |
| mmu-miR-8118 | 1 | 0 | 0 | 0 |
| mmu-miR-8119 | 21 | 42 | 23 | 18 |
| mmu-miR-8120 | 10 | 2 | 4 | 0 |
| mmu-miR-9768-3p | 1 | 1 | 0 | 0 |
| mmu-miR-9768-5p | 22 | 27 | 15 | 31 |
| mmu-miR-9769-3p | 2 | 3 | 0 | 1 |
| mmu-miR-9769-5p | 2 | 4 | 0 | 20 |

**Supplementary Table 2.** Microarray analysis – miRNA expression in intact telogen skin of during of 8 week-old and 2 year-old mice

| Reporter Name | 8-W-O telogen skin | 2-Y-O telogen skin |
| --- | --- | --- |
| mmu-let-7a-1-3p | 107 | 47 |
| mmu-let-7a-5p | 8,794 | 7,950 |
| mmu-let-7b-3p | 33 | 25 |
| mmu-let-7b-5p | 4,676 | 4,012 |
| mmu-let-7c-5p | 6,793 | 6,997 |
| mmu-let-7d-3p | 86 | 135 |
| mmu-let-7d-5p | 7,538 | 5,547 |
| mmu-let-7e-5p | 2,680 | 780 |
| mmu-let-7f-5p | 8,720 | 6,991 |
| mmu-let-7g-5p | 3,226 | 2,189 |
| mmu-let-7i-5p | 2,152 | 1,944 |
| mmu-let-7j | 1,838 | 1,419 |
| mmu-let-7k | 1,733 | 1,852 |
| mmu-miR-100-5p | 3,093 | 1,323 |
| mmu-miR-101a-3p | 342 | 120 |
| mmu-miR-101b-3p | 123 | 58 |
| mmu-miR-101c | 151 | 28 |
| mmu-miR-103-3p | 1,360 | 1,927 |
| mmu-miR-106a-5p | 600 | 536 |
| mmu-miR-106b-3p | 33 | 23 |
| mmu-miR-106b-5p | 345 | 514 |
| mmu-miR-107-3p | 1,360 | 1,941 |
| mmu-miR-10a-5p | 1,023 | 843 |
| mmu-miR-10b-5p | 2,467 | 1,862 |
| mmu-miR-1187 | 152 | 112 |
| mmu-miR-1188-5p | 37 | 5 |
| mmu-miR-1191b-5p | 44 | 16 |
| mmu-miR-1195 | 1,018 | 1,022 |
| mmu-miR-1224-5p | 871 | 827 |
| mmu-miR-1231-5p | 34 | 18 |
| mmu-miR-1249-3p | 31 | 19 |
| mmu-miR-125a-5p | 1,879 | 2,514 |
| mmu-miR-125b-2-3p | 48 | 27 |
| mmu-miR-125b-5p | 6,128 | 7,168 |
| mmu-miR-126a-3p | 3,440 | 3,059 |
| mmu-miR-127-3p | 929 | 388 |
| mmu-miR-128-3p | 331 | 239 |
| mmu-miR-130a-3p | 865 | 1,101 |
| mmu-miR-130b-3p | 30 | 31 |
| mmu-miR-132-3p | 30 | 88 |
| mmu-miR-133a-3p | 10,207 | 10,224 |
| mmu-miR-133a-5p | 347 | 281 |
| mmu-miR-133b-3p | 9,726 | 9,240 |
| mmu-miR-133b-5p | 18,080 | 1,760 |
| mmu-miR-134-5p | 37 | 34 |
| mmu-miR-136-3p | 63 | 0 |
| mmu-miR-138-5p | 122 | 158 |
| mmu-miR-139-5p | 188 | 198 |
| mmu-miR-140-3p | 1,373 | 2,303 |
| mmu-miR-140-5p | 88 | 44 |
| mmu-miR-141-3p | 348 | 207 |
| mmu-miR-142a-3p | 322 | 1 |
| mmu-miR-142a-5p | 349 | 49 |
| mmu-miR-143-3p | 3,794 | 4,353 |
| mmu-miR-145a-3p | 167 | 113 |
| mmu-miR-145a-5p | 4,926 | 4,703 |
| mmu-miR-145b | 4,271 | 3,102 |
| mmu-miR-146a-5p | 1,624 | 1,869 |
| mmu-miR-146b-5p | 1,114 | 630 |
| mmu-miR-148a-3p | 943 | 481 |
| mmu-miR-148b-3p | 283 | 265 |
| mmu-miR-149-3p | 1,447 | 1,928 |
| mmu-miR-149-5p | 62 | 39 |
| mmu-miR-150-5p | 658 | 395 |
| mmu-miR-151-3p | 132 | 159 |
| mmu-miR-151-5p | 532 | 701 |
| mmu-miR-152-3p | 878 | 786 |
| mmu-miR-154-5p | 74 | 0 |
| mmu-miR-155-5p | 90 | 50 |
| mmu-miR-15a-3p | 40 | 23 |
| mmu-miR-15a-5p | 1,108 | 455 |
| mmu-miR-15b-3p | 39 | 0 |
| mmu-miR-15b-5p | 915 | 1,224 |
| mmu-miR-16-1-3p | 35 | 0 |
| mmu-miR-16-2-3p | 30 | 0 |
| mmu-miR-16-5p | 5,939 | 5,537 |
| mmu-miR-17-3p | 43 | 34 |
| mmu-miR-17-5p | 854 | 1,121 |
| mmu-miR-181a-1-3p | 47 | 0 |
| mmu-miR-181a-5p | 1,534 | 1,869 |
| mmu-miR-181b-5p | 311 | 332 |
| mmu-miR-181c-5p | 411 | 486 |
| mmu-miR-181d-5p | 239 | 178 |
| mmu-miR-182-5p | 694 | 1,332 |
| mmu-miR-183-5p | 414 | 813 |
| mmu-miR-1839-3p | 293 | 255 |
| mmu-miR-1839-5p | 243 | 216 |
| mmu-miR-1843a-5p | 64 | 55 |
| mmu-miR-1843b-5p | 74 | 68 |
| mmu-miR-185-5p | 183 | 234 |
| mmu-miR-186-5p | 196 | 145 |
| mmu-miR-187-3p | 40 | 57 |
| mmu-miR-188-5p | 50 | 42 |
| mmu-miR-1892 | 145 | 138 |
| mmu-miR-1894-3p | 325 | 136 |
| mmu-miR-1895 | 310 | 301 |
| mmu-miR-1897-5p | 50 | 36 |
| mmu-miR-1906 | 36 | 89 |
| mmu-miR-1907 | 739 | 546 |
| mmu-miR-191-5p | 3,262 | 4,368 |
| mmu-miR-192-5p | 59 | 43 |
| mmu-miR-1934-3p | 134 | 142 |
| mmu-miR-193a-3p | 748 | 799 |
| mmu-miR-193b-3p | 420 | 443 |
| mmu-miR-194-5p | 105 | 90 |
| mmu-miR-1949 | 69 | 120 |
| mmu-miR-1956 | 30 | 21 |
| mmu-miR-195a-5p | 3,423 | 3,898 |
| mmu-miR-196a-5p | 152 | 0 |
| mmu-miR-196b-5p | 85 | 0 |
| mmu-miR-1982-5p | 40 | 22 |
| mmu-miR-1983 | 52 | 22 |
| mmu-miR-199a-3p | 3,359 | 2,755 |
| mmu-miR-199a-5p | 1,365 | 1,296 |
| mmu-miR-199b-5p | 1,117 | 380 |
| mmu-miR-19b-3p | 286 | 242 |
| mmu-miR-1a-1-5p | 120 | 38 |
| mmu-miR-1a-3p | 11,191 | 7,348 |
| mmu-miR-1b-5p | 57 | 10 |
| mmu-miR-200a-3p | 1,696 | 1,796 |
| mmu-miR-200b-3p | 1,772 | 1,944 |
| mmu-miR-200b-5p | 33 | 51 |
| mmu-miR-200c-3p | 1,287 | 2,276 |
| mmu-miR-203-3p | 7,312 | 10,177 |
| mmu-miR-203-5p | 74 | 61 |
| mmu-miR-204-5p | 238 | 52 |
| mmu-miR-205-5p | 10,296 | 16,838 |
| mmu-miR-206-3p | 1,523 | 1,657 |
| mmu-miR-208a-5p | 48 | 6 |
| mmu-miR-20a-5p | 1,004 | 1,085 |
| mmu-miR-20b-5p | 442 | 190 |
| mmu-miR-210-3p | 66 | 290 |
| mmu-miR-2137 | 1,359 | 1,653 |
| mmu-miR-214-3p | 2,663 | 3,054 |
| mmu-miR-214-5p | 78 | 88 |
| mmu-miR-218-5p | 201 | 157 |
| mmu-miR-219c-5p | 34 | 22 |
| mmu-miR-21a-5p | 4,304 | 1,854 |
| mmu-miR-221-3p | 832 | 1,121 |
| mmu-miR-222-3p | 447 | 773 |
| mmu-miR-223-3p | 896 | 142 |
| mmu-miR-22-3p | 2,588 | 2,525 |
| mmu-miR-224-5p | 47 | 37 |
| mmu-miR-22-5p | 227 | 226 |
| mmu-miR-23a-3p | 9,284 | 9,872 |
| mmu-miR-23b-3p | 9,077 | 8,921 |
| mmu-miR-24-1-5p | 49 | 12 |
| mmu-miR-24-2-5p | 432 | 423 |
| mmu-miR-24-3p | 9,738 | 10,589 |
| mmu-miR-25-3p | 895 | 822 |
| mmu-miR-26a-5p | 12,238 | 11,763 |
| mmu-miR-26b-5p | 3,582 | 1,754 |
| mmu-miR-27a-3p | 3,255 | 2,228 |
| mmu-miR-27b-3p | 2,292 | 1,997 |
| mmu-miR-2861 | 324 | 619 |
| mmu-miR-28a-3p | 42 | 36 |
| mmu-miR-28a-5p | 180 | 86 |
| mmu-miR-28b | 52 | 0 |
| mmu-miR-28c | 293 | 228 |
| mmu-miR-299a-5p | 131 | 6 |
| mmu-miR-299b-5p | 137 | 11 |
| mmu-miR-29a-3p | 4,186 | 4,180 |
| mmu-miR-29b-3p | 2,037 | 560 |
| mmu-miR-29c-3p | 1,098 | 236 |
| mmu-miR-300-3p | 81 | 13 |
| mmu-miR-3064-3p | 45 | 35 |
| mmu-miR-3068-3p | 532 | 672 |
| mmu-miR-3068-5p | 71 | 39 |
| mmu-miR-3070-2-3p | 302 | 160 |
| mmu-miR-3072-5p | 1,189 | 1,443 |
| mmu-miR-3077-5p | 652 | 220 |
| mmu-miR-3082-5p | 241 | 384 |
| mmu-miR-3084-3p | 71 | 144 |
| mmu-miR-3095-3p | 57 | 15 |
| mmu-miR-30a-3p | 204 | 175 |
| mmu-miR-30a-5p | 3,160 | 2,843 |
| mmu-miR-30b-5p | 4,417 | 3,482 |
| mmu-miR-30c-1-3p | 682 | 261 |
| mmu-miR-30c-2-3p | 106 | 16 |
| mmu-miR-30c-5p | 4,235 | 4,390 |
| mmu-miR-30d-5p | 1,997 | 1,809 |
| mmu-miR-30e-3p | 246 | 118 |
| mmu-miR-30e-5p | 1,756 | 1,156 |
| mmu-miR-30f | 148 | 0 |
| mmu-miR-3102-3p | 34 | 25 |
| mmu-miR-3102-5p.2-5p | 286 | 375 |
| mmu-miR-3110-3p | 48 | 147 |
| mmu-miR-3154 | 93 | 32 |
| mmu-miR-31-5p | 130 | 2,518 |
| mmu-miR-320-3p | 616 | 1,268 |
| mmu-miR-322-3p | 76 | 41 |
| mmu-miR-322-5p | 504 | 131 |
| mmu-miR-32-3p | 110 | 80 |
| mmu-miR-324-3p | 41 | 44 |
| mmu-miR-324-5p | 105 | 172 |
| mmu-miR-328-3p | 52 | 69 |
| mmu-miR-328-5p | 496 | 231 |
| mmu-miR-329-3p | 454 | 400 |
| mmu-miR-331-3p | 77 | 80 |
| mmu-miR-335-5p | 221 | 148 |
| mmu-miR-337-3p | 125 | 7 |
| mmu-miR-337-5p | 97 | 48 |
| mmu-miR-338-3p | 154 | 12 |
| mmu-miR-338-5p | 65 | 50 |
| mmu-miR-339-5p | 40 | 21 |
| mmu-miR-340-3p | 41 | 11 |
| mmu-miR-340-5p | 194 | 13 |
| mmu-miR-341-3p | 104 | 79 |
| mmu-miR-341-5p | 29,972 | 38,658 |
| mmu-miR-342-3p | 677 | 464 |
| mmu-miR-344g-5p | 30 | 19 |
| mmu-miR-345-5p | 87 | 71 |
| mmu-miR-346-3p | 71 | 76 |
| mmu-miR-346-5p | 75 | 211 |
| mmu-miR-3470a | 63 | 38 |
| mmu-miR-3470b | 60 | 55 |
| mmu-miR-3473a | 141 | 71 |
| mmu-miR-3473b | 1,489 | 1,198 |
| mmu-miR-3473e | 1,492 | 903 |
| mmu-miR-3473f | 964 | 451 |
| mmu-miR-3473g | 96 | 116 |
| mmu-miR-3474 | 40 | 25 |
| mmu-miR-34a-5p | 312 | 536 |
| mmu-miR-34c-3p | 86 | 116 |
| mmu-miR-350-3p | 333 | 128 |
| mmu-miR-351-5p | 88 | 59 |
| mmu-miR-3535 | 1,264 | 2,908 |
| mmu-miR-3547-5p | 148 | 175 |
| mmu-miR-3569-5p | 94 | 160 |
| mmu-miR-361-5p | 245 | 352 |
| mmu-miR-3620-5p | 330 | 564 |
| mmu-miR-362-3p | 96 | 19 |
| mmu-miR-362-5p | 107 | 91 |
| mmu-miR-363-5p | 176 | 78 |
| mmu-miR-365-3p | 808 | 109 |
| mmu-miR-369-3p | 37 | 0 |
| mmu-miR-374b-5p | 362 | 125 |
| mmu-miR-374c-5p | 264 | 47 |
| mmu-miR-375-3p | 36 | 32 |
| mmu-miR-376a-3p | 49 | 9 |
| mmu-miR-376b-3p | 138 | 37 |
| mmu-miR-376b-5p | 92 | 2 |
| mmu-miR-376c-3p | 134 | 17 |
| mmu-miR-376c-5p | 70 | 1 |
| mmu-miR-377-3p | 82 | 8 |
| mmu-miR-378a-3p | 2,041 | 2,894 |
| mmu-miR-378a-5p | 148 | 282 |
| mmu-miR-378b | 1,560 | 2,142 |
| mmu-miR-378c | 1,604 | 2,317 |
| mmu-miR-378d | 1,493 | 1,740 |
| mmu-miR-379-3p | 87 | 16 |
| mmu-miR-379-5p | 327 | 114 |
| mmu-miR-381-3p | 86 | 37 |
| mmu-miR-3960 | 1,183 | 1,373 |
| mmu-miR-3962 | 1,902 | 207 |
| mmu-miR-3963 | 1,238 | 888 |
| mmu-miR-3965 | 94 | 0 |
| mmu-miR-3968 | 31 | 19 |
| mmu-miR-3970 | 1,741 | 196 |
| mmu-miR-409-3p | 74 | 34 |
| mmu-miR-409-5p | 49 | 0 |
| mmu-miR-410-3p | 45 | 0 |
| mmu-miR-411-3p | 168 | 22 |
| mmu-miR-411-5p | 247 | 65 |
| mmu-miR-421-3p | 30 | 26 |
| mmu-miR-423-5p | 163 | 277 |
| mmu-miR-425-3p | 36 | 14 |
| mmu-miR-425-5p | 268 | 366 |
| mmu-miR-429-3p | 928 | 1,318 |
| mmu-miR-431-5p | 37 | 10 |
| mmu-miR-434-3p | 279 | 31 |
| mmu-miR-434-5p | 53 | 0 |
| mmu-miR-451a | 713 | 1,523 |
| mmu-miR-455-3p | 167 | 345 |
| mmu-miR-466c-5p | 99 | 41 |
| mmu-miR-466d-3p | 34 | 25 |
| mmu-miR-466f-3p | 485 | 693 |
| mmu-miR-466f-5p | 57 | 30 |
| mmu-miR-466g | 184 | 299 |
| mmu-miR-466h-3p | 340 | 556 |
| mmu-miR-466i-3p | 295 | 308 |
| mmu-miR-466i-5p | 1,123 | 1,984 |
| mmu-miR-466m-3p | 184 | 157 |
| mmu-miR-466m-5p | 161 | 150 |
| mmu-miR-466q | 141 | 175 |
| mmu-miR-467a-3p | 76 | 68 |
| mmu-miR-467b-3p | 247 | 312 |
| mmu-miR-467c-3p | 47 | 26 |
| mmu-miR-467d-3p | 166 | 193 |
| mmu-miR-467e-3p | 32 | 0 |
| mmu-miR-467f | 241 | 289 |
| mmu-miR-467g | 32 | 14 |
| mmu-miR-468-3p | 49 | 34 |
| mmu-miR-483-5p | 248 | 241 |
| mmu-miR-484 | 128 | 297 |
| mmu-miR-485-3p | 45 | 48 |
| mmu-miR-486a-3p | 482 | 310 |
| mmu-miR-486a-5p | 250 | 511 |
| mmu-miR-486b-3p | 48 | 75 |
| mmu-miR-487b-3p | 32 | 16 |
| mmu-miR-489-3p | 49 | 89 |
| mmu-miR-494-3p | 1,509 | 1,106 |
| mmu-miR-495-3p | 51 | 18 |
| mmu-miR-497a-5p | 601 | 771 |
| mmu-miR-500-3p | 159 | 116 |
| mmu-miR-501-3p | 140 | 125 |
| mmu-miR-503-5p | 64 | 22 |
| mmu-miR-504-3p | 48 | 27 |
| mmu-miR-5099 | 3,092 | 4,172 |
| mmu-miR-5100 | 325 | 567 |
| mmu-miR-5107-5p | 1,572 | 781 |
| mmu-miR-5112 | 1,473 | 3,907 |
| mmu-miR-5113 | 37 | 0 |
| mmu-miR-511-3p | 221 | 85 |
| mmu-miR-5119 | 75 | 105 |
| mmu-miR-5121 | 781 | 1,147 |
| mmu-miR-5122 | 71 | 51 |
| mmu-miR-5126 | 3,284 | 3,779 |
| mmu-miR-5128 | 46 | 62 |
| mmu-miR-5130 | 52 | 80 |
| mmu-miR-5132-5p | 73 | 40 |
| mmu-miR-532-3p | 39 | 60 |
| mmu-miR-532-5p | 167 | 234 |
| mmu-miR-539-5p | 47 | 9 |
| mmu-miR-541-5p | 130 | 49 |
| mmu-miR-5622-3p | 32 | 49 |
| mmu-miR-574-3p | 209 | 247 |
| mmu-miR-574-5p | 345 | 434 |
| mmu-miR-615-5p | 200 | 135 |
| mmu-miR-6236 | 124 | 77 |
| mmu-miR-6238 | 1,326 | 343 |
| mmu-miR-6239 | 29,642 | 39,029 |
| mmu-miR-6240 | 793 | 937 |
| mmu-miR-6244 | 41 | 17 |
| mmu-miR-6348 | 176 | 333 |
| mmu-miR-6349 | 130 | 145 |
| mmu-miR-6360 | 179 | 281 |
| mmu-miR-6366 | 96 | 104 |
| mmu-miR-6368 | 36 | 43 |
| mmu-miR-6385 | 62 | 162 |
| mmu-miR-6394 | 34 | 48 |
| mmu-miR-6402 | 715 | 32 |
| mmu-miR-6418-5p | 57 | 25 |
| mmu-miR-6516-5p | 76 | 117 |
| mmu-miR-652-3p | 298 | 762 |
| mmu-miR-652-5p | 45 | 29 |
| mmu-miR-6538 | 453 | 607 |
| mmu-miR-6540-5p | 66 | 34 |
| mmu-miR-664-3p | 62 | 87 |
| mmu-miR-665-3p | 141 | 55 |
| mmu-miR-665-5p | 81 | 90 |
| mmu-miR-668-3p | 1,038 | 288 |
| mmu-miR-669a-3-3p | 35 | 32 |
| mmu-miR-669a-3p | 170 | 208 |
| mmu-miR-669a-5p | 36 | 17 |
| mmu-miR-669b-5p | 38 | 33 |
| mmu-miR-669c-3p | 191 | 163 |
| mmu-miR-669c-5p | 272 | 359 |
| mmu-miR-669e-3p | 72 | 110 |
| mmu-miR-669f-3p | 111 | 144 |
| mmu-miR-669f-5p | 140 | 125 |
| mmu-miR-669k-5p | 57 | 29 |
| mmu-miR-669l-5p | 88 | 27 |
| mmu-miR-669n | 291 | 315 |
| mmu-miR-669o-5p | 69 | 16 |
| mmu-miR-669p-3p | 263 | 351 |
| mmu-miR-671-5p | 127 | 119 |
| mmu-miR-674-3p | 41 | 50 |
| mmu-miR-674-5p | 170 | 243 |
| mmu-miR-676-3p | 110 | 188 |
| mmu-miR-6769b-5p | 32 | 28 |
| mmu-miR-677-3p | 122 | 246 |
| mmu-miR-677-5p | 39 | 1 |
| mmu-miR-678 | 54 | 59 |
| mmu-miR-680 | 45 | 57 |
| mmu-miR-681 | 34 | 75 |
| mmu-miR-6896-3p | 39 | 16 |
| mmu-miR-690 | 22,202 | 18,637 |
| mmu-miR-6906-5p | 43 | 36 |
| mmu-miR-691 | 481 | 78 |
| mmu-miR-6912-5p | 63 | 130 |
| mmu-miR-6923-5p | 34 | 26 |
| mmu-miR-6925-5p | 56 | 29 |
| mmu-miR-6929-3p | 30 | 51 |
| mmu-miR-6931-5p | 1,356 | 472 |
| mmu-miR-6934-3p | 39 | 35 |
| mmu-miR-6937-5p | 669 | 816 |
| mmu-miR-6939-5p | 37 | 31 |
| mmu-miR-6942-5p | 53 | 21 |
| mmu-miR-6944-5p | 301 | 251 |
| mmu-miR-6945-5p | 53 | 106 |
| mmu-miR-6946-5p | 37 | 0 |
| mmu-miR-6947-5p | 39 | 14 |
| mmu-miR-6954-5p | 36 | 1 |
| mmu-miR-696 | 30 | 30 |
| mmu-miR-6963-5p | 54 | 32 |
| mmu-miR-6965-3p | 2,969 | 307 |
| mmu-miR-6965-5p | 898 | 2,074 |
| mmu-miR-6968-5p | 227 | 233 |
| mmu-miR-6970-5p | 105 | 146 |
| mmu-miR-6971-5p | 222 | 245 |
| mmu-miR-6973a-5p | 129 | 131 |
| mmu-miR-6973b-5p | 45 | 59 |
| mmu-miR-6975-5p | 67 | 34 |
| mmu-miR-6980-5p | 42 | 12 |
| mmu-miR-6981-5p | 230 | 210 |
| mmu-miR-6984-3p | 39 | 0 |
| mmu-miR-6987-5p | 51 | 32 |
| mmu-miR-6989-5p | 31 | 31 |
| mmu-miR-6990-5p | 166 | 157 |
| mmu-miR-7003-5p | 31 | 34 |
| mmu-miR-700-3p | 129 | 128 |
| mmu-miR-7005-5p | 253 | 391 |
| mmu-miR-7009-5p | 30 | 19 |
| mmu-miR-7010-3p | 35 | 37 |
| mmu-miR-7011-5p | 194 | 209 |
| mmu-miR-7016-5p | 36 | 22 |
| mmu-miR-7019-5p | 93 | 29 |
| mmu-miR-7020-5p | 70 | 25 |
| mmu-miR-7022-3p | 115 | 208 |
| mmu-miR-7028-3p | 70 | 202 |
| mmu-miR-7028-5p | 27,622 | 14,182 |
| mmu-miR-7029-5p | 171 | 191 |
| mmu-miR-703 | 65 | 65 |
| mmu-miR-7033-5p | 614 | 859 |
| mmu-miR-7034-5p | 125 | 179 |
| mmu-miR-7036a-5p | 43 | 26 |
| mmu-miR-7038-5p | 68 | 49 |
| mmu-miR-7040-5p | 31 | 25 |
| mmu-miR-7042-5p | 35 | 27 |
| mmu-miR-7044-5p | 499 | 435 |
| mmu-miR-7045-3p | 69 | 60 |
| mmu-miR-7045-5p | 476 | 508 |
| mmu-miR-7046-5p | 85 | 111 |
| mmu-miR-7047-5p | 605 | 621 |
| mmu-miR-7048-5p | 44 | 42 |
| mmu-miR-7049-3p | 37 | 61 |
| mmu-miR-705 | 662 | 509 |
| mmu-miR-7050-5p | 76 | 67 |
| mmu-miR-7056-5p | 786 | 142 |
| mmu-miR-7058-3p | 77 | 89 |
| mmu-miR-706 | 214 | 121 |
| mmu-miR-7063-5p | 117 | 43 |
| mmu-miR-7069-5p | 60 | 123 |
| mmu-miR-7070-5p | 40 | 138 |
| mmu-miR-7071-3p | 41 | 12 |
| mmu-miR-7072-5p | 244 | 149 |
| mmu-miR-7075-5p | 40 | 5 |
| mmu-miR-7081-5p | 3,194 | 172 |
| mmu-miR-7082-5p | 3,875 | 1,806 |
| mmu-miR-7085-5p | 221 | 112 |
| mmu-miR-708-5p | 536 | 1,043 |
| mmu-miR-7088-5p | 73 | 103 |
| mmu-miR-709 | 19,540 | 26,074 |
| mmu-miR-711 | 52 | 12 |
| mmu-miR-7118-5p | 117 | 93 |
| mmu-miR-714 | 191 | 161 |
| mmu-miR-718 | 82 | 81 |
| mmu-miR-7221-3p | 214 | 277 |
| mmu-miR-7233-5p | 58 | 41 |
| mmu-miR-7235-3p | 32 | 8 |
| mmu-miR-7235-5p | 245 | 309 |
| mmu-miR-7239-3p | 6,786 | 165 |
| mmu-miR-7240-5p | 119 | 158 |
| mmu-miR-7242-3p | 53 | 26 |
| mmu-miR-744-5p | 199 | 498 |
| mmu-miR-7578 | 36 | 59 |
| mmu-miR-758-5p | 65 | 111 |
| mmu-miR-762 | 988 | 751 |
| mmu-miR-763 | 31 | 40 |
| mmu-miR-7648-3p | 38 | 73 |
| mmu-miR-7648-5p | 455 | 339 |
| mmu-miR-7653-5p | 93 | 149 |
| mmu-miR-7658-5p | 97 | 80 |
| mmu-miR-7665-5p | 76 | 38 |
| mmu-miR-7671-3p | 66 | 57 |
| mmu-miR-7674-5p | 36 | 46 |
| mmu-miR-7683-3p | 285 | 279 |
| mmu-miR-7684-3p | 50 | 23 |
| mmu-miR-770-3p | 74 | 26 |
| mmu-miR-7a-1-3p | 31 | 0 |
| mmu-miR-7a-5p | 65 | 4 |
| mmu-miR-8100 | 49 | 79 |
| mmu-miR-8101 | 61 | 80 |
| mmu-miR-8102 | 30 | 39 |
| mmu-miR-8104 | 35 | 24 |
| mmu-miR-8109 | 37 | 26 |
| mmu-miR-8110 | 199 | 197 |
| mmu-miR-8113 | 32 | 9 |
| mmu-miR-872-3p | 31 | 28 |
| mmu-miR-872-5p | 138 | 86 |
| mmu-miR-877-5p | 38 | 58 |
| mmu-miR-92a-3p | 379 | 922 |
| mmu-miR-92b-3p | 237 | 521 |
| mmu-miR-93-5p | 648 | 1,064 |
| mmu-miR-96-5p | 271 | 40 |
| mmu-miR-9768-5p | 40 | 22 |
| mmu-miR-98-5p | 1,134 | 213 |
| mmu-miR-99a-5p | 3,653 | 3,054 |
| mmu-miR-99b-5p | 731 | 1,046 |

**Supplementary Figure 1. miR-200c does not affect keratinocyte proliferation in vitro.**

**a)** Quantitative analysis of proliferating Ki-67+ cells: proliferation of primary mouse epidermal keratinocytes (PMEKs) is unaffected after transfection with miR-200c mimic (n = 3; mean + SE); **b)** Representative images of Ki-67 immunofluorescence (red) of PMEKs transfected with either miR-200c mimic or negative control scrambled RNA, nuclear staining DAPI (blue); Scale bar: 10 µm;

**c)** FACS analysis: no changes in cell cycle progression through the different phases in HaCaT cells as a result of their transfection with miR-200c mimic compared to negative control scrambled RNA, (n = 3).
